# Supplementary material for: Stakeholders’ Perceptions of How Nurse–Doctor Communication Impacts Patient Care: A Concept Mapping Study
Source: Nurs Rep. 2023 Nov 6;13(4):1607–23. doi: 10.3390/nursrep13040133 (PMC10661264; doi:10.3390/nursrep13040133)
Supplement: Supplementary file 1 [file nursrep-13-00133-s001.zip › nursrep-2581629-supplementary/S2_Complete list of statements.docx]

**General**

1. Patient care needs a multidisciplinary team approach involving doctors, nurses, allied health, social workers, and pastoral care
2. it is good to include a patient in his care if he can speak for himself
3. Doctors and nurses are the forefront of the healthcare practice.
4. Nurses and doctors complement each other as they view and see things differently
5. Nurse-doctor communication is incredibly important in terms of patient care
6. Communication is a key factor to safe and good patient care
7. Communication is one of the important pillars in nursing practice
8. Communication is extremely important for providing high quality care
9. patient outcomes will be better with detailed communication
10. nurses should have skills to report the concerns of a patient with the doctor
11. Nurse-doctor communication can affect either positively or negatively based on the dynamics of the communication
12. Information obtained by nurses and doctors combinedly form a complete story of what is going with the patient
13. Nurse-doctor communication is integral to a deeper understanding of a patient
14. Nurse-doctor communication is very valuable to achieve a proper patient care
15. When doctors and nurses are communicating well, there is cohesion in the care delivery
16. communication between the different members of a health care team is vital
17. It is important nurses understand medical care plan so that they can deliver it
18. very clear communication needs to go back and forth between all the different people
19. Intersectoral communication (with patients, relatives, government line agencies) is important to convey the message for prompt management of outbreaks
20. Nurses form the communication link between the medical team and rest of the disciplines
21. It is important to convey message to all the staff to delegate the care task
22. Nurses and doctors should be on the same page
23. There should be a cohesive healthcare team to ensure a patient is better managed
24. There must be a proper channel for communication between nurses and doctors
25. Everyone should be aware of the policies or guidelines for communication that is based on the priority of patient care
26. Hospitals need to design the communication pathways so that the communication can be the best it needs to be
27. Having forums, team meetings, nurse-doctor meetings where we can talk about patient so that everyone knows
28. Ensure things are attended
29. Disagreements can help pick up small things that sometimes can be missed
30. Nurses feel easy to communicate with the doctors they know
31. Nurse and the doctor should know what each other want (or are doing)
32. Familiarity with the clinician and working with them helps in building rapport and communication
33. Nurses can follow up the missing things recommended by the doctor
34. Nurses help carry out the recommendations made by the doctor
35. Nurses and doctor should understand their professional boundaries
36. Doctors focus on the illness and medicine whereas nurses tend to look at more holistic picture of the patient
37. The doctor needs to know what is happening to his patient even though he is not there in the hospital
38. Having a better understanding of where each other are coming is very important
39. We need to set up the boundaries and know when to help each other
40. doctor gets annoyed due to messages from the nurses which was their part of their care
41. Few nurses do try to play a heroic role – trying to seek doctor’s help straight away
42. care becomes fragmented if there is lack of good communication between the nurse and the doctor
43. Doctor should provide clear instruction to the nurses
44. Doctors should be approachable to the nurses
45. Details of doctors should be readily available at the nursing station
46. Due to apprehension between nurses and doctors, nurses might not feel comfortable in going to the doctor
47. The doctor needs to have allocated time to speak to the nurses
48. At critical points in patient care, information from nursing staff is often very important
49. Nurses should provide accurate and detailed information to the doctors
50. Nurses filter the information going to the doctor
51. Nurses prepare the ground form their observations to explain the doctor
52. Its responsibility of a nurse to make a doctor aware of the patient’s situation and medical condition
53. Medical staff get insight to the patient’s condition from the nursing notes
54. Nurses brief the patient’s concern and condition which helps a doctor to be prepared before seeing a patient
55. nurse needs to be clear communicating back (talk) to the doctor any concerns she has
56. They should openly express the ideas and opinions about the treatment method and everything
57. when adequate information is not conveyed to the doctors, it could negatively impact the care provided
58. A lot of communication between patient and nurses probably never end up getting communicated to the doctor
59. Nurses might have noticed something that the doctors may not notice during the ward rounds
60. Nurses’ communication of a patients’ situation helps a doctor helps take prompt action
61. A good communication will help nurse to act on quickly based on the existing guidelines
62. Nurses do a lot of stuff (small procedures) for doctors
63. Efficiency of nurses helps undertaking a procedure
64. A nurse provides emotional support to the patients
65. Nurses are the eyes, ears, and mouth for the patients
66. Engaging family members is one of the vital roles in nursing practice
67. Families have the right to know about what is happening with their patient
68. It is important for the nurses to know what is going on
69. Deep understanding of the social situation, social issues, and the personality of the patient
70. With a good communication we can identify other problems that concerns a patient
71. with effective communication it is easier to get to the bottom of the problem
72. Having the patients’ best interest at front and centre is really the most important thing.
73. If a nurse believes that a doctor is wrong, she must act on and not just ignore it
74. It is a responsibility of a nurse to stand up if they think if the drug chart is written incorrectly
75. Good communication can give nurses an opportunity to pass on some important information
76. If nurses do not carry out an order, they should not sign on that order
77. These days, nurses are more assertive and often battle for their patients
78. it is important to have assertiveness in communication
79. A doctor can act upon what is best for the patient when they listen to the nurse
80. Nurses have an idea about which allied health professional would be better for the patient
81. Nurses communicate with patient, reassuring the patient of what the patient is expecting
82. Hospital is not the place where nurses always follow the order of the doctors
83. Nurse-doctor communication impact to care a patient under bio-psycho-social framework.
84. Nurses can have idea of what is best for the patient because of being with the patient for most of the time
85. Nurses assist patient from their entry to the hospital
86. The primary contact of the patient is always a nurse.
87. Nurses are always there with the patients
88. Doctors go to see the patient during the ward rounds or whenever the nurse contact us for any complication or other things that we must look for.
89. Communication must be good both ways for the best patient care in a hospital
90. Doctors should value the nursing input and involve them in clinical decision making
91. Patient management can be altered with good communication
92. Doctors need to introduce themselves, their preferences, and expectances better with the people we work with
93. Having a safe happy communication outside of those working hours talking about real interest on people you work with, asking about their life, asking about their interest, sitting down having a meal
94. Having a meal along with nurses in the tearoom helps to get to know them
95. Spending more time with the nurses makes a doctor lot more valuable and stops a lot of problem from happening as it is easier for people to access them
96. Having a teatime with multidisciplinary team go through the list of patients and their plan helps to identify the things needed to be done from different members makes things efficient
97. nurses and doctors should have open area where they write their notes or communal space where can know each other (in their working area)
98. If they see each other at other places, they could greet each other and be on good terms
99. If we have good working relationship, nursing staff are more likely to do things that need to be done urgently
100. Doctors and nurses need to work together as a unit to get the job done
101. It is important for nurses to escalate concerns to facilitate patient review
102. A doctor needs to liaise with the nursing team about implementation of (changes to) plan is important to optimise patient care
103. Doctors need to inform changes in the medication to the nurses
104. nurses and doctors need to make the effort to read what the other one has written
105. Nurses act as patient’s advocate particularly if there are any concerns raised by patients or family members
106. Nurse make the communication between the patient and their family member
107. Because of lack of communication, sometimes patients need to be advocate of themselves
108. There are lot of instances where people may not be conscious enough to say that it is wrong
109. Medical work is a team-based approach
110. Team morale can be influenced by the level of communication between nurses and doctors
111. Lack of communication will decrease personal self-esteem (of a nurse)
112. Nurses are at better placed to raise questions asked when doctors are rushing
113. The tone between the nursing and the medical team can have a massive influence on the way how rest of the staff in the ward communicate
114. change in tone can indicate seriousness or urgency in communication
115. When there are negative interactions between nurses and doctors, it is difficult getting good outcomes for the patient
116. There is a direct impact if doctors can communicate the plan to the nurse
117. If doctors can communicate with the nurses, then there is no delay as things can be clarified instantly
118. Nurse should have courage to question, ask or clarify information from the doctor
119. A nurse should properly question on what is happening around because she has the duty of care to watch that patient
120. A nurse can put forward her concerns and suggestions to the doctor in a way that he/she is not putting any authority on the doctor
121. If a nurse can interact frequently with the doctor with the patient’s condition, it can help patient get managed promptly
122. it makes a difference when nurses are confident of communicating with the doctor to ask something
123. Doctors should ask opinion from the nurses
124. One should value the opinion of the other
125. Doctors should be available to answer the nurses’ queries
126. Easy access to nursing and medical staff will help to discuss patient urgency
127. In aged care setting, doctors are not available at the time when nurses need them
128. Nurses freely asking doctors for something or even pointing out mistakes is important
129. Update each other so that patient is aware what is happening
130. Nurses and doctors have a different area of work, they should be valued given to all the beats of work
131. Doctors came on their round with other doctors, but I did not see any communication between the doctors and the nurses.
132. All we can do is hope that doctors are communicating with nurses at some other locations where you cannot see it
133. You do wonder about whether there was any coordination happens and how good that coordination was
134. There needs to be coordination of care
135. Frequency of care needs to be increased
136. If documentation is carried out at the right place it will not take a long time to find it
137. Standardising hospital forms across hospitals will make doctors confident of documentation
138. Documentation is one of the factors that could improve communication
139. It is of no point if you do not document whatever you talk with the patient
140. There needs to be a lot more of written communication which you can reflect or have a look at
141. workload of a doctor may impact their ability to do their job efficiently

**Communication mode**

1. It should be a three-way conversation with having the inputs from the patients
2. Things that cannot be communicated in a medical chart needs to be verbally communication and hand over
3. Some of the subtle things in patient care need to be communicated verbally rather than in a piece of paper
4. Having an ongoing communication helps to pick up the subtle changes in the patient’s conditions
5. If anything goes wrong, the doctor needs to be informed straight away
6. We can send an email to communicate. For example, sending a photo, which can be saved in the patient profile.
7. We can make a phone call through our internal telephone
8. We need to tell things in a friendly way so that others can take it easily
9. With verbal handover things can be missed out
10. If a verbal communication is not documented, it carries a risk of not being handed over to the next nurse
11. If there is verbal communication, patient care could be potentially rectified due to prompt action
12. a written thing needs to be verbally communicated
13. Patients may have problems when there is no good linkage of handovers between nursing and medical staff
14. Nurse-doctor communication is important to monitor and assess the patient
15. There is a lot of interpersonal issues that arise in the ward that could affect communication
16. Interpersonal relationships are not as important as the patient in front of you
17. In a hostile and abusive environment personal safety comes first
18. There needs to be a degree of psychological safety within an organization to create an environment facilitating the people to bring out the issues
19. We need to feel safe to talk to people
20. With good rapport, a nurse will feel comfortable to come forward to tell their concerns about the patient
21. we cannot get on with everybody at our work, but we can work together to provide a good health care
22. Clear communication in verbal language giving nurses chances to explain
23. nurses and doctors should understand clearly what it is wanted or needed by both sides
24. Directly calling the doctors may work but it can increase interruptions to their work
25. Pagers could potentially provide short messages which could be misinterpreted
26. Online messaging system with patient identifier could be better but are potentially expensive
27. A verbal communication can be forgotten by either person as there is no record of it
28. Conflicts may increase the number of complaints against each other in the professional bodies
29. Conflicts could end up in court
30. Nurses and doctors can lose their registrations
31. If a quick response is required in an important aspect of care, we can communicate with the senior members of the team
32. Not having a power imbalance is an important aspect of good communication
33. In practical scenario, nurses and doctors separately communicate with patient

**Timing of care**

1. Doctors do not spend a lot of time with patients,
2. Doctors come and see their patients for a brief time in inpatient setting
3. Patients do not have much more time to speak with the doctors
4. Things are delayed when nurses and doctors work on their separate teams
5. Delay in relaying information
6. Delay in review by the doctors
7. Problems with communication between doctors and nurses can leave with you pain or without pain management for significant amount of time

**Scope of care**

1. Doctors take a directed history and can understand what is happening with the patient medically
2. Doctor would not understand some elements of patient care such as soft care
3. Nursing staff can access mood, social situation, and insights and understanding to these situations which doctors do not read
4. Nurses can read the note and take actions according to the order or advice of a doctor
5. Nurses should verify doctor’s order
6. Nurses need to do more things within the scope of practice
7. Need to evolve role and responsibilities of the nurses
8. Experienced doctors rely on the recommendation made by the experienced nurses
9. Understanding the nutritional needs of the patients. Nurses make sure these things are placed into their care plan
10. People may be confident talking to people who have leadership qualities rather than those who have just started their practice

**Characteristics of communication**

1. Having an open communication that is honest is important
2. Open communication between nurses and doctors
3. Communication needs to be direct (face-to-face, over the phone, or writing a document)
4. If we do not come to a point that whatever we are doing is right, we are not achieving 100% care of the patient
5. Nurses and doctors should be willing listen to the other person
6. Communication should be honest
7. Doctors and nurses should trust on each other’s knowledge, abilities and expectations
8. Nurses and doctors need to understand that they come from different level of expertise and everybody has something to offer
9. Doctors should respect the nurses in the same way that the nurses respect them
10. Nurses and doctors need to be polite
11. It is important to have a respectful communication among team members
12. Communication needs to be professional
13. They should remain professional and polite when one is proposing something
14. We need to deal each patient as a person not as like a case
15. We need to look not at the disease but look at the person
16. (There should be) Knowing that the environment you would not be prosecuted, troubled or victimised if you complained or noticed a problem
17. Environment is not going to take a bad view of a nurse speaking up will facilitate open communication
18. Doctor is responsive, listen respectively, and either chooses not to undertake the suggestion and gives the rationale why or chooses to listen to the suggestion makes the environment conducive to open communication
19. Communicating a plan to a nursing staff and if they do not agree to it, listening to the reasons and deciding thing acceptable to both parties is the only way to progress it
20. Spontaneous communication with nursing staff that does not involve patient care will make communication much more natural
21. nurses should advocate on behalf of the patient to discuss their concerns with doctors or other health professionals
22. Both doctors and nurses are eligible to express their concerns as they are health professionals
23. Doctors need to have a friendly relationship with nurses (like a family)
24. Doctors need to encourage nurses when they are doing job nicely
25. A patient will not receive the best treatment if there is miscommunication between the nurse and the doctor
26. We cannot give the right treatment if we are not communicating
27. Communication should be timely and thorough
28. Communication should be uniform during all shifts (less in night shift)
29. A clear overview of a plan, not just a list of tasks is effective for patient
30. Lack of communication have a legal implication
31. Healthcare is vital than communication
32. Patient care should be our main priority
33. We need to be kind with each other
34. Trying to create a team environment rather than separating two teams is very important
35. Doctors and nurses should be careful about how they communicate in front of the patients
36. Staff need to be aware that sick people are around; should not communicate with a loud voice
37. A clear allocation of responsibilities may help establish good communication
38. Head nurse to be involved in critical decision making
39. Having nurse practitioners on the wards

**Disagreements**

1. Nurses and medical staff are not that great articulating what they want from each other
2. It there are disagreements, be direct, say that we have got a wrong foot here (acknowledge), and spend some time to repair that relationship
3. If it becomes non workable, it is important to bring in people who are more senior to then discuss the issue
4. Escalate this issue to the relevant manager to see if we could bring a resolution to workout
5. Talk with the supervisor, third person, or other colleagues who can solve the disagreement
6. We need to improve the support that we receive from out hospital, directors, or the supervisors, whoever are in the hierarchy
7. We need to look for a solution that suites everybody
8. It is important to find out if they are having a problem in their personal life that is making them difficult to get along with work.
9. We can rectify disagreement by talking about the point of disagreement (listen, explain own views, and then act about the disagreement)
10. One should respect the concern on his/her side of the problem
11. If any legal issue is involved, we talk with the indemnity insurance to solve the problem
12. If things are documented, they can be used for court of law if anything is used against them
13. If we do not come to an agreement regarding the patient care, we are not going to achieve the target care
14. If we disagree on certain point of view, we need to agree before we can achieve 100% patient care.
15. Getting an agreement is for the best interest of the patient
16. Any conflict needs to be settled down before the management is delivered to a patient
17. Some of these conflicts can end up having trouble or mismanagement of a patient
18. We need to try to find out own’s weaknesses and strengths through regular meeting
19. having disagreements will not be good for the health system
20. Delay in treatment can impact the health system
21. If a patient is not quite sick, you might not feel the need to communicate things clearly compared to a very sick patient

**Things that can happen with bad communication**

1. A breakdown in communication can lead to negative outcomes
2. Lack of open communication can cause trouble in patient care
3. Lack of communication can lead to a lot of complicated situations
4. patient may be left untreated until it is late
5. Poor patient experience
6. Lack in timely intervention can escalate the situation
7. Disagreements also have ramifications for actual procedures
8. Disagreements can slower recovery rates
9. patients may be admitted to a ward which they are not the right fit
10. a patient may have to unnecessarily stay in the emergency for a longer time
11. interdepartmental management/consultations may be delayed
12. If there is no proper communication, patients will not be assured that they are getting a proper treatment
13. The whole process can be vague and will bring unhappy patients
14. Lack of synchronization between nurses and doctors does not bring a good outcome
15. there will be confusion about what will be the next step in management
16. We lose your interest in the work if we do not feel comfortable working with a particular staff
17. Things are missed with poor communication which can lead to poor health outcomes
18. if there is no effective communication, patients may be missed out in the priority list
19. It can lead to worse health care outcomes related to their medical conditions on a longer term
20. patients will not get proper treatment if there is a breakdown in communication
21. test results will not be reviewed if there is lack of communication
22. patients not being shipped to the wards from emergency if there is lack of communication
23. If the nursing staff do not communicate that a patient has arrived, they can sit in the wards for hours from emergency department
24. People can have open fights on the wards
25. The number of junior staff can also impact communication as they are new to their responsibilities and they often do not know the best time and best ways to communicate to each other
26. Lack of trust may lead to undermine the teamwork or professional relationship
27. If the handover plans or concerns are not carried out, one might feel that they cannot trust the other party
28. Lack of trust may place an undue burden of responsibilities on one other to carry out the roles of the other party
29. lack of communication prevents a comprehensive holistic patient care
30. Patient is not confident of their management when doctors and nurses are conflicting with each other
31. Doctors and nurses may suffer from mental health conditions after having observed regular serious patient deterioration
32. Not having a good communication creates hostility in the workplace
33. Frequent disagreements on a long run can be mentally draining and stressful to the staff
34. Prolong inpatient stay may affect other people who need hospital beds
35. Prolonged hospital stay will increase cost to the Medicare system
36. Lack of communication can create confusion and distrust on health professionals
37. patients will lose faith/trust with practitioners or health system
38. Patient can withdraw from coming back to the hospital
39. Lack of good working environment can probably increase the turnover
40. With disagreements, nurses and doctors will not enjoy their work
41. People might not be happy to come to work because of having tension with staff
42. The biggest worry is when the care of a patient is compromised due to the tension between doctor and nurse
43. As a result of disagreements, nurses may not act upon the plans and requests made by the doctors
44. It can have a bad repercussion to patient care if nurse or doctor do not agree on something proposed by the either one
45. The work environment can become toxic and impact patient care
46. Frequent arguments can impact their (nurses and doctors) career in the long term
47. The way people inform the things can impact another person
48. If nurses and doctors cannot work together, it questions their professionalism
49. Expected care may not be achieved
50. It can result in significant illness and injuries if there is no proper communication
51. Wounds can get infected if not dressing is not done properly
52. not having communication will compromise a patient’s care
53. Due to frequent miscommunication, patients will consequently receive a poorer care
54. communication between the entire healthcare team may go wrong
55. We do not feel happy with the job
56. On a long run, we will not want to come to work
57. breakdown in communication can affect the whole team
58. Nurses mostly hear the care plan from the patients as they are not having effective communication with the doctors
59. Adverse events can occur if the disagreements are time dependent
60. Timely notification of adverse situation
61. Nurses can help prevent the side effects on the patients – DVT
62. It is good to have opinion because the other person can give right solution which is not coming in our mind
63. Involvement of family is very important when patients cannot advocate for themselves
64. It is important for nurses and doctors to ask patients what they want
65. We need to compromise a few things to make patients happy
66. Providing a space and safe environment for the patients to make them speak up
67. It may be sometimes difficult to explain what a patient wants
68. Sometimes we need to accept what a patient wants by keeping aside our belief and values
69. Patients are not good educated to seek for the health advice

**Role of good communication**

1. Good communication saves time; nobody should wait longer
2. A good communication will help reduce gap between doctors and nurses
3. Communication with doctors can give autonomy for the nurses to care the patient
4. Very young and older patients tend to deteriorate very quickly, we cannot wait very long for the doctors to see them
5. Should understand urgency of the situation
6. Nurses can help communicating with the patient while a doctor is doing surgery which makes a patient feels comfortable
7. If doctors have a good communication and relationship with nurse, it all makes it better
8. With good communication, doctors and nurses will feel more supported to one another
9. A good communication allows gaps to be filled
10. we work together nicely – it becomes easier and simple to achieve goals for patient management
11. A nice communication gives a good initiative to work
12. A good communication will help to deliver whatever we intend to do
13. Collaboration is an important part of ensuring that we deliver good safe patient care
14. Timely referral can help free up the patient beds

**Patient’s perceived care**

1. There was a lot of waiting on the ward
2. Patients want to be kept in the information loop all the time
3. patients expect respectful professional care
4. A critical component to patient care is that nurses are more attentive to patients’ needs and give or spend more time with individual patients.
5. They should act in a professional manner in front of patients
6. Patient obliged to understand communication between nurses and doctors
7. Nurses and doctors should discuss with the patient about the available options for management (dialysis, antibiotics)
8. Patients will be aware of their plan with clear communication
9. patients will not receive optimum care with disagreements
10. Patients can be exposed to hospital acquired infections
11. Patients can lose their autonomy
12. Patients can feel less empowered of themselves
13. Patients can have multitude of repercussions
14. Patients can have trauma of hospital stay
15. Patients can develop disabilities
16. What happens to a patient in the hospital can change the trajectory of their life
17. If communication is not timely, people might lose interest on their health issues (example, diabetic educator to a patient with newly diagnosed diabetes)
18. Doctors and nurses should discuss about patient’s special needs – asthma check, diabetic check
19. There could be policies to empower nurses to have better patient engagement
20. Utilizing nursing workforce in health avenues could enhance patient care
21. Nurses and doctors should be willing to discuss patient’s needs or feelings with both parties
22. Patients want to be listened to with my wishes/arguments
23. A respectful communication helps patient feel that they are being looked after by a cohesive team.
24. Coherent flow of information from the health professionals gives a positive vibe to a patient
25. The way they act, behave, communicate in front of patient can in fact, negatively impact patient
26. Patients have their most of the interactions with nurses
27. It is important to make people feel safe and cared for
28. Being around a team with consistency in the way they approach the care of the patient could make the patient feel secure.
29. If a patient perceives that they are not comfortable or there is a double standard in the management, it is going to be devastating
30. They might not feel confident, comfortable, or relaxed if they do not observe communication
31. Patient may not feel confident of your care as they can see the disharmony
32. Patients will feel the brunt of that broken team structure or communication
33. Patients are the beneficiary of a good teamwork between doctors and nurses
34. Patient is the person who will suffer for any poor communication between nurses and doctors
35. Patients do not want doctors and nurses talk about themselves in a way that it can be listened by other persons in the hospital ward
36. If you are not comfortable with the care you are getting you are not giving the good reviews about the hospital or care services
37. People may share negative views about the experience with other people
38. More people will have negative experience on doctors, nurses, or specific hospital
39. More negative feedback makes rating of the hospital becomes low
40. The patient will be extremely concerned of why these people are conflicting
41. Patient will feel unhappy if there are unresolved conflicts
42. It is not good to have a conflict with a person who is looking after (you)
43. Head budding between healthcare staff is not always in the best interest of the patient
44. patient may get disturbed with unclear information
45. People in the emergency room are probably having the worst day of their life
46. First thing a sick patient wants to be comfortable
47. the biggest thing is doctors and nurses need to be honest with you
48. Patients get involved in their care if nurses and doctors do their assessments together
49. The doctors and nurses should always try to put themselves on the shoes or position of a patient
50. A patient can easily sense or know if the doctors and nurses are having conflicts
51. If patients have a trust, they can approach to the doctors and nurses easily if they have any queries or questions
52. patients should feel comfortable dealing with doctors and nurses
53. patients expect them to be honest about everything related to their health
54. Sorting out a way to communicate so that a patient could ask them if they were not sure of anything is nice, little extra thing when you are at home
55. With making the patient understand what they are going through can improve greatly
56. You feel anxious and worried when you get conflicting messages from the different health professionals
57. We can feel quite scared
58. Doctors and nurses can improve patient’s happiness and reduce stress by providing good customer service
59. A good customer service is important
60. information may not be passed out if doctors cannot find out nurses
61. Have interest on your patient
62. Staff knowing your name – comforts patient patients are not a number
63. Assistance (interpreter/translator) if patient does not understand the doctors
64. Often nurses pick up the need of translators through their continuous interactions with the patient
65. Language and ascent should be clear to the patient
66. Doctors and nurses should speak in a simple language that patient can understand and ask questions if they have
67. We need to give people chance to ask questions and make sure that they have understood
68. Encouraging the patient to ask questions at any time
69. Patients’ perceived rejection may lead to self-harm or attempted suicide
70. Patients may become aggressive as they do not want to stay in the ward
71. There is difficulty in communicating with foreign nurses because it is hard to pick up the accent
72. New Australians or non-English speaking patients should get a clear understanding of their condition before going back to home
73. Doctor should give attention to what nurses are saying as nurses are there with patient most of the times
74. Doctors should console the patient
75. Any conversation that might relax the patient and creates good atmosphere to the patient could be helpful
76. Having a good relationship with doctors and nurses makes more comfortable
77. Surgeon should look after the patient after the operation to see the outcome of the intervention
78. Nurses should humbly ask patients about their condition as every patient are not able to tell their condition
79. Nurses should relay the doctor’s voice to the patients
80. Nurses and doctors need to be humble with patients and each other
81. Communication needs to be clear to themselves and patients
82. communication help doctors to understand methods to handle the situation of a patient
83. Patient alone in the hospital ward as relatives are not allowed except for a few specific times during COVID
84. Patient is lonely, vulnerable and does not have any support around me in the hospital
85. A patient does not want to be dismissed when he/she is vulnerable
86. Patient willing to talk with doctors to know facts on their health
87. Patients could be clear if doctors communicate directly with them
88. Regular visits (interactions) with patients
89. Doctor would come early in the morning and then the rest of the care was left up to the nurses
90. Nurses and doctors should provide sufficient time
91. Patient would be reassured if we know that doctors and nurses are on the same page
92. If you lack the confidence in the medical team that is looking after you, then of course, you will not experience a full benefit
93. Lack of communication can aggravate their physical condition as they are not able to trust medical staff
94. A good communication can help patients to develop self-confidence
95. A negative communication shuts down all the options that may be available for the patients.
96. The confidence in the medical care that you are getting is critical to recovery
97. If the nurses need to chase the doctor to have things done, it takes off confidence from the patient
98. When patients are caught at the middle of the battle between nurses and doctors, they will lose confidence on treatment received
99. patients may decide not to do things that are beneficial to themselves
100. patients may go home with issues which should have been addressed during their hospital admission
101. Doctors should be sentimental of patient’s condition (they were laughing and joking about something to be removed from my body for which I felt angry and humiliated)
102. Clear instructions and expectations explained to the patient why we are undertaking any activities
103. Effective explanation of expected outcomes would contribute to good communication
104. An injured patient at the end of the bed wants the health care team that is supporting each other
105. If the instructions are not clear it might affect on recovery stage
106. When someone in the family has a bad hospital experience, it creates a lot of anxiety among the family members even though if you are not there in the hospital
107. Consumer perspectives should be understood
108. Patients do not have access to the same information that nurses, and doctors have.
109. Patients expect communication to be in writing, so that they can access it whenever they want
110. Clear information about the after care needs to be passed to the regular doctor
111. If patients get a nice welcome at the ward, they get 50% better
112. Patients come to the hospital with sickness.
113. A friendly behaviour can make patient feel comfortable
114. We need to be vigilant about what a patient is saying
115. Patients were crying during COVID times as they did not had visitors.
116. patients feel better when a nurse communicated them with their family over phone
117. A good communication can alleviate the concerns from early in the pace
118. A good communication can soften the issue that they have to follow long term
119. if nurses and doctors do not communicate clearly to the patient, patient may not be familiar with their home care plan
120. A clear communication can settle the nerves of the patient
121. It gives patient a clear indication of their own health requirements from early on in their hospital admission
122. It is good to have a solid plan in place on how we are communicating with the patients
123. Plan needs to be communicated properly before it goes to the patient
124. Preparing patient discharge is easier when there is a clear plan
125. Patient can leave the hospital
126. Patient may not feel comfortable in the hospital setting anymore
127. With bad communication, the priority is taken away from the patient care
128. Having a clear communication will help doctors and nurses to understand the needs of the patient
129. some patients may have additional disabilities which we need to consider
130. We need to tailor our information to the level that patient understands
131. With a good, tailored information patient will feel that they are able to decide with management
132. We need to give plenty of opportunities for them to ask questions
133. It is important to use other forms of communication – visual, oral, pictorial, or video (you tube) etc so that they get that information to make an informed decision
134. We cannot assume that every patient knows and understands what we are talking about
135. Even highly educated people can have a poor health literacy
136. wrong decisions could be made based on those wrong information/report
137. There may be greater risk of hospital acquired infections and problems, pressure ulcers or infections
138. Young people may be stressed for their family if they have children at home
139. **Common barriers**
140. In general, nurses and doctors do not tend to be friends with each other outside of the workplace
141. Friendships with nurses, by no means is common as friendship with doctors.
142. It would be nice, if everyone would be on the same footy and likely to be friends outside of work, whether they would be nurses or doctors.
143. Some doctors and nurses are bad communicators
144. Colleagues do not follow advice or recommendations
145. Care can be delayed if nurses feel intimidated to ask the questions
146. Nurse and the doctor need to be able to ask questions to each other
147. It is a right of a nurse to ask the question to advocate for the safety of the patient
148. If the communication is not cordial, people might step back of asking the question or communicating things related to the patient
149. People brought up in a system where they do not ask any question to a doctor can be a barrier
150. Nurses and doctors need to be confident of what one another are doing
151. A junior doctor may feel challenging to escalate the concerns about working relationship with the nursing staff
152. It can be challenging for the nursing staff to get to know people with high turnover
153. A high patient load of the medical staff can account for some of the delays in communication
154. Doctors usually have time pressure as they need to look after many patients
155. nurses may not understand medical jargons of doctors may impact communication
156. Do not show your adversity on other
157. Understanding the underlying reason of the care (example, family abuse)
158. People might tell different stories to different health professionals, so everyone needs to be there at the same time.
159. There needs to be a space where people can have privacy so that other patients will not be able to hear your story.
160. There was no place anywhere where these confidential conversations could be had very easily
161. different people visit at different occasions to talk but not at the same time
162. coordination between the different services a patient receives should be in the same room at the same time
163. A patient does not have to repeat the same story if health professionals visit together
164. Passing on the notes and information so that the patient does not need to repeat over and over
165. communicating between ourselves helps us not to ask the same question to the patient repeatedly
166. Doctors and nurses should not tell patient to stop asking
167. Time of day can influence communication
168. Time is the biggest factor influencing communication
169. Nurses may not have time to read all the notes at all the times
170. The culture of a workplace influences communication
171. People are more receptive to things at the beginning of their shift than towards the end of their shift
172. Personal disputes at home may be reflected during work which may hamper the communication
173. Relationship between doctors and nurses can affect communication
174. There should not be a class different between nurses and doctors.
175. Doctors should not think that they are better than a nurse.
176. Some of the stuffs are best managed by the nurses for example, wound management They need to have good relationships to make people comfortable
177. Nurses and doctors should develop a sort of personal relationship with interest
178. We need to pull ourselves to a side (go aside) and discuss what is bothering us
179. Coming to a mutual agreement on which is the best way for us to work together and better
180. Gender may play an important role in communication due to masochism in place in many hospital wards
181. Doctors need to change their attitude to comment or contribution from nursing staff
182. Older school doctors can be a problem
183. Working with arrogant doctors, we need to find out a way to combat arrogance, pragmatism
184. If doctors do not agree with what nurses are telling, there is animosity which can go into a battle between nurses and doctors
185. Level of training can influence communication

**Communication tool**

1. Different ways we can use communication to assist in the wellbeing of the patient
2. That was probably a good communication tool for the doctor as well as because the nurses are changing, and he can see the names of the nurses
3. Communication boards with information on staff delegation helps to identify and talk with the person responsible for the patient care
4. Use of name badge in the hospital makes a difference; it helps nurses and doctor to call other people by their name
5. judicious use of electronic records is required
6. There are programs that turns verbal words into writings
7. There are tools made to enhance communication between nurses and doctors
8. Use of technology (Bluetooth, earbuds), hand signs or various equipment can facilitate communications
9. There can be mismatch of communication due to lack of adequate technology
10. Communicating over the phone is difficult when the doctor does not have time to hear all
11. Having a system that allows for quick communication with minimal fuss can improve communication
12. There could be some rating systems – for nurses’ notes – low priority, medium priority, or high priority that the doctor must read, and they could tick the mark on like the triage systems
13. We need to prioritize and say which one is the most important
14. Half of the information will be missed with improper handover
15. SBAR handover is good. But sometimes it is good to put the request at first
16. Methods for handover is encouraged and supported
17. Using patient name and bed number while talking with doctors could improve communication
18. There should be proper identifiers whenever talking about a patient, the more the better
19. pagers and phones can help us in conveying messages
20. For non-urgent situations that can be reviewed later, sending email or fax is the usual mode of communication
21. Developing universal system so that doctors can flag their important concerns for the patient will help to provide better care for the patient
22. Having a system that helps important things to pop up in the electronic records system so that they are not missed out
23. Using a structured communication is very helpful because we understand what is being communicated
24. Having a way to monitor if the use of technology is efficient for communication is important
25. Adhering to the existing policies if any disagreements occur
26. A simple way such as, pop-up alert at the doctor’s screen, could be set up at every facility

**Improving communication**

1. We (doctors, nurses, and other staff) should have a meeting, once in a month to discuss how to improve communication.
2. Formal regular meeting between doctors and nurses to establish common understanding and professional relationship
3. The health authority should promote communication
4. Doctors and nurses need to have both academic and practical education
5. Upskilling training help nurses to develop confidence on their care
6. We need to make sure that we are using the culturally appropriate communication
7. We need to handle the situation mutually without hampering the patient
8. A practice manager is there to negotiate between the two parties if there is no good relationship between the doctors and nurses
9. We should pass on the problems that are recurring based on the institution’s policy
10. Negotiation with the people having trouble communicating is the first step
11. Talking openly about the situation is very important
12. (Doctor needs to) orientating the nurse, who is working with you, about how you like things to be done
13. Doctors need to mould nurse to what your preferences are. So, they know you and how you work
14. Trying to communicate directly sometimes can worsen the situation
15. Having nice communication in a friendly environment will help on a long run
16. Having a meeting with a nurse is always helpful
17. With good communication between the doctor and nurse, any patient complaints can be dealt easily and openly
18. Nurses and doctors can help each other managing and solving any complications
19. if there is a breakdown in communication, we work through swiss cheese model to build those layers to prevent them from occurring
20. Having the ward rounds with the nursing in-charge with you can help
21. Take time to explain what you are thinking
22. We should not assume that everyone has the same understanding of what is documented
23. It is important to self-reflect and see if there is anything wrong you have done
24. When we apologize for the mistake, the relationships are even better than neutral
25. Any misunderstanding needs to be cleared up, or sorted out so that all are on the same page
26. Having a lovely, genuine friendship with the colleagues (nursing staff) at workplace
27. Having a good relationship with nurses has a happy outcome
28. They should spend some time together outside of this working relationship
29. Medical staff need to understand what makes nursing care challenging
30. A small change that you make can make a difference in the efficiency of care that is delivered
31. Nursing staff should be aware of the available team structure to escalate the concerns if they do not get a satisfactory response
32. Senior team members may provide guidance on resolving the issue and improve working relationship
33. Understanding the channels of escalation with the seniors of the team
34. Escalating the concerns properly to each other is important
35. Nursing staff might feel comfortable approaching a different member of the team highlighting the difficulties in communication with a particular team member
36. We need to look as a team to address the personality clash
37. Identifying the root cause of the conflict is important
38. Doctors should ensure that the nurses properly understand the plan and ask if they have any queries or questions
39. Delay in relaying information
40. Asking directly giving an opportunity for an open discussion
41. Any personality clash should be recognized as a personality of an individual
42. there should be a specific, dedicated line of communication with the patient
43. Taking ten minutes at the end of the round to talk with the nurse in charge to give an overview of what the plan is for the patients makes things easier
44. Having a dedicated time between the doctors and nurses is very important
45. Evening handover between the nurse unit manager and medical team is a dedicated time to discuss and is very useful
46. Concerns raised that are based on evidence or on their experience needs to be heard
47. Negotiation should be based on evidence-based practice
48. Negotiation during resuscitation or acutely deteriorating patient is harder
49. There should be a planned review with medical and nursing team
50. The healthcare team needs to meet regularly and include all staff
51. nurses should feel free to speak to the doctors
52. we should resolve the conflict between ourselves
53. Conflicts can be managed through direct face-to-face discussion
54. There must be a protocol to resolve the conflicts
55. we need to make sure that we are here to practice safety standards
56. Being more mindful and focused on what we are doing
57. there should be trustworthy relationship between nurses and doctors
58. Doctor’s order should be clear, legible, and self-explanatory
59. We need to improve ourselves to improve collaboration
60. Different people need to lead the communication at different times
61. everybody should have their say and be respected no matter where they are in their education or ranks
62. Clear documentation of care plan at appropriate place so that nurse can easily read it and carry out things even when doctors are not available for explanation
63. Making sure the notes are eligible and written
64. Transparent communication on decision making of the patient
65. Orientation (breaking in) of new staff at the time of boarding can help improve communication
66. Changing way of doing things can make things easier and better
67. It is important to be kind towards each other and with the patient
68. Having empathy over each other’s job will make a big difference in patient care
69. patients expect doctors to show empathy
70. A lot of the work that doctors do are not physical but have mental pressure
71. We should realize factors that make each other’s job hard
72. Professional development workshops can improve interactions between nursing staff and doctors
73. Hospital can organize training on how to manage effective communication
74. Nurses and doctors should have training on how to communicate in specific situations –aboriginal communities
75. A good mix of nurse-doctor education or simulation can be better
76. We have better relationships when we do things collectively together on teams which will help to get know to each other
77. Disagreements should be solved away from the patients
78. Any significant issue that is highlighted by the nursing staff, sensitive in nature, should be addressed away from the bedside
79. If conflicts are taken personally, it would affect the whole profession
80. Fix disagreements in a professional manner
81. Should clear doubts in an empathetic way
82. We need to make sure that it is not a hierarchical communication with people feeling disempowered with the communication
83. Hierarchy and ego should not prevent the information being available so that patient can have the best care
84. Feeling of doctor is up there and nurses are subordinate can hamper communication
85. Some of the nurses might come from the culture where doctors are ranked very higher in the importance level than nurses and nurses do not have a voice.
86. Attitude and communication skills of a doctor is key (important) as there is a power difference between doctors and nurses
87. There needs to be opportunities to direct face to face communication between various staff
88. Having a suitable space to talk about something which is very personal like suicide attempts, like being homeless, is important
89. As nurses and doctors work in rotations, what patient would hope is that there is mechanism in the ward routine for that communication to take place
90. If nurses know something is wrong, there needs to be a potential avenue where they can escalate their concerns
91. Staff members should understand each other’s thought process, and behaviour helps to achieve best outcome for the patient
92. They can hate each other outside the border of the hospital, but at the bedside they need to show unison respect to each other
93. If we notice that something is wrong, we need to follow the protocol for reporting it
94. VHIMS (Victorian Hospital Incident Management System) should be initiated so that any incident goes into the hospital system in a formal way
95. Proper documentation of communication is important as it can be an evidence

**Long term care/impact**

1. There should be good communication link between the hospital and structures within the community
2. We need to have a good preparation on sending the patient back from the hospital to the community
3. Discussion with nurses helps preparing a care plan for a patient with chronic disorder
4. Patients sit down with the nurse to discuss a care plan
5. If nurse understand the plan, they can translate the plan with the patient
6. If we do not take preventive measures, we are not controlling the chronic condition.
7. If doctors can communicate nurses for smart goal settings, nurse can help patients to achieve those goal plans.
8. Nurses play an important role in preventive measurements/medicine more than a GP.
9. we need to work as a team to provide the highest quality care
10. Wellbeing of doctors and nurses is a big question
11. Lack of communication may affect the reputation of the institution
12. Good communication will improve people’s trust towards medicine
13. If there is good communication, people will recommend the hospital
14. If there is no good treatment due to ineffective communication patient would not recommend the hospital
15. if there is animosity between nurses and doctors, patients will lose trust on the healthcare team and the hospital
16. Good communication increases the reputation of the hospital
17. If we give more pain to the patients, they will not want to come back to the hospital when they are sick next time

**Holistic care**

1. Nurse can have a big help to lifestyle modification, psychosocial impact, general health care, social home environment improvement of the patient
2. Healthcare is a teamwork
3. Effective communication benefits the patient, hospital, and everybody
4. Nurses can help doctors by encouraging and convincing patient to modify lifestyle measures
5. The difference in the medical knowledge of different level of nurses plays an important part in patient care
6. Doing things together help to hear voice of each other to understand what is happening with the patient
7. Involving multiple partners (allied health professionals, social support workers, counsellors) in patient care can help
8. A holistic approach means that other professionals like dieticians, physiotherapists, occupational therapists are also communicating effectively with the medical and the nursing team
9. We should include patient and family members in the treatment
10. Patient’s family members also feel well supported in their care
11. We need to ask patients on what they want in their spiritual and social care
12. A welcome pack or some sort of information may make them comfortable and gives a good impact
13. They gave me a sheet of information about what they are going to do
14. Stress levels can influence communication
15. If people are coming to work with personal stress, even the mood of different staff can impact communication
16. Spiritual factors also could influence communication
17. Effective communication could mean that there is less confusion about the patient care
18. An ideal patient care is provided when a patient feels that they have been looked after, not only by the best possible medicine, but also with the best possible behaviour
19. Making the patient feel that they are the centre of the care is one of the biggest things
20. Patient needs to be the centre of attention when they are having conversation in front of the patient
21. Go above and beyond to make sure to make patient something special
22. A good communication addresses the needs of the patient - physio
23. Nurses and doctors need to understand that holistic care is important
24. good communication can improve hospital performance
25. We have to make the journey of a patient easier by communicating with them
26. We need to share our plans as patients are anxious to know it
27. Communication is a balance between a patient and the healthcare team

**Challenges**

1. A person/patient with different origin may not understand all the colloquialisms of the native English-speaking person
2. Communication can be quite challenging when English is the second language
3. Lack of understanding due to accent can damage communication. Nurses cannot provide accurate information to the doctor and the doctor thinks they cannot rely on the nurse.
4. Even though the English level required for migrant workers is high but due to difference in accent, we encounter racism directed towards our clients
5. Miscommunication due to ethnic differences can be avoidable if we give sometime to understand the doctor or the nurse
6. We do not need to judge the background of the doctor. It is important to understand the information that is being provided by the doctor
7. There is a huge migrant flow in medical and nursing profession. Doctors and nurses come from the different parts of the world with different cultures in their system.
8. Junior staff often do not understand the importance of communication in escalating concerns and identifying risk with patients
9. Personality traits of the medical or nursing staff can have a massive influence in communication
10. Among other forms of discrimination, racism plays a role in these environment
11. Sometimes, doctor may not communicate with a nurse due to racism
12. Bullying in the workplace is an important issue that we face
13. Nurses bullying doctors and doctors bullying nurses is a huge issue
14. There may be significant bullying from doctors over nurses, particularly when they are escalating things
15. Foreign nurses can sacrifice bullying to be able to build a life for their family in Australia.
16. Bullying is a huge issue from medical staff directed at nurses.
17. With bullying and not having good relationships, it is often difficult for having a good reference which can hold their career for a while
18. Bullying can increase the risk of suffering from burnout, anxiety, depression
19. Bullying can also increase the risk of suicide
20. People get upset if a person bully at you
21. Subtle imbalance of communication between nurses and doctors can create master-slave relationship
22. We cannot concentrate on our work
23. It takes a long time to get back that confidence
24. Nurses need to have a time off to relax
25. Nurses can leave the job because they cannot do it or keep making mistakes
26. Hierarchical culture where nurses do not challenge the doctors prevents effective communication
27. Distinction between the nursing and the medical staff sometimes does play an important role in communication
28. With unequal relationship, one is always talking and the other never steps up to talk
29. Fostering the culture of unequal relationship will take time
30. Nurses may have more anxiety on issues with communication because of the power struggle between nurses and doctors
31. Many nursing staff are naturally intimidated by the medical staff
32. Nurse should not be bossy to the doctor
33. Nurse needs to be very kind to the student doctor
34. We need to be harsh to improve communication
35. If anyone miscommunicates, setting them aside could deliver a message that we need better communication and miscommunication is not tolerated
36. it makes hard to set the right message when the senior management team cannot communicate with each other well
37. Not to judge based on different backgrounds and religion
38. There will be reduced satisfaction that comes out of the relationship
39. There will be reduction in the motivation to do things

**Patient outcomes**

1. There are a huge range of minor complications, delayed treatment, delayed results, patient’s spending more time in hospital, they can block a big spectrum to missing things
2. We may not be able to provide adequate treatment to conditions we haven’t discovered
3. Mortality rates may increase
4. Patient can potentially even die if certain procedures that should be done are not done due to disagreements
5. Even though they may not die, there could be lifelong repercussions, lifelong medical problems
6. Patients feel less empowered to speak about or ask questions if they see amicable communication between nurses and doctors
7. Patients can be discharged even when they are not ready to be discharged
8. Doing things together is more efficient and timelier
9. If both sides feel that they can communicate freely, things will happen more efficiently
10. The patient would feel more happy, hopeful
11. When patients are happy, they would not complain about anything
12. With good support, patients might feel like they are not away from the home
13. Professional care enhance recovery of patient
14. Patient’s mental health, psychological wellbeing could be extremely negatively impacted
15. Good communication improves patient’s mindset and mental health
16. we still are not doing our job properly if we cannot address their emotional perspectives
17. Need to ask accommodation, family, spiritual stuff, and patient’s wishes
18. A collaborative team produces good positive health outcomes
19. Potential deterioration of a patient can impact patient care depending on the skills of escalation and timeliness of the review
20. Timely delivery of care plan will lead to prompt treatment and recovery
21. With good communication, patients will get right medicine
22. Medicines delivered (provided) on time
23. Adverse outcomes – infection, death, cross-contamination
24. Discuss and agree on care plan
25. Disclose/explain reasons behind care plan
26. Home environment – love, care, and affection
27. Prolong time taken to be seen by the doctor
28. It can delay patient care if plans are not implemented, or investigations are not carried out due to poor communication
29. With clear communication tests would be done quicker
30. Patients may not receive procedures, medications that they need to receive
31. Lack of communication may result in drugs are not given
32. Lack of communication may result in some treatments not being carried out
33. There is potential for harm if poor communication or the concerns of one another are missed
34. With joint rounds, things can be timely as any concerns can be addressed right at the bedside
35. With joint rounds, concerns can be addressed quickly, and errors can be prevented
36. When nurses and doctors are together in the rounds, there will be a comprehensive understanding of what is happening with the patient
37. If nurses attend the rounds with the doctors, they will be aware of the communication that a doctor is having with you.
38. It would be reassuring if the doctors and nurses are both there when the key decisions are made
39. Every member of the healthcare team should be present while seeing the patient so that they all know patient and are speaking the same voice
40. If doctors are well communicating with the nurses, they will be able to know about the patient comprehensively
41. Patient may be given wrong medication, wrong dose
42. The physical ailments of our illness could be messed up with the mistakes are made
43. Patients can get wrong treatment
44. Lack of communication can lead to fatal medical errors and catastrophic problems
45. nurses can easily make error or lose their confidence due to lack of communication
46. When there is a lack of communication, patients may not get proper advice, education, and support
47. Patients might be less likely to take advice from the medical team if there are disagreements between healthcare team
48. If they are telling the patient same messages, same education, and the same warning signs to be careful of (nurses and doctors are providing same information), it will make patients feel secure with regard of their health.
49. There may be lack of timely interventions
50. With decreased communication, things take too long to be done
51. Consequences of not having an effective communication may lead to unwanted complications
52. Daily communication with the patients from nurses and doctors increases patient’s confidence
53. If doctors can feel confidence on the nurses, they are more responsive to us
54. if there is a clear communication about what we are doing and what they want, patients will have trust with us
55. If a patient is confident of what is in the chart is correct, he can probably relax more and help me get better faster
56. patients are unhappy about the lack of communication about technical errors
57. People are angry for not being communicated end to end about technical errors
58. Witnessing disagreements reduce patient’s trust on nurses and doctors
59. Lack of trust increases distress among patients
60. Lack of trust/distress is the number one cause for hospitals being sued
61. Having frequent disagreements affect the level of trust that we have on each other
62. Having disagreements will not be good to hospital reputation and goodwill
63. patients will not get cared if there is no good relationship
64. Having bad experience at hospital or clinic can impact for the rest of their life
65. People do not want to go back to the same hospital or the doctor if they receive bad care
66. People will have doubt on the hospital or the system
67. One of our responsibilities is to explain to the patient to align them back to the healthcare service
68. Patients might not go back to the same hospital or look for a different one where they could feel comfortable
69. Patients prefer going a hospital that provides prompt service
70. Family members like prompt assessment and treatment
71. he hospitals should have an eye on the cleaning services so that patients do not feel uncomfortable while they are in the hospital or in a ward
72. Reviews on the social media gives an active impression about the hospital culture
73. A lot of factors – clean, hygienic, latest technologies, time for appointment, time for a waiting list, management of appointments, treatment methods will influence my decision for receiving healthcare
74. Good communication can give patient comfort
75. Patient would be happy when leaving the hospital
76. With good communication, things will be flagged and noted quicker
77. With good communication, things getting deteriorated get addressed in timely manner so that we can prevent further deterioration
78. Overflow of hospital beds may increase stress to the staff
79. Imbalance between the number of medical and nursing staff can affect communication
80. Australian health workforce is understaffed; it can lead to stress and pressure
81. If we do not have enough nurses for a nurse to step in for the fifteen minutes when the doctor is there in the hospital ward, it is clearly an issue
82. delay in communication can deteriorate the patient’s health
83. Some patients may need visit to the emergency because of communication delay
84. There can be increased length of hospital stay due to deteriorations
85. Lack of proper communication may prolong a patient’s length of hospital stay
86. An extra day in a hospital means, I have another day when I do not get paid, another day for me to organize things for my pets and other things
87. Family do not have sufficient income to cover for the unplanned prolonged hospital stay
88. With good communication patients can be discharged quickly
89. With good communication there will be increased turnover of patient’s beds
90. With good communication patients may find the process of being in the hospital more enjoyable
91. Delayed response can cause more serious conditions in the patient or a decline in the patient’s condition
92. (Dissatisfied) patients can be confused about what is happening about their care
93. Depending upon the doctor, nurse, and their way of communication there can be a delay in responding and reviewing the patient
94. Things can evolve quickly with good communication
95. Communication can affect the timeliness of getting right care at the right time
96. Closed loop communication helps immediate action and timely management
97. Closed loop communication helps to identify the errors
98. With lack of communication there could be a lack of observations on the patient
99. Nurses should be serious about vital signs
100. Lack of communication may impact the quality of care received by the patient
101. Lack of communication can be frustrating to the patient
102. It overall affects the quality of medical care the society will receive
103. At the end of the day, what type of service they provide matters
104. Patients may have more presentations to hospital
105. People can come back from the hospital in worse conditions
106. Patients do not have the knowledge that will help them self-medicate
107. People may have severe symptoms and refuse to go to the hospital – they will not receive any treatment – can increase mortality
108. If the health system is down, more people will lose their jobs
109. People can become physically tired
110. People will feel emotionally tired
111. People will feel mentally exhausted
112. Extra waste of time going back and forth to the hospital
113. Its been a constant cost for me ($50 per week)
114. Trying to balance between work, family responsibility and doctor has been a nightmare
115. Increased financial strain
116. It’s been a nightmare for patients to be back and forth between doctors
117. We need to look for the social determinants of health that needs to be improved
118. There is language barrier with indigenous population

**Financial Outcomes**

1. Medical insurance overbilling
2. Socioeconomic cost associated with health care industry
3. Private sector- cost saving rather than spending
4. It can impact the budget and cost effectiveness of treatment. For example, if there is a debate around undergoing MRI and if it returns normal, there is an impact of the cost of a procedure, time, and human interaction if the tests come normal
5. In public hospitals they are investigating appropriately, but not over investigating
6. a patient’s expectations are meet in a private facility, but at the financial cost to yourself
7. It can result in increased need of investigations and specialist consultations
8. It will increase the expenses (out-of-pocket) for the patient (insurance will cover only a part of the cost associated with specialist review)
9. It is really bad if lack of communication between nurses and doctors results in complications
10. A good communication will affect the economy of the whole system – hospital or community
11. When the stay of a patient is prolonged, the government needs to pay more for the patient, there is need of additional resources
12. A prolong stay of a patient will affect the budget of the hospital
13. we will not be able to make room for the sicker patients
14. It will prevent other patients to get service from the hospital (bed occupancy - we could have admitted two patients in place of the prolong stay)
15. Deteriorating health of a patient will put burden on the healthcare system
16. There will be a drain on the patient’s ability to work

**Parking lot**

1. As a healthcare team, we need to support the way a patient wants to live their life
2. nurses and doctors need to understand the structure and role of each other
3. One party or other may feel that they are not heard, or their concerns are not escalated or appreciated
4. We all should be communicative about the treatment plan, side effects and other management
5. Lack of having a grasp people’s skill levels can affect on what is being escalated to them and their understanding of it
6. We need to make a standard, reinforceable means of communication so that everyone knows how to get what they need across
7. There should be a facility-based agreement on what to do in case of disagreements
8. Messaging through writing in the patient’s notes can be missed but is important to do it
9. Use of sign language may not be clear to the patient
10. Language barrier can be an issue
11. Language skills and different ways of communicating of nurses can influence communication
12. We need to be aware that we mean the same thing with all our language that we use
13. A surgeon was a god sitting up on the cloud
14. They should have discussions about you even when you are not there
15. They should discuss the case and what to expect before going to the client’s room
16. They should have more meetings about patient
17. They should do things in the right way
18. Sometimes nurses do enough to express what is going on negative repercussions for a patient’s care
19. Nurses go through with the doctors sometimes too fast and too quickly. It may be because they are so busy. But I think they need to slow down little bit.
20. Some of the discussions should not be done in front of the patient. It heightens their anxiety and stress levels.
21. They need to discuss anything discretely probably in an office or somewhere not in front of a patient
22. As doctors work in different hospitals, they may not be aware of the hospital protocol and policies around a certain thing whereas the nurses know that stuff
23. A lot of communication between doctors and nurses happens outside of the room, in the corridor
24. Disagreements can alleviate worry or anxiety
25. Not knowing what is happening obviously could add to anxiety
26. There was a lot of time nurses saying they were not sure what is happening.
27. There is shift work, but if same nurses are there over days, it is easier for communication
28. I could hear nursing staff talking in frustration, as they were unsure of things going on
29. They should consider as a person rather than a patient
30. doctors should not show their frustrations with nurses
31. They should have one spokesperson (to prevent everyone rushing) to address the queries of the patient and represent the whole team
32. Before seeing the patient, they should read about the case and know what is exactly going on
33. If there is no communication, it would seem like they do not know their work, they do not about the case. It would not reflect very well among either doctor or nurse
34. The attitudes and behaviour of both nurses and doctors in public and private healthcare sector is quite different
35. It will help them feel free to ask any questions or doubts regarding their operation and management
36. I would have felt clueless if there was no communication.
37. I would have got frightened by knowing that there is nobody to take care of.
38. They might not be interested on work; they might turn up late for work, take leave, inform leave, take sick leave which may affect the whole hospital.
39. Infrastructure, services, equipment, hygiene, cleanness of the hospital needs to be improved.
40. Communication should be clear enough so that the nurses can treat the patient as mentioned. If it is not clear, they might do something that is not actually needed and may affect the patient
41. It may make feel the nurses that they are not been trusted enough, they might think that they are not good enough for work. They might feel isolated at times.
42. Many personal and institutional factor could affect communication between doctors and nurses
43. doctors and nurses should think they should have the same team spirit to work together.
44. Collaborative care could be introduced through education in their schools and at the hospital through training programs
45. Proper communication will ensure ideal treatment methods and would be beneficial for the patient
46. Lot of people do not have a choice between public and private hospital
47. Some of the doctors and nurses are very unapproachable and scary
48. nurses are having more pressure if they are not having proper and correct communication
49. There are doctors who have an aggressive personality and nursing staff who may not like a doctor
50. It is not about having a bad result or a good result, but it is about having an appropriate result, the right one
51. I do not think gender of a nurse matters
52. Nurses are the one who end up making the mistakes if there is not good communication
53. It is important to have an informal conversation to establish a good rapport; patient needs to be taken care before having such conversation
54. personal discussion should not be conducted in front of the patient
55. It is dangerous if people let their personal opinions influence patient care
56. Hospital is already a stressful environment and with added pressure of working with a difficult person would add to anxiety and depression about going to work
57. If people are not going to work because of having mental stress of going to work, we will have a lot of sick leave to cover. Overall, the organization will implode
58. Miscommunication can lead to problems within departments. The department may be dysfunctional to the organization
59. When there is a rift between doctors and nurses, other people involved in patient care often feel hesitant and get confused with their role in patient care
60. Disagreements are common in a high stress setting; the way they are handled is very important in terms of how it affects patient’s perceptions and care
61. Disagreements can help pick up things that have been missed out through exchange of information between doctors and nurses
62. Disagreements that occur in front of the patient can reduce patient satisfaction in the care they are receiving
63. It will create a negative image to the organization when a patient witness disagreement
64. Repeated disagreements over a same issue does not make a good working environment for nursing and medical staff
65. Repeated disagreements reduce their productivity of the department and staff
66. Disagreement damages the perceptions of each profession has over other profession
67. Wearing masks in the healthcare setting could influence hearing and understanding
68. People can gossip about disagreements between nursing and medical staff, which can create further problems within the department
69. Performing tasks independently but converging or meeting if there are problems or issues through good communication
70. Highlighting the points of contacts between shift will allow nurses and doctors to have expectations of when they are communicating with each other
71. Setting up the expectation from each other through regular conversation
72. Approaching patient care with a unified approach is important
73. Team focused approach involving the whole team and not just doctors making the decisions
74. Nurses appreciate if at the end of the ward rounds doctor provide a summary of what is needed, or any specific changes have been made
75. If doctors are not approachable to the nurses, things on the priority list can be dropped down
76. Doctors want to know earlier if any patient needs an urgent review so that he/she can triage things
77. It helps planning early and appropriate discharge
78. A good communication helps planning out rehabilitation beds (through early referral) which are often scarcity
79. it is also not good for patient to be in the hospital as all patients cannot get their intense need (for example physio)
80. good communication helps patient to get out of the hospital system as soon as possible
81. Doctors want to know a clear picture of what is wrong with the patients
82. A good relationship with nurses makes the job of a doctor easier
83. A good relationship has a big impact on the efficiency of the workplace
84. A mucky day never makes anyone feel good
85. If there is a good team that we can get along, we always look forward to coming to the work
86. Doctors need to acknowledge the role nurses in patient care
87. Having a task list makes things more organized as we can leave the notes next to each task
88. Having a paging system or having a system for face-to-face communication for more urgent clinical things is important
89. Doctors do not have set breaks like the nursing staffs
90. Nurses identify the need of other things for the patient which are missed out by the doctors
91. Good communication helps to provide not only the physical care but also the mental care and the functional care that might be needed
92. It provides a pleasant experience to the patient if they feel the whole team has the best interest on them
93. We need to make sure that there is a good level of mutual respect between all levels of health care professionals
94. Timely care is important in our overloaded hospital systems
95. With good communication, patients will come back to the same hospital in future
96. If patients do not trust the assessment or advice by a doctor, they are not going to follow it and their health will deteriorate
97. Having a dedicated time where nurses, doctors, and other allied health staff can be physically present at the same space to talk about patient is most helpful
98. It can hurt the confidence of doctor and nurses
99. Notes, verbal (face to face), telephone or paging system
100. we can get the information promptly with face-to-face communication
101. Patients will get better care when each party listens to the other
102. Nurses can help patients to have their concerns passed on (patients can have nurses as another avenue to being listened to)
103. Sometimes patient may not want to tell things to the doctor; nurses’ observations might help
104. (Tools for) communication can remind the medical staff so that things are not forgotten
105. Nurses’ can provide the valuable suggestions about the patient care which can accelerate care pathway.
106. Patients might get deteriorate in the ward because they are not getting treatment, they are supposed to
107. valuable information can be lost if one party does not listen to other
108. If the concerns from nurses are dismissed by the doctors, they feel bad and think that can’t raise concerns in the future, which can impact a different patient
109. There is a fine line between having disagreement and conflicts
110. If it’s a fight between doctors and nurses, it is helpful for the senior staffs to get involved. Escalation to them earlier on can alleviate distress
111. When there are a lot of sick patients, everyone is stressed, and communication is worst in these cases.
112. Having a debrief session after a stressful patient encounter can be helpful. Having this dialogue between doctors and nurses can help improve things for the next patient as people can work out why things were done in a certain manner.
113. Handover can be improved so a quick synopsis of what is happening to the patient and the assessment could be relayed
114. When these things are addressed, they have a better chance of not being readmitted
115. Nurses can help provide a better plan for patients to manage their chronic illnesses once they are discharged from the hospital
116. Paging can be problematic because the pages fill up in a busy shift with requests and we need to cycle through them
117. It helps nurses and doctors to work together rather than fighting about who is going to do it or who can do it.
118. Understanding the social condition of the patient helps us to better manage the patient
119. Patient often disclose their concerns more to the nurses than to the doctors.
120. A lot of doctors’ assessment is based on what nursing staff tell them
121. Code blues or medcalls, that are run very well because of teamwork.
122. Doctors know when patients are ready for discharge from what the nursing staff tell us.
123. Children are better in their home environment. The faster we can get them discharged safely, the better
124. Communication gets difficult with locum doctors/nurses whom we have not met, as we are not sure of their clinical skills
125. We are more likely to have conflicts in wards with locum or seasonal staff, where we do not have the same understanding of the staff
126. Sitting with the nursing staff while having the lunch gives opportunity to know each other
127. The parents can put their frustrations back to the nursing staff, who can fire that back on to the doctors.
128. If we have doctors, who are there for a few weeks, it is barely enough time to get know the staff, get along to make an impression
129. Senior doctors can disagree with nursing assessments and nursing judgements. They think because they are senior, they can do that.
130. All the nonverbal communication cues as well are important
131. Any nonprofessional conduct needs to be reported appropriately.
132. It makes a big difference if a doctor calls nurses by their names in front of the patient
133. Introducing team will give confidence to the patient that we all understand each other
134. You get to know the team you are working with by getting mixed with them
135. The work would be easier if we know your team
136. Nurses help doctor to monitor the patient’s telemetry and let the doctors know
137. Clearly explaining the nurses with parameters that a doctor is concerned of and not documenting only them on papers
138. Doctors may miss some important things simply because there are so many pagers from the nurses
139. A junior doctor, will not feel respected if the nursing team is confirming the decision/plan with the other member of the medical team
140. Personal style of communication, clearing concerns can impact communication
141. Using shorthand or acronyms
142. Tailoring communication to different groups of people can help
143. Having processes in place, not in antagonistic method, punitive way, when identified when there are conflicts between staff
144. When there is workload, the idea of working in a team- communication could be set aside because they have so much of the health care related works to provide
145. having non-confrontational ways to deal with interpersonal conflicts can help
146. In the punitive version, where you get rid of the people who are causing with problems are replaced with others, loses knowledge and experience.
147. When there are certain antagonistic things between doctors and nurses, for example yelling at each other, that obviously affect the patient
148. Innovative methods of communication – trial of application where nurses put information about the patient and their observations – could help
149. LAN paging system will not allow a detail communication
150. Having a robust communication system is a key to improve communication
151. The plan of care gets more established if nurses are there with doctors during patient rounds
152. Nursing staff are good resource for the doctors to rely on to highlight the issues that might affect the timely discharge
153. Seeing senior doctors communicate with nursing staff in a professional way can reinforce professional expectations of junior staff
154. It is demeaning and demoralizing as a junior when your practice is questioned in front of the patients and their families and over time it affects the self-esteem as a junior doctor
155. A senior consultant might not appreciate the importance of keeping the nursing staff in the loop and the letting her know early about things that can affect the plan of care on the day.
156. Junior staff in the ward more easily recognize the importance of keeping nurses in the loop and be more active in finding the nurse and letting them know about the plan of the patient
157. A busy day with complicated patients can make them less receptive to hearing about more complex plans and other jobs that we have asked them for
158. Workload can impact the level of communication
159. How a person handles stress is also important
160. During disagreements, it is beneficial to have coordinated discussions between the healthcare team or perhaps a senior member involved in the patient care
161. Try not to insult or offend the person whom you are discussing with.
162. Doctors should realize that the nursing staff can be of valuable in their clinical skills
163. Not everybody has the time to read all the notes that is written in EMR; plans might be missed then it just gets all over the place
164. When nurses and doctors do not sense any urgency in the patient during their communication, then the patient tends to stay longer
165. The nurses’ thoughts of patient’s condition help doctor to manage the symptoms better and able to get through the diagnosis faster
166. When vital information is missed or not communicated with the doctors, it can lead to increase in the morbidity and mortality
167. When a person comes to the hospital, it is the job of doctors and nurses to help them get better and not to worsen their symptoms
168. It can cause more problems or harms than good when there is less communication
169. When the matter/request is urgent, keeping the key points could be helpful
170. Break time can be a factor. Doctors and nurses go on breaks. Sometimes, we need to find each other but we cannot find them as they are on breaks and we do not want to disturb either.
171. We can easily address even the tiniest concerns that they might have
172. Good communication can help to elevate the mood of the patient
173. If patients see people around them in a positive mood, it helps their immune system
174. Speaking out with a colleague or anybody lets out your frustration and helps to move on
175. Changes in plan does not get enacted due to disagreements
176. An informal communication to give an update about a patient can be helpful
177. Coming back and checking back if something has been done and making sure that someone understood you well is massive thing
178. sometimes being friendly can have detriment effects as people tend to ask more things or ask for favours with an approachable person
179. We need to flag the urgency of what we are discussing
180. We need to foster an environment that facilitates open communication
181. Body language and tone is important in communication
182. Maximising the use of available resources is important.
183. Efficiency will change massively if we can get the right person taking charge of the communication
184. Patients are the sick persons who want reassurance and wants to know if they are going to get better; conflicts can have impact on them mentally
185. If you do not feel safe and secure in your workplace, you will feel more fatigue and will lead to high level of burnouts and not lasting in jobs
186. Because of COVID, visitors are restricted, and communication is mostly dependent over phone; it mostly relies on nurses to communicate that
187. Communication at right time so that a person is not distracted while they are doing something more important
188. If nurses do not have the correct information, the hospital is not able to response appropriately. It delays in patient care
189. Introducing yourself is a big thing. More the people know your name, it improves communication
190. Errors are prevented if you are an approachable person as someone can easily double check with you
191. Nurses should be able to say to the doctors if they are not able to do or perform any tasks so that it could be delegated to some other person who would be able to carry out
192. General flow of the hospital can be impacted
193. Nurses being able to communicate things with family members is important
194. If the communication is good, the work atmosphere is much more collegian
195. Doctors always enquire a brief snapshot of what is going across the day whereas the nurses have a much large amount of time at the bedside
196. What doctors see might not be representative of how things are going more consistently
197. In psychiatry, doctors should quickly be responsive to the concerns of the nursing staff to prevent being harmed agitated or aggressive patients
198. Any health interaction is provoking anxiety for most of the patients
199. Understanding of what is going on decrease frustration
200. It makes a GP visit a lot more efficient because everyone is on the same page
201. There may be problems in escalating things to intervene which may lead to late diagnosis and more serious complications
202. if trust breaks down due to lack of communication between doctors and nurses, there could be avoidance of accessing care for the preventive healthcare from GP
203. There will be reluctance to come back even when things are not getting better with the initial management
204. They might seek out health care from another team.
205. There will be a loss of continuity in care as patient moves from one healthcare to another
206. It can contribute to a detrimental hierarchical culture where both sides may not speak and listen to each other
207. There is a lost opportunity for education between the two parties
208. There is better workplace satisfaction with good communication
209. It is an enjoyable workplace if all the people feel valued and can contribute to patient care
210. There needs to be appreciation of non-judgemental communication in workplace
211. Models of care (SIBR - Structured Interdisciplinary Bedside Rounds) with engagement of multidisciplinary team along with patients helps to improve patient care
212. Expect if the doctor has said something wrong, the nurse would pick me up and seek clarification in a graded assertive scale
213. Building relationship over time to get to know each other professionally and personally can help to build respect and understanding
214. If we focus much on the pathology or the treatment of the illness, we could miss out the other psychosocial impacts, financial aspects of the patient care
215. Training and cultural shift would help
216. A verbal miscommunication or mistake would ultimately lead to consequences and things happening which was not a part of the plan.
217. Communication sometimes can be dependent on the roasters of doctors and nurses
218. It can help to track the progress of a task so that we can prevent a day being wasted
219. doctor do not need to spend a lot of time reading the notes if nurses can communicate to the doctors at the morning ward rounds
220. Sometimes lapses in the communication leads to delay in newly charted medications
221. When the plan is not there, the nurses call for the cover team, or the medical cover, specially at the night for example, it takes time to review and assess.
222. If there is well documented plan, nurses can act on behalf of the doctor (for example make discharges in the morning)
223. Patients complain that their queries are not addressed because the doctors do not listen to the nurses or the nurses do not tell the doctors.
224. Most of the times patient withhold information themselves as they do not feel comfortable communicating with nurses and doctors
225. Lack of communication may lead to missing information that patients might have talked about
226. Patients may be agitated, anxious and do not want to engage in communication.
227. Patients will hesitate to relay their concerns, communicate with health professionals and engage in their own reviews
228. The number of years of experience could determine how a doctor might communicate to a nurse or a nurse communicates to a doctor
229. Stressful situation and inexperience of a doctor might lead to doctors being dismissive in their communication
230. Over confidence of graduate nurses may end up being a medical issue
231. More senior doctors and nurses can be rude to the juniors and may not want to listen to the other party.
232. Senior nurses may start implementing their own plans or teach the junior nurses on how to medically manage the patients which may affect patient care
233. Years of experience can positively or negatively affect the way they communicate and eventually affect the patient care
234. In some hospitals, communication between doctors and nurses may not be a priority
235. Some hospitals may encourage or advocate to have good communication and working relationship with your co-workers
236. The number of years of experience will shape things but the environment or setting of a hospital can also affect communication
237. Hierarchy is not so prominent in a rural hospital. It is not hard to ask help from a consultant.
238. With power imbalance, people will be defensive and dismissive; you do not constructively create better plan; and at the end we cannot create a definitive solution to a problem.
239. Training people, educating people to speak about different pathways of advocacy
240. Doctors acknowledge that nurses understand a patient’s needs
241. doctors view equality between team members
242. private hospitals want patients to have the best experience
243. doctor come back and see the patients before they leave the hospital
244. Empathic attitude to patients
245. patients feel uncomfortable when doctors outrage nurses
246. Patients need to be made aware of the disagreements between nurses and doctors
247. Feels like being undermined of your skills and is very frustrating
248. Often nurses explain the difficult conversations (between nurses and doctors) with the patients
249. Intervention from hospital management to improve communication might work
250. One negative incident can stand out all positive communications
251. Little things that we do make a positive experience
252. Joking with each other, singing the song over the radio can comfort patient and make them relaxed
253. Engaging a family member or carer if there is difficulty establishing communication, for example, parents involved for paediatric patients
254. Tailoring care to the needs of the patient gives them better experience
255. Television in the recovery wards, icy poles, colouring pages, stickers could help relieve stress of paediatric patients
256. Recognizing how skills from different persons can give best experience to the patient
257. Knowing how to communicate with difficult persons
258. We need to be more mindful of how we communicate and share information with patients
259. We can motivate the person continue the treatment depending on what kind of words what we use to tell them.
260. If we communicate negatively, patient will not have any motivation towards a proposed therapy (physiotherapy)
261. A great supportive, teamwork would be felt by the patients.
262. Nurses and doctors should be open to understand and step on each other’s shoes
263. They should be open minded without any personal judgements
264. A good teamwork and leadership would be win for ourselves and for the patients
265. A nurse would refuse to talk with the doctors if they are rude or would not listen to
266. Even with disagreement, we need to investigate together to check what is right for the patient
267. We need to look at the person and not at the disease itself
268. We need to learn how to set our mind focus on patient care. Knowledge, awareness, and understanding is required
269. Once we discharge a patient to home, we do not know exactly what will happen afterwards if he does not come back to us
270. Nurse-doctor communication can expediate care or improve care.
271. Doctors often work on a setting (ward) where they have no previous experience, so they very much must rely on the nurses to show them how the system works
272. Patients can receive care based on the experience of senior people with the junior staff being able to ask the questions and get the support from nursing and medical team
273. Doctors can benefit from the knowledge and experience of the nurses if they are happy to accept our support and help
274. The consultants are orientated, when they start in our department that the nursing team are the part of the whole team and need to be used as a resource and have the knowledge and information that might help them find their way.
275. If the family can see strong and helpful relationship between doctors and nursing team, it gives them the confidence to know that they are going to receive the best care.
276. Nurses provide the medical staff the information that they need to make their decisions day to day.
277. The doctor can disagree with the idea from nurses, but we can talk about that together and smash the ideas.
278. We do not have to like or hangout with everybody at work. We just need to remain professional, respectful in communication with that person.
279. If both parties are prepared to change their behaviour, you can discuss that with them and explain the effect it is having to the rest of the team.
280. It will be very helpful when you know your regular team.
281. Having to know your audience will help sometimes to get the best communication style.
282. Having gatherings to socialize with colleagues is helpful in creating good working relationship
283. At times, we do not good talking to each other but do better talking to our peers
284. understanding of how things are done changes the way we communicate
285. understanding of how things are done changes the expectations of one another
286. understanding of how things are done changes helps to be more supportive
287. The speed of information that is provided can be a barrier
288. Patients can be a barrier as they may prefer a specific person for communication.
289. Understanding and perception of patient safety or quality care can be a barrier
290. Risk perception may influence what/how we communicate can be a barrier
291. Sometimes even the most experienced people are not the best one ever.
292. Patients care a lot about how they have been treated
293. Patients care about how people communicated with them
294. Patients care about the person who took time caring them
295. Patient will not be interested in any activity if he is not feeling comfortable
296. Decision making and critical thinking involved in prioritizing and identifying the care required influences communication
297. Patients themselves play a factor in how communication occurs between doctors and nurses
298. If some patient is presenting with behaviour of concern, nurses and doctors should work together to support those behaviour
299. Setting expectations and a form or orientation when people start a new role or take a portfolio is important
300. When nurses have no idea of what is going on with a patient, they do not know what they are doing
301. Lack of communication may be due to ignorance
302. Sometimes, we can do things immediately but at other times, we might need to wait for the resources to come
303. We need to think about the resources, people, and the helping hands
304. When communication is not clear, nurses need to focus on the patient care on whatever needs to be done quickly
305. When nurses find error in documentation by a doctor, they need to communicate with the doctor and get things sorted out to prevent bad things from happening
306. Utilizing each other’s resource including those of allied health as a team promote patient outcomes
307. If there is no communication, doctors may be angry for being called up later in the night because they have not clearly documented or handed over things
308. The doctors may be angry in the morning if the nurses have not communicated specific issues overnight and the patient becomes more unwell
309. A young nurse may not have the skills to communicate the issues back to the doctor
310. When a nurse is busy, the doctors may not stop her to give a direct handover of the patient
311. Doctors can communicate long term plans with a senior nursing staff member who can handover it to the nursing team is usually effective
312. Having time away from the external stimulus to have speak one to one without interactions and distractions is helpful
313. It should be a lateral platform between the doctors and the nurses
314. The way people communicate can be a positive experience for one and negative for another
315. At times, nurses do not know the best way of communicating things with a doctor
316. When a nurse feels to be confronted, she will ask another person to help her which is not the right way to communicate
317. Nurses should understand that it may be ok for them not to receive the answer the way they wanted from the doctors.
318. Nurses should try to navigate in a different way in the situation rather than getting frustrated.
319. If we do not communicate with the patients, we cannot build rapport with them.
320. We need to communicate with the patient openly to get the information on what they are going through
321. Communicating with patient makes it easier for doctors to diagnose the patient
322. Missing out these tools in surgical wards may lead to wrong surgical intervention to the patient
323. If one person is doing one thing, it needs to be communicated with the team, so that the other members can focus on different things needed at that time (eg. Code blue – airway by one person, breathing by another and drugs by the third)
324. we need to communicate with others of what we are doing and asking them for help as we cannot do everything by ourselves
325. A good communication relieves panic inside your body
326. At instances, we cannot continue with the disagreements, we need to act on the situations and act/mould immediately
327. If we do not tell the patient and their family of what is going on, they will become tense; patient may stop taking medication
328. Doctors have more information and views on patient’s conditions
329. When a doctor responsible for the patient care is not around, the nurse must approach a different doctor; patient is more important than which doctor is responsible for the patient
330. Nurses have to read the patient; if something happens to a patient, everyone would be responsible
331. What a doctor does would reflect on the nurses and patients
332. Hospitals have taken some steps to control conflicts between doctors and nurses
333. Nurses cannot just sit down and bear everything; nurses need to speak their part
334. Doctors need to understand that nurses do not ask them to come to see a patient until they think they need to see the patient now (immediately/soon)
335. A nurse may end up knocking at the door if she thinks that worth it
336. A deficit of proper discharge plan with appropriate follow up appointment can contribute to repeated readmissions
337. Educating doctors to delegating more responsibilities to nurses
338. If patient is not communicated clearly, they may not follow the instructions given by the physician/GP
339. Difference in perceptions of what a patient can or cannot do – nurses can be more realistic, and doctors can be more idealistic
340. There can be a lack of communication between what a doctor wants to have and what a patient can afford to do (for example, a doctor wants a patient to have insulin three times a day, but the patient can afford to have it once a day)
341. With effective communication, it is easier to follow up a patient on how the plan went
342. If doctors reply to the nurses’ query, it is an easy way to transpose that information directly into the patient’s notes and can be easily passed on to the next doctor and nurse
343. It can help nurses make aware of things they are not aware of or the doctor might not have thought that way
344. If we do not get a good harmony at the bedside, patient may think that the team is not getting along with each other
345. If patients feel that you are not respecting the team, they will not respect the doctors which is not good for the quality care
346. Doctors should be self-caring to their patients
347. Our general health services is making client dependent rather than making them independent
348. Lack of the practical aspect of nursing care in the University may impact nurse-doctor communication
349. A change of way we look at things philosophy within the concept of being a nurse and doctor
350. Doctors should know their nurses or at least read their name on the batch
351. There should be a strong nurse leader who can stand up on behalf of the nurses
352. Every person should mould and be shaped into with the ward culture
353. Having clinical meetings for every patient make sure that there is a client centre based three-way communication between patients, nurses, and doctors
354. Doctors should make the patients lead on the communication could lead to create more open-ended questions/discussion
355. A 2-minute walk at the bedside with the nurse could help doctor as nurses can fill in the gaps between doctors and patients
356. We have to change the perceptions of the society that health is not a given thing
357. Society needs to take the ownership of health
358. Patients want good explanation from both sides
359. Patients want to have a clear pathway of goals or what they can expect to be in the hospital
360. Nurses and doctor should check if a patient has understood what has been told
361. Nurses and doctors should think of ways to make patients understand – print, videos
362. Doctors and nurses should look for different ways to enhance the journey of the patient
363. With disagreements, we can seek the patient’s opinion before escalating the matter
364. We ill not feel good mentally – it affects performance
365. Negative emotions can make prone to make errors
366. With negative emotions we can forget things
367. With negative emotions we are not getting the exact things done
368. We can attend counselling programs that they might have
369. Disagreements with one person can affect the whole team
370. Having enough rest between work is important
371. Patients deserve for what they have paid for
372. Grievance policy at the workplace can be utilized
373. Resolving the problems in a team will help each other to do best for the patient
374. It will not give good impression to patients if they see nurses and doctors fighting with each other
375. A good customer service can keep patients with you and hold out
376. It is very important that results are conveyed in a professional, articulate way so that the doctor knows exactly what to do
377. It is the responsibility of a senior nurse to make sure that the junior doctors are doing their job
378. If a nurse does not have courage to speak to a doctor, she could go to the senior nurse, who can speak on their behalf and make the connection
379. Hospital and staff need to learn from these mistakes in order to prevent these mistakes to happen in future
380. The managers (senior nurse/doctors) should have to look at the conflict to improve the communication
381. It can also be a very challenging to get right communication
382. Everyday the nurses and doctors might be working with different people, it adds extra challenges to communication
383. Patients may feel hospital environment is not good for them if they have delays for some reason
384. Doctors often may not think about some important aspects in women, for example breast feeding, which may impact the management
385. With an effective communication, doctors and nurses are aware of the alternative plans in emergency
386. If there is no communication, nurses will not be able to know what the red flags are
387. It may cause stress to the doctor if nurses are repeatedly calling them when they are very busy
388. discussing the situation before each case is valuable
389. it is effective to have compulsory communication
390. With so much of PPE (mask), there are physical barriers to communicate
391. use of non-secure communications about clinical situations (texting, sending pictures over phone) may make things efficient but there may be concern to privacy of the patient’s information.
392. Treatment may not include the side issues or other underlying medical conditions
393. If a patient goes home without a proper follow up or proper communication with their GP, it can lead to detrimental once they get home as something might not be covered or taken care of during the hospital
394. Having things done promptly can promote responses to treatment
395. Appreciation between the staff members – together as a team is important
396. shortage of staff makes it very difficult to make time for good communication with the doctors
397. long work hours will have a major impact on communication
398. nurses should pay attention to those issues that may have a big impact on recovery
399. Nurses and doctors should put enough details on what is passed on for others to carry on the continuity of care
400. A specific sector of the community may not be able to express themselves openly to another sector of community
401. There can be misunderstandings
402. If a patient trust on a nurse or a doctor, they are more receptive towards their expressions
403. Uneven distribution of doctors and nurses in primary care can cause delay in care
404. Keeping our knowledge to the standard (RACGP) practice or current evidence regarding the practice
405. Elderly nurses with a lot of experience snub the new nurses who are fresh and just been out of school
406. Patient feels comfortable if they feel free/comfortable to ask questions about the care provided to them
407. With a shift work for every 8 hours, information gets lost. So, we need to trust on what they are doing is right.
408. There needs to be a system, an integrated system so that whatever the nurses write on the file, the doctor needs to get it from wherever he is, even though he is not at the hospital.
409. Having one nurse allocated to two or three patients and if the same nurse covers the duty the next day so that they know the patients could make a difference.
410. Nursing stress level, their time commitments, the number of patients that they have got can influence communication
411. When there is a crisis in hospital or emergency when nurses have to participate, they might not be able to be around when the doctor comes in.
412. Lack of understanding of a medical term specially when we have a patient who is scared to ask for explanation in simple words.
413. The frequency of visits by nurses was getting less as I was recovering from my surgery. I understand they had other patients to care, but sometimes I really needed them.
414. If they had a free flowing communication channel/program that the doctor can check in even when he is not in the hospital on that would be helpful.
415. Good advice from the nurse
416. I was not given any information till the next afternoon. I was not sure what would happen to me. A little bit more of communication would have been better
417. I was put into different wards and rooms because I could not find space; the doctors and nurses who were caring for me had no ideas of what was happening to me. They were confused and waiting for the information
418. Compassion from the nurse
419. Have a second opinion
420. Debriefs to seek point of view
421. Support nurses and doctors at the same time without compromising the care of the patient
422. Nurses more responsible on care side
423. Lack of motivation among nurses
424. May underperform in the job
425. Doctors are not there in the facilities in the remote areas
426. Communication depends upon which setting you are in
427. New doctors – interns, registers do not learn how to communicate
428. There can be a gap in the knowledge if we are talking to people with different level of education (example – RN talking to a consultant)
429. Nurses need to learn quickly on how to communicate with each other as they spend a lot of time in communicating with things changing over time
430. In rural areas, we need to talk to the doctors over phone, and sometimes if lucky, over video conference
431. Communication depends on the level of education that we had on communication and on the level of your experience in the job
432. In the rural areas, nurses expect to work the whole patient up, expected to know exactly what a doctor wants to know over telephone
433. In rural areas, nurses do not use SBAR or any standard form of handover that frustrates our teams; because, then the nurse may not tell the doctor what she needed.
434. Nurses may not identify the clear issues because that might come down to necessarily not knowing what was wrong with the patient or of poor assessment
435. Sometimes it is difficult for us to interpret and figure out what is wrong with patients
436. When there is disengagement with the patient, the right to access the quality healthcare is in jeopardy
437. When there is no respect between the clinicians, patients feel that their healthcare is in jeopardy
438. It will make feel the organization or facility that there is potentially wrong with the service
439. Ongoing disagreement create an ongoing disparity in health which may not be limited being local, but can be state level and global perspectives
440. Being angry at job when we do not feel that sense of value
441. A nurse calls doctor mostly for problem solving
442. Feeling distrust with the clinicians is due to the difference in education and knowledge
443. Even with disagreements, it is ok if you stay professional
444. Disagreements may occur because people do not realize that they are communicating badly
445. When nurses communicate with the doctors, they need to make sure that the service is provided to the patient
446. People need to communicate better to overcome barrier due to knowledge gap
447. We need to trust on somebody else’s assessment. The point is do you trust that assessment; does that sound right; otherwise, I need to do it myself
448. Patients from rural community do not want fancy hospital beds in the city; they want to be sitting with their family or live with their poorer socio-economic conditions
449. People need to learn communicating in teams
450. People need to prioritize art of communication in their education in all the streams
