# Supplementary material for: Stakeholders’ Perceptions of How Nurse–Doctor Communication Impacts Patient Care: A Concept Mapping Study
Source: Nurs Rep. 2023 Nov 6;13(4):1607–23. doi: 10.3390/nursrep13040133 (PMC10661264; doi:10.3390/nursrep13040133)
Supplement: Supplementary file 1 [file nursrep-13-00133-s001.zip › nursrep-2581629-supplementary/S3_List of 937 statements deleted in the first round.docx]

List of 937 statements deleted in the first round

| 1 | Communication is a balance between a patient and the healthcare team |
| --- | --- |
| 2 | A nice communication gives a good initiative to work |
| 3 | Good communication saves time; nobody should wait longer |
| 4 | Doing things together is more efficient and timelier |
| 5 | With good communication, doctors and nurses will feel more supported to one another |
| 6 | If doctors have a good communication and relationship with nurse, it all makes it better |
| 7 | We work together nicely – it becomes easier and simple to achieve goals for patient management |
| 8 | We have better relationships when we do things collectively together on teams which will help to get know to each other |
| 9 | A good relationship has a big impact on the efficiency of the workplace |
| 10 | Changing way of doing things can make things easier and better |
| 11 | Doctors and nurses should think they should have the same team spirit to work together. |
| 12 | A small change that you make can make a difference in the efficiency of care that is delivered |
| 13 | If both sides feel that they can communicate freely, things will happen more efficiently |
| 14 | Things can evolve quickly with good communication |
| 15 | Doctors need to introduce themselves, their preferences, and expectances better with the people we work with |
| 16 | Use of name badge in the hospital makes a difference; it helps nurses and doctor to call other people by their name |
| 17 | Doctors should know their nurses or at least read their name on the batch |
| 18 | It makes a big difference if a doctor calls nurses by their names in front of the patient |
| 19 | The work would be easier if we know your team |
| 20 | It will be very helpful when you know your regular team. |
| 21 | Having to know your audience will help sometimes to get the best communication style. |
| 22 | Introducing team will give confidence to the patient that we all understand each other |
| 23 | If we have doctors, who are there for a few weeks, it is barely enough time to get know the staff, get along to make an impression |
| 24 | Communication gets difficult with locum doctors/nurses whom we have not met, as we are not sure of their clinical skills |
| 25 | We are more likely to have conflicts in wards with locum or seasonal staff, where we do not have the same understanding of the staff |
| 26 | Medical work is a team-based approach |
| 27 | Healthcare is a teamwork |
| 28 | Approaching patient care with a unified approach is important |
| 29 | The parents can put their frustrations back to the nursing staff, who can fire that back on to the doctors. |
| 30 | Empathic attitude to patients |
| 31 | If we have good working relationship, nursing staff are more likely to do things that need to be done urgently |
| 32 | Lack of trust may lead to undermine the teamwork or professional relationship |
| 33 | If the handover plans or concerns are not carried out, one might feel that they cannot trust the other party |
| 34 | Lack of trust may place an undue burden of responsibilities on one other to carry out the roles of the other party |
| 35 | A good communication allows gaps to be filled |
| 36 | If doctors are well communicating with the nurses, they will be able to know about the patient comprehensively |
| 37 | We need to work as a team to provide the highest quality care |
| 38 | Appreciation between the staff members – together as a team is important |
| 39 | Doctors view equality between team members |
| 40 | Nurses and doctors should act in a professional manner in front of patients |
| 41 | It would be reassuring if the doctors and nurses are both there when the key decisions are made |
| 42 | With joint rounds, things can be timely as any concerns can be addressed right at the bedside |
| 43 | When nurses and doctors are together in the rounds, there will be a comprehensive understanding of what is happening with the patient |
| 44 | If nurses attend the rounds with the doctors, they will be aware of the communication that a doctor is having with you |
| 45 | A 2-minute walk at the bedside with the nurse could help doctor as nurses can fill in the gaps between doctors and patients |
| 46 | Information obtained by nurses and doctors combinedly form a complete story of what is going with the patient |
| 47 | Nurse-doctor communication is integral to a deeper understanding of a patient |
| 48 | Doctors focus on the illness and medicine whereas nurses tend to look at more holistic picture of the patient |
| 49 | Medical staff get insight to the patient’s condition from the nursing notes |
| 50 | Nurses might have noticed something that the doctors may not notice during the ward rounds |
| 51 | Nurses’ communication of a patients’ situation helps a doctor helps take prompt action |
| 52 | The difference in the medical knowledge of different level of nurses plays an important part in patient care |
| 53 | Utilizing each other’s resource including those of allied health as a team promote patient outcomes |
| 54 | It should be a lateral platform between the doctors and the nurses |
| 55 | Communication helps nurses and doctors to work together rather than fighting about who is going to do it or who can do it. |
| 56 | We can easily address even the tiniest concerns that patients might have |
| 57 | Nurses can be more realistic, and doctors can be more idealistic in perceptions of what a patient can or cannot do |
| 58 | Nurses can read the note and take actions according to the order or advice of a doctor |
| 59 | Team focused approach involving the whole team and not just doctors making the decisions |
| 60 | We need to set up the boundaries and know when to help each other |
| 61 | Nurses and doctors have a different area of work, they should be valued given to all the beats of work |
| 62 | Nurses and doctors need to understand that they come from different level of expertise and everybody has something to offer |
| 63 | Medical staff need to understand what makes nursing care challenging |
| 64 | Staff members should understand each other’s thought process, and behaviour helps to achieve best outcome for the patient |
| 65 | Nurses and doctors need to understand the structure and role of each other |
| 66 | It is good to include a patient in his care if he can speak for himself |
| 67 | We need to be vigilant about what a patient is saying |
| 68 | The way people inform the things can impact another person |
| 69 | Nurses mostly hear the care plan from the patients as they are not having effective communication with the doctors |
| 70 | Doctors came on their round with other doctors, but I did not see any communication between the doctors and the nurses. |
| 71 | You do wonder about whether there was any coordination happens and how good that coordination was |
| 72 | All we can do is hope that doctors are communicating with nurses at some other locations where you cannot see it |
| 73 | It is important to convey message to all the staff to delegate the care task |
| 74 | Nurses can follow up the missing things recommended by the doctor |
| 75 | Doctors should ensure that the nurses properly understand the plan and ask if they have any queries or questions |
| 76 | When nurses have no idea of what is going on with a patient, they do not know what they are doing |
| 77 | There was a lot of time nurses saying they were not sure what is happening. |
| 78 | If nurses do not have the correct information, the hospital is not able to response appropriately. It delays in patient care |
| 79 | When communication is not clear, nurses need to focus on the patient care on whatever needs to be done quickly |
| 80 | Sometimes patient may not want to tell things to the doctor; nurses’ observations might help |
| 81 | Nurses’ can provide the valuable suggestions about the patient care which can accelerate care pathway. |
| 82 | Patient outcomes will be better with detailed communication |
| 83 | We need to improve ourselves to improve collaboration |
| 84 | When adequate information is not conveyed to the doctors, it could negatively impact the care provided |
| 85 | Communication help doctors to understand methods to handle the situation of a patient |
| 86 | Nursing staff are good resource for the doctors to rely on to highlight the issues that might affect the timely discharge |
| 87 | Nurses should be able to say to the doctors if they are not able to do or perform any tasks so that it could be delegated to some other person who would be able to carry out |
| 88 | Nurse-doctor communication is incredibly important in terms of patient care |
| 89 | Communication is a key factor to safe and good patient care |
| 90 | Communication is one of the important pillars in nursing practice |
| 91 | Nurse-doctor communication is very valuable to achieve a proper patient care |
| 92 | Communication between the different members of a health care team is vital |
| 93 | Intersectoral communication (with patients, relatives, government line agencies) is important to convey the message for prompt management of outbreaks |
| 94 | Communication must be good both ways for the best patient care in a hospital |
| 95 | Patient management can be altered with good communication |
| 96 | Nurse-doctor communication is important to monitor and assess the patient |
| 97 | A good communication will help to deliver whatever we intend to do |
| 98 | Collaboration is an important part of ensuring that we deliver good safe patient care |
| 99 | Nurses form the communication link between the medical team and rest of the disciplines |
| 100 | Nurses have an idea about which allied health professional would be better for the patient |
| 101 | Involving multiple partners (allied health professionals, social support workers, counsellors) in patient care can help |
| 102 | A holistic approach means that other professionals like dieticians, physiotherapists, occupational therapists are also communicating effectively with the medical and the nursing team |
| 103 | A good communication addresses the needs of the patient - physio |
| 104 | When doctors and nurses are communicating well, there is cohesion in the care delivery |
| 105 | If doctors can communicate nurses for smart goal settings, nurse can help patients to achieve those goal plans |
| 106 | Discussion with nurses helps preparing a care plan for a patient with chronic disorder |
| 107 | If nurse understand the plan, they can translate the plan with the patient |
| 108 | Nurse can have a big help to lifestyle modification, psychosocial impact, general health care, social home environment improvement of the patient |
| 109 | Nurses can help doctors by encouraging and convincing patient to modify lifestyle measures |
| 110 | Doing things together help to hear voice of each other to understand what is happening with the patient |
| 111 | Nurses and doctors can help each other managing and solving any complications |
| 112 | A good teamwork and leadership would be win for ourselves and for the patients |
| 113 | Very clear communication needs to go back and forth between all the different people |
| 114 | A clear allocation of responsibilities may help establish good communication |
| 115 | A clear overview of a plan, not just a list of tasks is effective for patient |
| 116 | Nurses and doctors should be willing to discuss patient’s needs or feelings with both parties |
| 117 | Junior staff in the ward more easily recognize the importance of keeping nurses in the loop and be more active in finding the nurse and letting them know about the plan of the patient |
| 118 | Nurses help doctor to monitor the patient’s telemetry and let the doctors know |
| 119 | Doctor should provide clear instruction to the nurses |
| 120 | They should openly express the ideas and opinions about the treatment method and everything |
| 121 | A doctor needs to liaise with the nursing team about implementation of (changes to) plan is important to optimise patient care |
| 122 | Doctor’s order should be clear, legible, and self-explanatory |
| 123 | If there is no communication, nurses will not be able to know what the red flags are |
| 124 | Nurses appreciate if at the end of the ward rounds doctor provide a summary of what is needed, or any specific changes have been made |
| 125 | Clearly explaining the nurses with parameters that a doctor is concerned of and not documenting only them on papers |
| 126 | Nurses provide the medical staff the information that they need to make their decisions day to day. |
| 127 | Doctors should value the nursing input and involve them in clinical decision making |
| 128 | Doctors should ask opinion from the nurses |
| 129 | Doctor is responsive, listen respectively, and either chooses not to undertake the suggestion and gives the rationale why or chooses to listen to the suggestion makes the environment conducive to open communication |
| 130 | Communicating a plan to a nursing staff and if they do not agree to it, listening to the reasons and deciding thing acceptable to both parties is the only way to progress it |
| 131 | Doctor should give attention to what nurses are saying as nurses are there with patient most of the times |
| 132 | The nurses’ thoughts of patient’s condition help doctor to manage the symptoms better and able to get through the diagnosis faster |
| 133 | A lot of doctors’ assessment is based on what nursing staff tell them |
| 134 | Doctors know when patients are ready for discharge from what the nursing staff tell us. |
| 135 | A good relationship with nurses makes the job of a doctor easier |
| 136 | Doctors should be self-caring to their patients |
| 137 | Nurses brief the patient’s concern and condition which helps a doctor to be prepared before seeing a patient |
| 138 | Its responsibility of a nurse to make a doctor aware of the patient’s situation and medical condition |
| 139 | Nurse needs to be clear communicating back (talk) to the doctor any concerns she has |
| 140 | With good rapport, a nurse will feel comfortable to come forward to tell their concerns about the patient |
| 141 | It is very important that results are conveyed in a professional, articulate way so that the doctor knows exactly what to do |
| 142 | Doctors want to know earlier if any patient needs an urgent review so that he/she can triage things |
| 143 | If anything goes wrong, the doctor needs to be informed straight away |
| 144 | Nurses have to read the patient; if something happens to a patient, everyone would be responsible |
| 145 | Nurses should pay attention to those issues that may have a big impact on recovery |
| 146 | Making sure that someone understood you well is massive thing |
| 147 | Nurse and the doctor should know what each other want (or are doing) |
| 148 | Having a better understanding of where each other are coming is very important |
| 149 | It is important for the nurses to know what is going on |
| 150 | There needs to be coordination of care |
| 151 | Nurses and doctors should understand clearly what it is wanted or needed by both sides |
| 152 | Trying to create a team environment rather than separating two teams is very important |
| 153 | Nurses and medical staff are not that great articulating what they want from each other |
| 154 | Patient would be reassured if we know that doctors and nurses are on the same page |
| 155 | Plan needs to be communicated properly before it goes to the patient |
| 156 | Discuss and agree on care plan |
| 157 | With an effective communication, doctors and nurses are aware of the alternative plans in emergency |
| 158 | We all should be communicative about the treatment plan, side effects and other management |
| 159 | Communication should be clear enough so that the nurses can treat the patient as mentioned. If it is not clear, they might do something that is not actually needed and may affect the patient |
| 160 | Nurses and doctors should be open to understand and step on each other’s shoes |
| 161 | Easy access to nursing and medical staff will help to discuss patient urgency |
| 162 | One should value the opinion of the other |
| 163 | Very young and older patients tend to deteriorate very quickly, we cannot wait very long for the doctors to see them |
| 164 | Information may not be passed out if doctors cannot find out nurses |
| 165 | Sometimes it is difficult for nurses to interpret and figure out what is wrong with patients |
| 166 | When there is a crisis in hospital or emergency when nurses have to participate, they might not be able to be around when the doctor comes in |
| 167 | There must be a proper channel for communication between nurses and doctors |
| 168 | Having the ward rounds with the nursing in-charge with you can help |
| 169 | Understanding the channels of escalation with the seniors of the team |
| 170 | Nursing staff should be aware of the available team structure to escalate the concerns if they do not get a satisfactory response |
| 171 | Taking ten minutes at the end of the round to talk with the nurse in charge to give an overview of what the plan is for the patients makes things easier |
| 172 | Patients can receive care based on the experience of senior people with the junior staff being able to ask the questions and get the support from nursing and medical team |
| 173 | It is the responsibility of a senior nurse to make sure that the junior doctors are doing their job |
| 174 | Communication with doctors can give autonomy for the nurses to care the patient |
| 175 | The doctor needs to have allocated time to speak to the nurses |
| 176 | Doctors should be available to answer the nurses’ queries |
| 177 | In aged care setting, doctors are not available at the time when nurses need them |
| 178 | Nurses and doctors should provide sufficient time |
| 179 | Evening handover between the nurse unit manager and medical team is a dedicated time to discuss and is very useful |
| 180 | Having a dedicated time where nurses, doctors, and other allied health staff can be physically present at the same space to talk about patient is most helpful |
| 181 | People may be confident talking to people who have leadership qualities rather than those who have just started their practice |
| 182 | Doctors take a directed history and can understand what is happening with the patient medically |
| 183 | Nurses feel easy to communicate with the doctors they know |
| 184 | Doctors should be approachable to the nurses |
| 185 | Sometimes being friendly can have detriment effects as people tend to ask more things or ask for favours with an approachable person |
| 186 | Doctors should be approachable to the nurses |
| 187 | Doctors need to encourage nurses when they are doing job nicely |
| 188 | In psychiatry, doctors should quickly be responsive to the concerns of the nursing staff to prevent being harmed agitated or aggressive patients |
| 189 | Doctors need to acknowledge the role nurses in patient care |
| 190 | Doctors acknowledge that nurses understand a patient’s needs |
| 191 | Good communication will help nurses feel free to ask any questions or doubts regarding their operation and management |
| 192 | One party or other may feel that they are not heard, or their concerns are not escalated or appreciated |
| 193 | Lack of having a grasp people’s skill levels can affect on what is being escalated to them and their understanding of it |
| 194 | If the concerns from nurses are dismissed by the doctors, they feel bad and think that can’t raise concerns in the future, which can impact a different patient |
| 195 | A nurse would refuse to talk with the doctors if they are rude or would not listen to |
| 196 | It is a responsibility of a nurse to stand up if they think if the drug chart is written incorrectly |
| 197 | Nurses freely asking doctors for something or even pointing out mistakes is important |
| 198 | A nurse should properly question on what is happening around because she has the duty of care to watch that patient |
| 199 | If nurses know something is wrong, there needs to be a potential avenue where they can escalate their concerns |
| 200 | Expect if the doctor has said something wrong, the nurse would pick me up and seek clarification in a graded assertive scale |
| 201 | If nurses do not carry out an order, they should not sign on that order |
| 202 | If some patient is presenting with behaviour of concern, nurses and doctors should work together to support those behaviour |
| 203 | It is important for nurses to escalate concerns to facilitate patient review |
| 204 | Sometimes nurses do enough to express what is going on negative repercussions (impact) for a patient’s care |
| 205 | A lot of communication between patient and nurses probably never end up getting communicated to the doctor |
| 206 | Nurses filter the information going to the doctor |
| 207 | A nurse can put forward her concerns and suggestions to the doctor in a way that he/she is not putting any authority on the doctor |
| 208 | It is important to have assertiveness in communication |
| 209 | These days, nurses are more assertive and often battle for their patients |
| 210 | Not having a power imbalance is an important aspect of good communication |
| 211 | Nurses and doctors need to be confident of what one another are doing |
| 212 | Having a meeting with a nurse is always helpful |
| 213 | There should be trustworthy relationship between nurses and doctors |
| 214 | Being more mindful and focused on what we are doing |
| 215 | Nurses do a lot of stuff (small procedures) for doctors |
| 216 | Nurses communicate with patient, reassuring the patient of what the patient is expecting |
| 217 | Nurses/doctors may not have time to read all the notes at all the times |
| 218 | Not everybody has the time to read all the notes that is written in EMR; plans might be missed then it just gets all over the place |
| 219 | Nurses can read the note and take actions according to the order or advice of a doctor |
| 220 | Nurse should have courage to question, ask or clarify information from the doctor |
| 221 | If a nurse can interact frequently with the doctor with the patient’s condition, it can help patient get managed promptly |
| 222 | It makes a difference when nurses are confident of communicating with the doctor to ask something |
| 223 | It is a right of a nurse to ask the question to advocate for the safety of the patient |
| 224 | Nurse and the doctor need to be able to ask questions to each other |
| 225 | If the communication is not cordial, people might step back of asking the question or communicating things related to the patient |
| 226 | Disclose/explain reasons behind care plan |
| 227 | Nurses are at better placed to raise questions asked when doctors are rushing |
| 228 | Nurses cannot just sit down and bear everything; nurses need to speak their part |
| 229 | When nurses find error in documentation by a doctor, they need to communicate with the doctor and get things sorted out to prevent bad things from happening |
| 230 | Hospital is not the place where nurses always follow the order of the doctors |
| 231 | When a doctor responsible for the patient care is not around, the nurse must approach a different doctor; patient is more important than which doctor is responsible for the patient |
| 232 | There is a direct impact if doctors can communicate the plan to the nurse |
| 233 | If we do not have enough nurses for a nurse to step in for the fifteen minutes when the doctor is there in the hospital ward, it is clearly an issue |
| 234 | If doctors reply to the nurses’ query, it is an easy way to transpose that information directly into the patient’s notes and can be easily passed on to the next doctor and nurse |
| 235 | We can get the information promptly with face-to-face communication |
| 236 | Good communication can help to track the progress of a task so that we can prevent a day being wasted |
| 237 | Doctors need to mould nurse to what your preferences are. So, they know you and how you work |
| 238 | (Doctor needs to) orientating the nurse, who is working with you, about how you like things to be done |
| 239 | Every person should mould and be shaped into with the ward culture |
| 240 | Setting expectations and a form or orientation when people start a new role or take a portfolio is important |
| 241 | The consultants are orientated, when they start in our department that the nursing team are the part of the whole team and need to be used as a resource and have the knowledge and information that might help them find their way. |
| 242 | As doctors work in different hospitals, they may not be aware of the hospital protocol and policies around a certain thing whereas the nurses know that stuff |
| 243 | Doctors often work on a setting (ward) where they have no previous experience, so they very much must rely on the nurses to show them how the system works |
| 244 | If documentation is carried out at the right place it will not take a long time to find it |
| 245 | It is of no point if you do not document whatever you talk with the patient |
| 246 | There needs to be a lot more of written communication which you can reflect or have a look at |
| 247 | Clear documentation of care plan at appropriate place so that nurse can easily read it and carry out things even when doctors are not available for explanation |
| 248 | Making sure the notes are eligible and written |
| 249 | If a verbal communication is not documented, it carries a risk of not being handed over to the next nurse |
| 250 | A verbal communication can be forgotten by either person as there is no record of it |
| 251 | A verbal miscommunication or mistake would ultimately lead to consequences and things happening which was not a part of the plan. |
| 252 | Things that cannot be communicated in a medical chart needs to be verbally communication and hand over |
| 253 | If there is verbal communication, patient care could be potentially rectified due to prompt action |
| 254 | A written thing needs to be verbally communicated |
| 255 | Clear communication in verbal language giving nurses chances to explain |
| 256 | A nurse calls doctor mostly for problem solving |
| 257 | When nurses communicate with the doctors, they need to make sure that the service is provided to the patient |
| 258 | A nurse may end up knocking at the door if she thinks that worth it |
| 259 | With a shift work for every 8 hours, information gets lost. So, we need to trust on what they are doing is right. |
| 260 | Doctors do not spend a lot of time with patients, |
| 261 | Patient willing to talk with doctors to know facts on their health |
| 262 | Patients could be clear if doctors communicate directly with them |
| 263 | Regular visits (interactions) with patients |
| 264 | Doctor would come early in the morning and then the rest of the care was left up to the nurses |
| 265 | Surgeon should look after the patient after the operation to see the outcome of the intervention |
| 266 | Doctors go to see the patient during the ward rounds or whenever the nurse contact us for any complication or other things that we must look for |
| 267 | Doctors always enquire a brief snapshot of what is going across the day whereas the nurses have a much large amount of time at the bedside |
| 268 | Communication should be timely and thorough |
| 269 | Time of day can influence communication |
| 270 | Time is the biggest factor influencing communication |
| 271 | People are more receptive to things at the beginning of their shift than towards the end of their shift |
| 272 | Doctors do not have set breaks like the nursing staffs |
| 273 | Doctors and nurses should be careful about how they communicate in front of the patients |
| 274 | Personal discussion should not be discussed in front of the patient |
| 275 | It is important to have an informal conversation to establish a good rapport; patient needs to be taken care before having such conversation |
| 276 | Efficiency will change massively if we can get the right person taking charge of the communication |
| 277 | Having nurse practitioners on the wards |
| 278 | There should be a strong nurse leader who can stand up on behalf of the nurses |
| 279 | If one person is doing one thing, it needs to be communicated with the team, so that the other members can focus on different things needed at that time (eg. Code blue – airway by one person, breathing by another and drugs by the third) |
| 280 | When the matter/request is urgent, keeping the key points could be helpful |
| 281 | We may not be able to provide adequate treatment to conditions we have not discovered |
| 282 | Patients complain that their queries are not addressed because the doctors do not listen to the nurses or the nurses do not tell the doctors. |
| 283 | If nurses and doctors cannot work together, it questions their professionalism |
| 284 | Communication between the entire healthcare team may go wrong |
| 285 | Breakdown in communication can affect the whole team |
| 286 | Disagreements with one person can affect the whole team |
| 287 | There can be misunderstandings |
| 288 | Miscommunication can lead to problems within departments. The department may be dysfunctional to the organization |
| 289 | We are not doing our job properly if we cannot address their emotional perspectives |
| 290 | It is good to have opinion because the other person can give right solution which is not coming in our mind |
| 291 | Disagreements can help pick up things that have been missed out through exchange of information between doctors and nurses |
| 292 | When there is a rift between doctors and nurses, other people involved in patient care often feel hesitant and get confused with their role in patient care |
| 293 | Communication can help nurses make aware of things they are not aware of or the doctor might not have thought that way |
| 294 | Few nurses do try to play a heroic role – trying to seek doctor’s help straight away |
| 295 | Doctor gets annoyed due to messages from the nurses which was their part of their care |
| 296 | It may cause stress to the doctor if nurses are repeatedly calling them when they are very busy |
| 297 | Over confidence of graduate nurses may end up being a medical issue |
| 298 | Nurses go through with the doctors sometimes too fast and too quickly. It may be because they are so busy. But I think they need to slow down little bit. |
| 299 | At times, nurses do not know the best way of communicating things with a doctor |
| 300 | It can also be a very challenging to get right communication |
| 301 | If doctors are not approachable to the nurses, things on the priority list can be dropped down |
| 302 | The culture of a workplace influences communication |
| 303 | When there are certain antagonistic things between doctors and nurses, for example yelling at each other, that obviously affect the patient |
| 304 | Personal disputes at home may be reflected during work which may hamper the communication |
| 305 | It is important to find out if they are having a problem in their personal life that is making them difficult to get along with work |
| 306 | Senior doctors think because they are senior, they can disagree with nursing assessments and judgements |
| 307 | A senior consultant might not appreciate the importance of keeping the nursing staff in the loop and the letting her know early about things that can affect the plan of care on the day |
| 308 | Seeing senior doctors communicate with nursing staff in a professional way can reinforce professional expectations of junior staff |
| 309 | We need to look as a team to address the personality clash |
| 310 | Communication depends upon which setting you are in |
| 311 | Personality traits of the medical or nursing staff can have a massive influence in communication |
| 312 | Attitude and communication skills of a doctor is key (important) as there is a power difference between doctors and nurses |
| 313 | Some of the doctors and nurses are very unapproachable and scary |
| 314 | There are doctors who have an aggressive personality and nursing staff who may not like a doctor |
| 315 | How a person handles stress is also important |
| 316 | Lack of communication may be due to ignorance |
| 317 | A busy day with complicated patients can make them less receptive to hearing about more complex plans and other jobs that we have asked them for |
| 318 | Break time can be a factor. Doctors and nurses go on breaks. Sometimes, we need to find each other but we cannot find them as they are on breaks and we do not want to disturb either. |
| 319 | When a nurse is busy, the doctors may not stop her to give a direct handover of the patient |
| 320 | When there are a lot of sick patients, everyone is stressed, and communication is worst in these cases. |
| 321 | When there is workload, the idea of working in a team- communication could be set aside because they have so much of the health care related works to provide |
| 322 | Workload of a doctor may impact their ability to do their job efficiently |
| 323 | Any personality clash should be recognized as a personality of an individual |
| 324 | Do not show your adversity (hard times) on other |
| 325 | If conflicts are taken personally, it would affect the whole profession |
| 326 | There needs to be a degree of psychological safety within an organization to create an environment facilitating the people to bring out the issues |
| 327 | We need to feel safe to talk to people |
| 328 | We cannot get on with everybody at our work, but we can work together to provide a good health care |
| 329 | Working with arrogant doctors, we need to find out a way to combat arrogance, pragmatism |
| 330 | Conflicts could end up in court |
| 331 | Nurses and doctors can lose their registrations |
| 332 | Lack of communication have a legal implication |
| 333 | Hospitals have taken some steps to control conflicts between doctors and nurses |
| 334 | Doctors should not show their frustrations with nurses |
| 335 | It there are disagreements, be direct, say that we have got a wrong foot here (acknowledge), and spend some time to repair that relationship |
| 336 | We can rectify disagreement by talking about the point of disagreement (listen, explain own views, and then act about the disagreement) |
| 337 | One should respect the concern on his/her side of the problem |
| 338 | We need to try to find out own’s weaknesses and strengths through regular meeting |
| 339 | Negotiation with the people having trouble communicating is the first step |
| 340 | If there is a breakdown in communication, we work through swiss cheese model to build those layers to prevent them from occurring |
| 341 | We should not assume that everyone has the same understanding of what is documented |
| 342 | It is important to self-reflect and see if there is anything wrong you have done |
| 343 | When we apologize for the mistake, the relationships are even better than neutral |
| 344 | Any misunderstanding needs to be cleared up, or sorted out so that all are on the same page |
| 345 | Even with disagreements, it is ok if you stay professional |
| 346 | Even with disagreement, we need to investigate together to check what is right for the patient |
| 347 | If both parties are prepared to change their behaviour, you can discuss that with them and explain the effect it is having to the rest of the team. |
| 348 | Asking directly giving an opportunity for an open discussion |
| 349 | Take time to explain what you are thinking |
| 350 | Talking openly about the situation is very important |
| 351 | Identifying the root cause of the conflict is important |
| 352 | Conflicts can be managed through direct face-to-face discussion |
| 353 | Should clear doubts in an empathetic way |
| 354 | Try not to insult or offend the person whom you are discussing with |
| 355 | Having non-confrontational ways to deal with interpersonal conflicts can help |
| 356 | At times, we do not good talking to each other but do better talking to our peers |
| 357 | We need to look for a solution that suites everybody |
| 358 | Getting an agreement is for the best interest of the patient |
| 359 | If we disagree on certain point of view, we need to agree before we can achieve 100% patient care. |
| 360 | If we do not come to an agreement regarding the patient care, we are not going to achieve the target care |
| 361 | Resolving the problems in a team will help each other to do best for the patient |
| 362 | People can gossip about disagreements between nursing and medical staff, which can create further problems within the department |
| 363 | Repeated disagreements over a same issue does not make a good working environment for nursing and medical staff |
| 364 | There is a lost opportunity for education between the two parties |
| 365 | We need to make sure that we are here to practice safety standards |
| 366 | Any significant issue that is highlighted by the nursing staff, sensitive in nature, should be addressed away from the bedside |
| 367 | Some of the discussions should not be done in front of the patient. It heightens their anxiety and stress levels. |
| 368 | They need to discuss anything discretely probably in an office or somewhere not in front of a patient |
| 369 | Talk with the supervisor, third person, or other colleagues who can solve the disagreement |
| 370 | If it becomes non workable, it is important to bring in people who are more senior to then discuss the issue |
| 371 | If a quick response is required in an important aspect of care, we can communicate with the senior members of the team |
| 372 | We need to improve the support that we receive from out hospital, directors, or the supervisors, whoever are in the hierarchy |
| 373 | Nursing staff might feel comfortable approaching a different member of the team highlighting the difficulties in communication with a particular team member |
| 374 | Trying to communicate directly sometimes can worsen the situation |
| 375 | Have a second opinion |
| 376 | Debriefs to seek point of view |
| 377 | If a nurse does not have courage to speak to a doctor, she could go to the senior nurse, who can speak on their behalf and make the connection |
| 378 | If it’s a fight between doctors and nurses, it is helpful for the senior staffs to get involved. Escalation to them earlier on can alleviate distress |
| 379 | During disagreements, it is beneficial to have coordinated discussions between the healthcare team or perhaps a senior member involved in the patient care |
| 380 | When a nurse feels to be confronted, she will ask another person to help her which is not the right way to communicate |
| 381 | Nurses should understand that it may be ok for them not to receive the answer the way they wanted from the doctors |
| 382 | If things are documented, they can be used for court of law if anything is used against them |
| 383 | Some of these conflicts can end up having trouble or mismanagement of a patient |
| 384 | Lack of trust/distress is the number one cause for hospitals being sued |
| 385 | Using shorthand or acronyms |
| 386 | Doctors usually have time pressure as they need to look after many patients |
| 387 | A lot of the work that doctors do are not physical but have mental pressure |
| 388 | Some doctors and nurses are bad communicators |
| 389 | Colleagues do not follow advice or recommendations |
| 390 | Older school doctors can be a problem |
| 391 | Sometimes even the most experienced people are not the best one ever. |
| 392 | Disagreements may occur because people do not realize that they are communicating badly |
| 393 | Knowing how to communicate with difficult persons |
| 394 | If people are coming to work with personal stress, even the mood of different staff can impact communication |
| 395 | Nursing stress level, their time commitments, the number of patients that they have got can influence communication |
| 396 | Hospital is already a stressful environment and with added pressure of working with a difficult person would add to anxiety and depression about going to work |
| 397 | As nurses and doctors work in rotations, what patient would hope is that there is mechanism in the ward routine for that communication to take place |
| 398 | Doctors need to change their attitude to comment or contribution from nursing staff |
| 399 | Some of the nurses might come from the culture where doctors are ranked very higher in the importance level than nurses and nurses do not have a voice |
| 400 | New doctors – interns, registers do not learn how to communicate |
| 401 | A young nurse may not have the skills to communicate the issues back to the doctor |
| 402 | Junior staff often do not understand the importance of communication in escalating concerns and identifying risk with patients |
| 403 | The number of junior staff can also impact communication as they are new to their responsibilities and they often do not know the best time and best ways to communicate to each other |
| 404 | Stressful situation and inexperience of a doctor might lead to doctors being dismissive in their communication |
| 405 | A junior doctor, will not feel respected if the nursing team is confirming the decision/plan with the other member of the medical team |
| 406 | Senior nurses may start implementing their own plans or teach the junior nurses on how to medically manage the patients which may affect patient care |
| 407 | Patients can be a barrier as they may prefer a specific person for communication. |
| 408 | If anyone miscommunicates, setting them aside could deliver a message that we need better communication and miscommunication is not tolerated |
| 409 | Not to judge based on different backgrounds and religion |
| 410 | Fostering the culture of unequal relationship will take time |
| 411 | It makes hard to set the right message when the senior management team cannot communicate with each other well |
| 412 | Nurses bullying doctors and doctors bullying nurses is a huge issue |
| 413 | Bullying is a huge issue from medical staff directed at nurses |
| 414 | There may be significant bullying from doctors over nurses, particularly when they are escalating things |
| 415 | Foreign nurses can sacrifice bullying to be able to build a life for their family in Australia |
| 416 | Among other forms of discrimination, racism plays a role in these environment |
| 417 | Sometimes, doctor may not communicate with a nurse due to racism |
| 418 | With bullying and not having good relationships, it is often difficult for having a good reference which can hold their career for a while |
| 419 | Elderly nurses with a lot of experience snub the new nurses who are fresh and just been out of school |
| 420 | Subtle imbalance of communication between nurses and doctors can create master-slave relationship |
| 421 | Many nursing staff are naturally intimidated by the medical staff |
| 422 | Nurse should not be bossy to the doctor |
| 423 | Nurse needs to be very kind to the student doctor |
| 424 | Hierarchical culture where nurses do not challenge the doctors prevents effective communication |
| 425 | With unequal relationship, one is always talking and the other never steps up to talk |
| 426 | We need to make sure that it is not a hierarchical communication with people feeling disempowered with the communication |
| 427 | Hierarchy and ego should not prevent the information being available so that patient can have the best care |
| 428 | Feeling of doctor is up there and nurses are subordinate can hamper communication |
| 429 | There should not be a class different between nurses and doctors |
| 430 | Doctors should not think that they are better than a nurse. |
| 431 | A change of way we look at things philosophy within the concept of being a nurse and doctor |
| 432 | With power imbalance, people will be defensive and dismissive; you do not constructively create better plan; and at the end we cannot create a definitive solution to a problem. |
| 433 | A surgeon was a god sitting up on the cloud |
| 434 | It can contribute to a detrimental hierarchical culture where both sides may not speak and listen to each other |
| 435 | Hierarchy is not so prominent in a rural hospital. It is not hard to ask help from a consultant. |
| 436 | A person/patient with different origin may not understand all the colloquialisms of the native English-speaking person |
| 437 | Lack of understanding due to accent can damage communication. Nurses cannot provide accurate information to the doctor and the doctor thinks they cannot rely on the nurse. |
| 438 | Even though the English level required for migrant workers is high but due to difference in accent, we encounter racism directed towards our clients |
| 439 | Miscommunication due to ethnic differences can be avoidable if we give sometime to understand the doctor or the nurse |
| 440 | We do not need to judge the background of the doctor. It is important to understand the information that is being provided by the doctor |
| 441 | There is a huge migrant flow in medical and nursing profession. Doctors and nurses come from the different parts of the world with different cultures in their system. |
| 442 | Language barrier can be an issue |
| 443 | Language skills and different ways of communicating of nurses can influence communication |
| 444 | We need to be aware that we mean the same thing with all our language that we use |
| 445 | Understanding and perception of patient safety or quality care can be a barrier |
| 446 | Decision making and critical thinking involved in prioritizing and identifying the care required influences communication |
| 447 | The way people communicate can be a positive experience for one and negative for another |
| 448 | Body language and tone is important in communication |
| 449 | There must be a protocol to resolve the conflicts |
| 450 | Negotiation during resuscitation or acutely deteriorating patient is harder |
| 451 | Concerns raised that are based on evidence or on their experience needs to be heard |
| 452 | Having one nurse allocated to two or three patients and if the same nurse covers the duty the next day so that they know the patients could make a difference. |
| 453 | Everyday the nurses and doctors might be working with different people, it adds extra challenges to communication |
| 454 | We do not feel happy with the job |
| 455 | A mucky day never makes anyone feel good |
| 456 | With disagreements, nurses and doctors will not enjoy their work |
| 457 | People might not be happy to come to work because of having tension with staff |
| 458 | Lack of good working environment can probably increase the turnover |
| 459 | On a long run, we will not want to come to work |
| 460 | Frequent arguments can impact their (nurses and doctors) career in the long term |
| 461 | There will be reduced satisfaction that comes out of the relationship |
| 462 | There will be reduction in the motivation to do things |
| 463 | May underperform in the job |
| 464 | Lack of motivation among nurses |
| 465 | We will not feel good mentally – it affects performance |
| 466 | It can hurt the confidence of doctor and nurses |
| 467 | Nurses are having more pressure if they are not having proper and correct communication |
| 468 | It may make feel the nurses that they are not been trusted enough, they might think that they are not good enough for work. They might feel isolated at times. |
| 469 | Feels like being undermined of your skills and is very frustrating |
| 470 | They might not be interested on work |
| 471 | With negative emotions we can forget things |
| 472 | With negative emotions we are not getting the exact things done |
| 473 | Nurses are the one who end up making the mistakes if there is not good communication |
| 474 | Being angry at job when we do not feel that sense of value |
| 475 | We lose your interest in the work if we do not feel comfortable working with a particular staff |
| 476 | Doctors and nurses may suffer from mental health conditions after having observed regular serious patient deterioration |
| 477 | Bullying can increase the risk of suffering from burnout, anxiety, depression |
| 478 | We cannot concentrate on our work |
| 479 | Nurses need to have a time off to relax |
| 480 | Having enough rest between work is important |
| 481 | People might come late for work |
| 482 | If people are not going to work because of having mental stress of going to work, we will have a lot of sick leave to cover. Overall, the organization will implode |
| 483 | People can become physically tired |
| 484 | Nurses may have more anxiety on issues with communication because of the power struggle between nurses and doctors |
| 485 | Nurses can leave the job because they cannot do it or keep making mistakes |
| 486 | People will feel emotionally tired |
| 487 | Australian health workforce is understaffed; it can lead to stress and pressure |
| 488 | People will feel mentally exhausted |
| 489 | Communication sometimes can be dependent on the roasters of doctors and nurses |
| 490 | Shortage of staff makes it very difficult to make time for good communication with the doctors |
| 491 | Long work hours will have a major impact on communication |
| 492 | Imbalance between the number of medical and nursing staff can affect communication |
| 493 | Doctors are not there in the facilities in the remote areas |
| 494 | Wellbeing of doctors and nurses is a big question |
| 495 | People can have open fights on the wards |
| 496 | Head budding between healthcare staff is not always in the best interest of the patient |
| 497 | The work environment can become toxic |
| 498 | People get upset if a person bully at you |
| 499 | Often nurses pick up the need of translators through their continuous interactions with the patient |
| 500 | Gender may play an important role in communication due to masochism in place in many hospital wards |
| 501 | I do not think gender of a nurse matters |
| 502 | Doctors and nurses need to have both academic and practical education |
| 503 | Upskilling training help nurses to develop confidence on their care |
| 504 | Feeling distrust with the clinicians is due to the difference in education and knowledge |
| 505 | People need to communicate better to overcome barrier due to knowledge gap |
| 506 | Communication depends on the level of education that we had on communication and on the level of your experience in the job |
| 507 | Training and cultural shift would help |
| 508 | Lack of the practical aspect of nursing care in the University may impact nurse-doctor communication |
| 509 | There should be proper identifiers whenever talking about a patient, the more the better |
| 510 | There are tools made to enhance communication between nurses and doctors |
| 511 | Notes, verbal (face to face), telephone or paging system |
| 512 | Having a system that allows for quick communication with minimal fuss can improve communication |
| 513 | For non-urgent situations that can be reviewed later, sending email or fax is the usual mode of communication |
| 514 | We can send an email to communicate. For example, sending a photo, which can be saved in the patient profile |
| 515 | Use of non-secure communications about clinical situations (texting, sending pictures over phone) may make things efficient but there may be concern to privacy of the patient’s information. |
| 516 | Maximising the use of available resources is important. |
| 517 | Having processes in place, not in antagonistic method, punitive way, when identified when there are conflicts between staff |
| 518 | Disagreements are common in a high stress setting; the way they are handled is very important in terms of how it affects patient’s perceptions and care |
| 519 | Highlighting the points of contacts between shift will allow nurses and doctors to have expectations of when they are communicating with each other |
| 520 | Doctors may miss some important things simply because there are so many pagers from the nurses |
| 521 | In rural areas, we need to talk to the doctors over phone, and sometimes if lucky, over video conference |
| 522 | In the rural areas, nurses expect to work the whole patient up, expected to know exactly what a doctor wants to know over telephone |
| 523 | White board was probably a good communication tool for the doctor as well as because the nurses are changing, and he can see the names of the nurses |
| 524 | We can make a phone call through our internal telephone |
| 525 | Pagers and phones can help us in conveying messages |
| 526 | LAN paging system will not allow a detail communication |
| 527 | Paging can be problematic because the pages fill up in a busy shift with requests and we need to cycle through them |
| 528 | A simple way such as, pop-up alert at the doctor’s screen, could be set up at every facility |
| 529 | Having a system that helps important things to pop up in the electronic records system so that they are not missed out |
| 530 | Innovative methods of communication – trial of application where nurses put information about the patient and their observations – could help |
| 531 | Developing universal system so that doctors can flag their important concerns for the patient will help to provide better care for the patient |
| 532 | If they had a free flowing communication channel/program that the doctor can check in even when he is not in the hospital on that would be helpful |
| 533 | There needs to be a system, an integrated system so that whatever the nurses write on the file, the doctor needs to get it from wherever he is, even though he is not at the hospital |
| 534 | Models of care (SIBR - Structured Interdisciplinary Bedside Rounds) with engagement of multidisciplinary team along with patients helps to improve patient care |
| 535 | There could be some rating systems – for nurses’ notes – low priority, medium priority, or high priority that the doctor must read, and they could tick the mark on like the triage systems |
| 536 | SBAR handover is good. But sometimes it is good to put the request at first |
| 537 | Methods for handover is encouraged and supported |
| 538 | Television in the recovery wards, icy poles, colouring pages, stickers could help relieve stress of paediatric patients |
| 539 | In rural areas, nurses do not use SBAR or any standard form of handover that frustrates our teams; because, then the nurse may not tell the doctor what she needed. |
| 540 | VHIMS (Victorian Hospital Incident Management System) should be initiated so that any incident goes into the hospital system in a formal way |
| 541 | Having a way to monitor if the use of technology is efficient for communication is important |
| 542 | There are programs that turns verbal words into writings |
| 543 | There can be mismatch of communication due to lack of adequate technology |
| 544 | People need to learn communicating in teams |
| 545 | Training people, educating people to speak about different pathways of advocacy |
| 546 | We can attend counselling programs that they might have |
| 547 | It is effective to have compulsory communication |
| 548 | Years of experience can positively or negatively affect the way they communicate and eventually affect the patient care |
| 549 | The number of years of experience will shape things but the environment or setting of a hospital can also affect communication |
| 550 | They should do things in the right way |
| 551 | Code blues or medcalls are run very well because of teamwork. |
| 552 | Our general health services is making client dependent rather than making them independent |
| 553 | Everyone should be aware of the policies or guidelines for communication that is based on the priority of patient care |
| 554 | Hospital can organize training on how to manage effective communication |
| 555 | Nurses and doctors should have training on how to communicate in specific situations –aboriginal communities |
| 556 | Professional development workshops can improve interactions between nursing staff and doctors |
| 557 | There needs to be opportunities to direct face to face communication between various staff |
| 558 | Support nurses and doctors at the same time without compromising the care of the patient |
| 559 | Intervention from hospital management to improve communication might work |
| 560 | Some hospitals may encourage or advocate to have good communication and working relationship with your co-workers |
| 561 | In some hospitals, communication between doctors and nurses may not be a priority |
| 562 | Having forums, team meetings, nurse-doctor meetings where we can talk about patient so that everyone knows |
| 563 | The healthcare team needs to meet regularly and include all staff |
| 564 | Before seeing the patient, they should read about the case and know what is exactly going on |
| 565 | They should discuss the case and what to expect before going to the client’s room |
| 566 | They should have discussions about you even when patient is not there |
| 567 | They should have more meetings about patient |
| 568 | If there is no communication, doctors may be angry for being called up later in the night because they have not clearly documented or handed over things |
| 569 | The doctors may be angry in the morning if the nurses have not communicated specific issues overnight and the patient becomes more unwell |
| 570 | Timely notification of adverse situation |
| 571 | With good communication between the doctor and nurse, any patient complaints can be dealt easily and openly |
| 572 | Delay in relaying information |
| 573 | Having a safe happy communication outside of those working hours talking about real interest on people you work with, asking about their life, asking about their interest, sitting down having a meal |
| 574 | Having a meal along with nurses in the tearoom helps to get to know them |
| 575 | Spending more time with the nurses makes a doctor lot more valuable and stops a lot of problem from happening as it is easier for people to access them |
| 576 | Having a teatime with multidisciplinary team go through the list of patients and their plan helps to identify the things needed to be done from different members makes things efficient |
| 577 | If they see each other at other places, they could greet each other and be on good terms |
| 578 | Spontaneous communication with nursing staff that does not involve patient care will make communication much more natural |
| 579 | In general, nurses and doctors do not tend to be friends with each other outside of the workplace |
| 580 | Friendships with nurses, by no means is common as friendship with doctors. |
| 581 | It would be nice, if everyone would be on the same footy and likely to be friends outside of work, whether they would be nurses or doctors. |
| 582 | Nurses and doctors should develop a sort of personal relationship with interest |
| 583 | Having a lovely, genuine friendship with the colleagues (nursing staff) at workplace |
| 584 | They should spend some time together outside of this working relationship |
| 585 | If there is a good team that we can get along, we always look forward to coming to the work |
| 586 | You get to know the team you are working with by getting mixed with them |
| 587 | Sitting with the nursing staff while having the lunch gives opportunity to know each other |
| 588 | Having gatherings to socialize with colleagues is helpful in creating good working relationship |
| 589 | Having a good relationship with nurses has a happy outcome |
| 590 | Building relationship over time to get to know each other professionally and personally can help to build respect and understanding |
| 591 | Having time away from the external stimulus to have speak one to one without interactions and distractions is helpful |
| 592 | We do not have to like or hangout with everybody at work. We just need to remain professional, respectful in communication with that person. |
| 593 | There needs to be appreciation of non-judgemental communication in workplace |
| 594 | Joking with each other, singing the song over the radio can comfort patient and make them relaxed |
| 595 | Having an open communication that is honest is important |
| 596 | Communication needs to be direct (face-to-face, over the phone, or writing a document) |
| 597 | Communication should be honest |
| 598 | Nurses and doctors should be willing listen to the other person |
| 599 | It is an enjoyable workplace if all the people feel valued and can contribute to patient care |
| 600 | Doctors should respect the nurses in the same way that the nurses respect them |
| 601 | They can hate each other outside the border of the hospital, but at the bedside they need to show unison respect to each other |
| 602 | Nurses and doctors need to be polite |
| 603 | We need to be kind with each other |
| 604 | They should remain professional and polite when one is proposing something |
| 605 | Communication needs to be professional |
| 606 | Doctors and nurses should discuss about patient’s special needs – asthma check, diabetic check |
| 607 | We need to make sure that there is a good level of mutual respect between all levels of health care professionals |
| 608 | We need to foster an environment that facilitates open communication |
| 609 | Patients will get better care when each party listens to the other |
| 610 | Understanding of how things are done changes the way we communicate |
| 611 | Understanding of how things are done changes the expectations of one another |
| 612 | Understanding of how things are done changes helps to be more supportive |
| 613 | Doctors and nurses should be open minded without any personal judgements |
| 614 | There should be a specific, dedicated line of communication with the patient |
| 615 | Doctors and nurses should look for different ways to enhance the journey of the patient |
| 616 | Having clinical meetings for every patient make sure that there is a client centre based three-way communication between patients, nurses, and doctors |
| 617 | With a good, tailored information patient will feel that they are able to decide with management |
| 618 | We cannot assume that every patient knows and understands what we are talking about |
| 619 | Even highly educated people can have a poor health literacy |
| 620 | Some patients may have additional disabilities which we need to consider |
| 621 | Patients do not have the knowledge that will help them self-medicate |
| 622 | Tailoring care to the needs of the patient gives them better experience |
| 623 | Tailoring communication to different groups of people can help |
| 624 | Doctors need to encourage nurses when they are doing job nicely |
| 625 | Environment is not going to take a bad view of a nurse speaking up will facilitate open communication |
| 626 | Nurses can help communicating with the patient while a doctor is doing surgery which makes a patient feels comfortable |
| 627 | Being around a team with consistency in the way they approach the care of the patient could make the patient feel secure. |
| 628 | If a patient perceives that they are not comfortable or there is a double standard in the management, it is going to be devastating |
| 629 | They might not feel confident, comfortable, or relaxed if they do not observe communication |
| 630 | Patient may not feel confident of your care as they can see the disharmony |
| 631 | Patients want to be listened to with my wishes/arguments |
| 632 | It is important to make people feel safe and cared for |
| 633 | The way they act, behave, communicate in front of patient can in fact, negatively impact patient |
| 634 | If communication is not timely, people might lose interest on their health issues (example, diabetic educator to a patient with newly diagnosed diabetes) |
| 635 | It is important to be kind towards each other and with the patient |
| 636 | We should realize factors that make each other’s job hard |
| 637 | Having empathy over each other’s job will make a big difference in patient care |
| 638 | Everybody should have their say and be respected no matter where they are in their education or ranks |
| 639 | It is important to use other forms of communication – visual, oral, pictorial, or video (you tube) etc so that they get that information to make an informed decision |
| 640 | We need to pull ourselves to a side (go aside) and discuss what is bothering us |
| 641 | If doctors do not agree with what nurses are telling, there is animosity which can go into a battle between nurses and doctors |
| 642 | In the punitive version, where you get rid of the people who are causing with problems are replaced with others, loses knowledge and experience |
| 643 | Educating doctors to delegating more responsibilities to nurses |
| 644 | We should pass on the problems that are recurring based on the institution’s policy |
| 645 | Adhering to the existing policies if any disagreements occur |
| 646 | We (doctors, nurses, and other staff) should have a meeting, once in a month to discuss how to improve communication. |
| 647 | Nurses need to do more things within the scope of practice |
| 648 | Sometimes, we can do things immediately but at other times, we might need to wait for the resources to come |
| 649 | We need to think about the resources, people, and the helping hands |
| 650 | Some patients may need visit to the emergency because of communication delay |
| 651 | When a person comes to the hospital, it is the job of doctors and nurses to help them get better and not to worsen their symptoms |
| 652 | Doctors often may not think about some important aspects in women, for example breast feeding, which may impact the management |
| 653 | Patients are the beneficiary of a good teamwork between doctors and nurses |
| 654 | Patient would be happy when leaving the hospital |
| 655 | If a patient is confident of what is in the chart is correct, he can probably relax more and help me get better faster |
| 656 | Understanding of what is going on decrease frustration |
| 657 | Any health interaction is provoking anxiety for most of the patients |
| 658 | Having a good relationship with doctors and nurses makes more comfortable |
| 659 | A good communication can soften the issue that they have to follow long term |
| 660 | If we do not communicate with the patients, we cannot build rapport with them. |
| 661 | We need to communicate with the patient openly to get the information on what they are going through |
| 662 | Patients expect respectful professional care |
| 663 | Language and ascent should be clear to the patient |
| 664 | Have interest on your patient |
| 665 | Patients expect them to be honest about everything related to their health |
| 666 | Nurses should relay the doctor’s voice to the patients |
| 667 | Effective explanation of expected outcomes would contribute to good communication |
| 668 | Clear instructions and expectations explained to the patient why we are undertaking any activities |
| 669 | I was not given any information till the next afternoon. I was not sure what would happen to me. A little bit more of communication would have been better |
| 670 | Patients want good explanation from both sides |
| 671 | Patients want to have a clear pathway of goals or what they can expect to be in the hospital |
| 672 | Patients will be aware of their plan with clear communication |
| 673 | Patients may be agitated, anxious and do not want to engage in communication. |
| 674 | Patients will hesitate to relay their concerns, communicate with health professionals and engage in their own reviews |
| 675 | Ensure things are attended |
| 676 | The frequency of visits by nurses was getting less as I was recovering from my surgery. I understand they had other patients to care, but sometimes I really needed them. |
| 677 | Lack of understanding of a medical term specially when we have a patient who is scared to ask for explanation in simple words. |
| 678 | Patients will feel the brunt of that broken team structure or communication |
| 679 | Patient is the person who will suffer for any poor communication between nurses and doctors |
| 680 | A patient can easily sense or know if the doctors and nurses are having conflicts |
| 681 | With bad communication, the priority is taken away from the patient care |
| 682 | Patients will not get cared if there is no good relationship |
| 683 | When there is a lack of communication, patients may not get proper advice, education, and support |
| 684 | If nurses and doctors do not communicate clearly to the patient, patient may not be familiar with their home care plan |
| 685 | Patients might be less likely to take advice from the medical team if there are disagreements between healthcare team |
| 686 | If trust breaks down due to lack of communication between doctors and nurses, there could be avoidance of accessing care for the preventive healthcare from GP |
| 687 | If patients do not trust the assessment or advice by a doctor, they are not going to follow it and their health will deteriorate |
| 688 | We can motivate the person continue the treatment depending on what kind of words what we use to tell them |
| 689 | Patient will not be interested in any activity if he is not feeling comfortable |
| 690 | There will be reluctance to come back even when things are not getting better with the initial management |
| 691 | They might seek out health care from another team. |
| 692 | Lack of communication can aggravate their physical condition as they are not able to trust medical staff |
| 693 | When patients are caught at the middle of the battle between nurses and doctors, they will lose confidence on treatment received |
| 694 | A negative communication shuts down all the options that may be available for the patients. |
| 695 | If the nurses need to chase the doctor to have things done, it takes off confidence from the patient |
| 696 | If there is animosity between nurses and doctors, patients will lose trust on the healthcare team and the hospital |
| 697 | If we do not get a good harmony at the bedside, patient may think that the team is not getting along with each other |
| 698 | If patients feel that you are not respecting the team, they will not respect the doctors which is not good for the quality care |
| 699 | People are angry for not being communicated end to end about technical errors |
| 700 | Patients need to be made aware of the disagreements between nurses and doctors |
| 701 | Patient may get disturbed with unclear information |
| 702 | The patient will be extremely concerned of why these people are conflicting |
| 703 | It is not good to have a conflict with a person who is looking after (you) |
| 704 | We can feel quite scared |
| 705 | You feel anxious and worried when you get conflicting messages from the different health professionals |
| 706 | Witnessing disagreements reduce patient’s trust on nurses and doctors |
| 707 | When there is disengagement with the patient, the right to access the quality healthcare is in jeopardy |
| 708 | When there is no respect between the clinicians, patients feel that their healthcare is in jeopardy |
| 709 | Patients are the sick persons who want reassurance and wants to know if they are going to get better; conflicts can have impact on them mentally |
| 710 | Patient would feel happier if there is good communication |
| 711 | Patient obliged to understand communication between nurses and doctors |
| 712 | Nurses and doctors need to be humble with patients and each other |
| 713 | An ideal patient care is provided when a patient feels that they have been looked after, not only by the best possible medicine, but also with the best possible behaviour |
| 714 | We need to share our plans as patients are anxious to know it |
| 715 | If a patient trust on a nurse or a doctor, they are more receptive towards their expressions |
| 716 | Patient feels comfortable if they feel free/comfortable to ask questions about the care provided to them |
| 717 | It provides a pleasant experience to the patient if they feel the whole team has the best interest on them |
| 718 | A great supportive, teamwork would be felt by the patients. |
| 719 | If patients see people around them in a positive mood, it helps their immune system |
| 720 | Patients care a lot about how they have been treated |
| 721 | Patients care about the person who took time caring them |
| 722 | If the family can see strong and helpful relationship between doctors and nursing team, it gives them the confidence to know that they are going to receive the best care. |
| 723 | The confidence in the medical care that you are getting is critical to recovery |
| 724 | We have to make the journey of a patient easier by communicating with them |
| 725 | If patients have a trust, they can approach to the doctors and nurses easily if they have any queries or questions |
| 726 | Patients should feel comfortable dealing with doctors and nurses |
| 727 | The doctors and nurses should always try to put themselves on the shoes or position of a patient |
| 728 | With making the patient understand what they are going through can improve greatly |
| 729 | Sorting out a way to communicate so that a patient could ask them if they were not sure of anything is nice, little extra thing when you are at home |
| 730 | We need to give people chance to ask questions and make sure that they have understood |
| 731 | Doctors should console the patient |
| 732 | Any conversation that might relax the patient and creates good atmosphere to the patient could be helpful |
| 733 | Nurses should humbly ask patients about their condition as every patient are not able to tell their condition |
| 734 | We need to give plenty of opportunities for them to ask questions |
| 735 | Doctors and nurses should not tell patient to stop asking |
| 736 | One of our responsibilities is to explain to the patient to align them back to the healthcare service |
| 737 | Nurses and doctors should think of ways to make patients understand – print, videos |
| 738 | Doctors should make the patients lead on the communication could lead to create more open-ended questions/discussion |
| 739 | Patients feel less empowered to ask questions if they see amicable communication between nurses and doctors |
| 740 | Patients can lose their autonomy |
| 741 | Patients can feel less empowered of themselves |
| 742 | As a healthcare team, we need to support the way a patient wants to live their life |
| 743 | With disagreements, we can seek the patient’s opinion before escalating the matter |
| 744 | We need to learn how to set our mind focus on patient care. Knowledge, awareness, and understanding is required |
| 745 | Need to ask accommodation, family, spiritual stuff, and patient’s wishes |
| 746 | Deep understanding of the social situation, social issues, and the personality of the patient |
| 747 | Nurses can have idea of what is best for the patient because of being with the patient for most of the time |
| 748 | Home environment – love, care, and affection |
| 749 | If we focus much on the pathology or the treatment of the illness, we could miss out the other psychosocial impacts, financial aspects of the patient care |
| 750 | It can delay patient care if plans are not implemented, or investigations are not carried out due to poor communication |
| 751 | Prolong time taken to be seen by the doctor |
| 752 | Doctor come back and see the patients before they leave the hospital |
| 753 | Nursing staff can access mood, social situation, and insights and understanding to these situations which doctors do not read |
| 754 | Patients feel better when a nurse communicated them with their family over phone |
| 755 | Patients were crying during COVID times as they did not had visitors. |
| 756 | Involvement of family is very important when patients cannot advocate for themselves |
| 757 | Engaging a family member or carer if there is difficulty establishing communication, for example, parents involved for paediatric patients |
| 758 | Patient’s family members also feel well supported in their care |
| 759 | Family members like prompt assessment and treatment |
| 760 | Nurses assist patient from their entry to the hospital |
| 761 | Patients sit down with the nurse to discuss a care plan |
| 762 | Compassion from the nurse |
| 763 | Nurses more responsible on care side |
| 764 | Good advice from the nurse |
| 765 | Nurses act as patient’s advocate particularly if there are any concerns raised by patients or family members |
| 766 | Nurses are the eyes, ears, and mouth for the patients |
| 767 | Nurses should advocate on behalf of the patient to discuss their concerns with doctors or other health professionals |
| 768 | Because of lack of communication, sometimes patients need to be advocate of themselves |
| 769 | Doctor would not understand some elements of patient care such as soft care |
| 770 | Things are delayed when nurses and doctors work on their separate teams |
| 771 | Delay in relaying information |
| 772 | Delay in review by the doctors |
| 773 | Problems with communication between doctors and nurses can leave with you pain or without pain management for significant amount of time |
| 774 | Disagreements also have ramifications for actual procedures |
| 775 | Patient care should be our main priority |
| 776 | A patient will not receive the best treatment if there is miscommunication between the nurse and the doctor |
| 777 | Healthcare is vital than communication |
| 778 | Making the patient feel that they are the centre of the care is one of the biggest things |
| 779 | Interpersonal relationships are not as important as the patient in front of you |
| 780 | If we do not come to a point that whatever we are doing is right, we are not achieving 100% care of the patient |
| 781 | We need to look not at the disease but look at the person |
| 782 | We need to look at the person and not at the disease itself |
| 783 | They should consider as a person rather than a patient |
| 784 | If there is no communication, it would seem like they do not know their work, they do not about the case |
| 785 | A patient may have to unnecessarily stay in the emergency for a longer time |
| 786 | Patients not being shipped to the wards from emergency if there is lack of communication |
| 787 | If the nursing staff do not communicate that a patient has arrived, they can sit in the wards for hours from emergency department |
| 788 | I was put into different wards and rooms because I could not find space; the doctors and nurses who were caring for me had no ideas of what was happening to me. They were confused and waiting for the information |
| 789 | Patient is not confident of their management when doctors and nurses are conflicting with each other |
| 790 | Patients will lose faith/trust with practitioners or health system |
| 791 | Not knowing what is happening obviously could add to anxiety |
| 792 | Disagreements can alleviate worry or anxiety |
| 793 | Patient would have felt clueless if there was no communication. |
| 794 | Patient would have got frightened by knowing that there is nobody to take care of. |
| 795 | The biggest worry is when the care of a patient is compromised due to the tension between doctor and nurse |
| 796 | People in the emergency room are probably having the worst day of their life |
| 797 | Patient alone in the hospital ward as relatives are not allowed except for a few specific times during COVID |
| 798 | Patient is lonely, vulnerable and does not have any support around me in the hospital |
| 799 | A patient does not want to be dismissed when he/she is vulnerable |
| 800 | Patients come to the hospital with sickness |
| 801 | Sometimes we need to accept what a patient wants by keeping aside our belief and values |
| 802 | We need to compromise a few things to make patients happy |
| 803 | It may be sometimes difficult to explain what a patient wants |
| 804 | We need to ask patients on what they want in their spiritual and social care |
| 805 | Understanding the social condition of the patient helps us to better manage the patient |
| 806 | Patients do not want doctors and nurses talk about themselves in a way that it can be listened by other persons in the hospital ward |
| 807 | There was no place anywhere where these confidential conversations could be had very easily |
| 808 | Having a suitable space to talk about something which is very personal like suicide attempts, like being homeless, is important |
| 809 | Coordination between the different services a patient receives should be in the same room at the same time |
| 810 | People might tell different stories to different health professionals, so everyone needs to be there at the same time. |
| 811 | Different people visit at different occasions to talk but not at the same time |
| 812 | Communicating between ourselves helps us not to ask the same question to the patient repeatedly |
| 813 | A lot of communication between doctors and nurses happens outside of the room, in the corridor |
| 814 | They gave me a sheet of information about what they are going to do |
| 815 | Patients can have multitude of repercussions |
| 816 | Potential deterioration of a patient can impact patient care depending on the skills of escalation and timeliness of the review |
| 817 | It can cause more problems or harms than good when there is less communication |
| 818 | It is not about having a bad result or a good result, but it is about having an appropriate result, the right one |
| 819 | There will be a drain on the patient’s ability to work |
| 820 | Patients may go home with issues which should have been addressed during their hospital admission |
| 821 | Patient can leave the hospital |
| 822 | People can come back from the hospital in worse conditions |
| 823 | With good communication patients can be discharged quickly |
| 824 | Good communication helps patient to get out of the hospital system as soon as possible |
| 825 | Proper communication will ensure ideal treatment methods and would be beneficial for the patient |
| 826 | There can be increased length of hospital stay due to deteriorations |
| 827 | Patient may not feel comfortable in the hospital setting anymore |
| 828 | People may have severe symptoms and refuse to go to the hospital – they will not receive any treatment – can increase mortality |
| 829 | It is nightmare for patients to be back and forth between doctors |
| 830 | If we give more pain to the patients, they will not want to come back to the hospital when they are sick next time |
| 831 | Test results will not be reviewed if there is lack of communication |
| 832 | Depending upon the doctor, nurse, and their way of communication there can be a delay in responding and reviewing the patient |
| 833 | With decreased communication, things take too long to be done |
| 834 | There may be lack of timely interventions |
| 835 | Patient may be given wrong medication, wrong dose |
| 836 | The physical ailments of our illness could be messed up with the mistakes are made |
| 837 | Treatment may not include the side issues or other underlying medical conditions |
| 838 | Patients might get deteriorate in the ward because they are not getting treatment, they are supposed to |
| 839 | There was a lot of waiting on the ward |
| 840 | Communication can affect the timeliness of getting right care at the right time |
| 841 | With good communication, things getting deteriorated get addressed in timely manner so that we can prevent further deterioration |
| 842 | Having things done promptly can promote responses to treatment |
| 843 | Timely delivery of care plan leads to prompt treatment and recovery |
| 844 | Missing out these tools in surgical wards may lead to wrong surgical intervention to the patient |
| 845 | Patients deserve for what they have paid for |
| 846 | We have to change the perceptions of the society that health is not a given thing |
| 847 | If the instructions are not clear it might affect on recovery stage |
| 848 | Timely care is important in our overloaded hospital systems |
| 849 | A friendly behaviour can make patient feel comfortable |
| 850 | If patients get a nice welcome at the ward, they get 50% better |
| 851 | It gives patient a clear indication of their own health requirements from early on in their hospital admission |
| 852 | Medicines delivered (provided) on time |
| 853 | Lack of communication may result in drugs are not given |
| 854 | Sometimes lapses in the communication leads to delay in newly charted medications |
| 855 | There are a huge range of minor complications |
| 856 | Adverse outcomes – infection, death, cross-contamination |
| 857 | Lack of open communication can cause trouble in patient care |
| 858 | Things are missed with poor communication which can lead to poor health outcomes |
| 859 | There may be greater risk of hospital acquired infections and problems, pressure ulcers or infections |
| 860 | Delayed response can cause more serious conditions in the patient or a decline in the patient’s condition |
| 861 | With lack of communication there could be a lack of observations on the patient |
| 862 | Consequences of not having an effective communication may lead to unwanted complications |
| 863 | Errors are prevented if you are an approachable person as someone can easily double check with you |
| 864 | Lack of communication can lead to fatal medical errors and catastrophic problems |
| 865 | When the plan is not there, the nurses call for the cover team, or the medical cover, specially at the night for example, it takes time to review and assess. |
| 866 | Lack of communication can be frustrating to the patient |
| 867 | It overall affects the quality of medical care the society will receive |
| 868 | At the end of the day, what type of service they provide matters |
| 869 | Disagreements that occur in front of the patient can reduce patient satisfaction in the care they are receiving |
| 870 | What happens to a patient in the hospital can change the trajectory of their life |
| 871 | It will create a negative image to the organization when a patient witness disagreement |
| 872 | Expected care may not be achieved |
| 873 | Not having communication will compromise a patient’s care |
| 874 | Patients may become aggressive as they do not want to stay in the ward |
| 875 | Once we discharge a patient to home, we do not know exactly what will happen afterwards if he does not come back to us |
| 876 | Mortality rates may increase |
| 877 | There could be lifelong repercussions even if they do not die |
| 878 | Family do not have sufficient income to cover for the unplanned prolonged hospital stay |
| 879 | An extra day in a hospital means, I have another day when I do not get paid, another day for me to organize things for my pets and other things |
| 880 | Its been a constant cost for me ($50 per week) |
| 881 | Private hospitals want patients to have the best experience |
| 882 | In public hospitals they are investigating appropriately, but not over investigating |
| 883 | Socioeconomic cost associated with health care industry |
| 884 | We will not be able to make room for the sicker patients |
| 885 | Prolong inpatient stay may affect other people who need hospital beds |
| 886 | Timely referral can help free up the patient beds |
| 887 | It is also not good for patient to be in the hospital as all patients cannot get their intense need (for example physio) |
| 888 | With good communication patients may find the process of being in the hospital more enjoyable |
| 889 | General flow of the hospital can be impacted |
| 890 | There is need of additional resources with prolong stay |
| 891 | Having disagreements will not be good for the health system |
| 892 | A good communication will affect the economy of the whole system – hospital or community |
| 893 | Prolonged hospital stay will increase cost to the Medicare system |
| 894 | Patient’s mental health, psychological wellbeing could be extremely negatively impacted |
| 895 | Good communication helps to provide not only the physical care but also the mental care and the functional care that might be needed |
| 896 | It can have a bad repercussion to patient care if nurse or doctor do not agree on something proposed by the either one |
| 897 | Private sector- cost saving rather than spending |
| 898 | Poor patient experience |
| 899 | If there is no proper communication, patients will not be assured that they are getting a proper treatment |
| 900 | The whole process can be vague and will bring unhappy patients |
| 901 | Lack of synchronization between nurses and doctors does not bring a good outcome |
| 902 | (Dissatisfied) patients can be confused about what is happening about their care |
| 903 | It will not give good impression to patients if they see nurses and doctors fighting with each other |
| 904 | Because of COVID, visitors are restricted, and communication is mostly dependent over phone; it mostly relies on nurses to communicate that |
| 905 | It can result in significant illness and injuries if there is no proper communication |
| 906 | More people will have negative experience on doctors, nurses, or specific hospital |
| 907 | If you are not comfortable with the care you are getting you are not giving the good reviews about the hospital or care services |
| 908 | Having disagreements will not be good to hospital reputation and goodwill |
| 909 | People do not want to go back to the same hospital or the doctor if they receive bad care |
| 910 | People will have doubt on the hospital or the system |
| 911 | Patients might not go back to the same hospital or look for a different one where they could feel comfortable |
| 912 | If there is no good treatment due to ineffective communication patient would not recommend the hospital |
| 913 | It will make feel the organization or facility that there is potentially wrong with the service |
| 914 | Good communication increases the reputation of the hospital |
| 915 | If there is good communication, people will recommend the hospital |
| 916 | Doctors and nurses can improve patient’s happiness and reduce stress by providing good customer service |
| 917 | A lot of factors – clean, hygienic, latest technologies, time for appointment, time for a waiting list, management of appointments, treatment methods will influence my decision for receiving healthcare |
| 918 | Infrastructure, services, equipment, hygiene, cleanness of the hospital needs to be improved. |
| 919 | We need to have a good preparation on sending the patient back from the hospital to the community |
| 920 | If we do not take preventive measures, we are not controlling the chronic condition |
| 921 | Nurses and doctors should put enough details on what is passed on for others to carry on the continuity of care |
| 922 | With effective communication, it is easier to follow up a patient on how the plan went |
| 923 | A good communication plan makes a GP visit a lot more efficient because everyone is on the same page |
| 924 | Doctors can communicate long term plans with a senior nursing staff member who can handover it to the nursing team is usually effective |
| 925 | Messaging through writing in the patient’s notes can be missed but is important to tell it |
| 926 | Having a debrief session after a stressful patient encounter can be helpful |
| 927 | More senior doctors and nurses can be rude to the juniors and may not want to listen to the other party. |
| 928 | It is demeaning and demoralizing as a junior when your practice is questioned in front of the patients and their families and over time it affects the self-esteem as a junior doctor |
| 929 | Pagers could potentially provide short messages which could be misinterpreted |
| 930 | We need to prioritize and say which one is the most important |
| 931 | Doctors need to have a friendly relationship with nurses (like a family) |
| 932 | Having nice communication in a friendly environment will help on a long run |
| 933 | It is important to have a respectful communication among team members |
| 934 | Coherent flow of information from the health professionals gives a positive vibe to a patient |
| 935 | Daily communication with the patients from nurses and doctors increases patient’s confidence |
| 936 | Having frequent disagreements affect the level of trust that we have on each other |
| 937 | If there is a clear communication about what we are doing and what they want, patients will have trust with us |
