# Supplementary material for: Stakeholders’ Perceptions of How Nurse–Doctor Communication Impacts Patient Care: A Concept Mapping Study
Source: Nurs Rep. 2023 Nov 6;13(4):1607–23. doi: 10.3390/nursrep13040133 (PMC10661264; doi:10.3390/nursrep13040133)
Supplement: Supplementary file 1 [file nursrep-13-00133-s001.zip › nursrep-2581629-supplementary/S4_List of 311 removed statements.docx]

**List of statements removed in second round**

| 1 - A bad hospital experience creates a lot of anxiety among the family members |
| --- |
| 2 - A breakdown in communication can lead to negative outcomes |
| 3 - A clear communication can settle the nerves of the patient |
| 4 - A cohesive team ensure better patient management |
| 5 - A collaborative team produces good positive health outcomes |
| 6 - A critical component to patient care is that nurses are more attentive to patients’ needs and give or spend more time with individual patients |
| 7 - A deficit of proper follow up appointment can contribute to repeated readmissions |
| 8 - A doctor's action would be reflected on the nurses and patients |
| 9 - A good communication can alleviate the concerns from early in the pace |
| 10 - A good communication can help patients to develop self-confidence |
| 11 - A good communication helps planning out rehabilitation beds |
| 12 - A good communication relieves panic inside your body |
| 13 - A good communication will help nurse to act on quickly |
| 14 - A good customer service can keep patients with you |
| 15 - A good customer service is important |
| 16 - A good handover provides synopsis of patient's condition |
| 17 - A good mix of nurse-doctor education or simulation can be better |
| 18 - A high patient load of the medical staff can account for delays in communication |
| 19 - A junior nurse/doctor may feel challenging to escalate the concerns about working relationship |
| 20 - A nurse provides emotional support to the patients |
| 21 - A patient does not have to repeat the same story if health professionals visit together |
| 22 - A sick patient wants to be comfortable |
| 23 - A specific sector of the community may not be able to express themselves openly to another sector of community |
| 24 - A welcome pack or some sort of information may make them comfortable |
| 25 - Adverse events can occur if the disagreements are time dependent |
| 26 - An informal communication to give an update about a patient can be helpful |
| 27 - An ongoing communication helps to pick up the subtle changes in the patient’s conditions |
| 28 - Any conflict needs to be settled down before patient management |
| 29 - Assistance if patient does not understand the doctors |
| 30 - At instances people may not be conscious enough to say that it is wrong |
| 31 - At instances we need to act on the situations to act/mould immediately |
| 32 - Bullying can increase the risk of mental health conditions (suicide) |
| 33 - Care can be delayed if nurses feel intimidated to ask the questions |
| 34 - Change in tone can indicate urgency in communication |
| 35 - Changes in plan does not get enacted due to disagreements |
| 36 - Changes in plan should be communicated to the nurses |
| 37 - Clear communication between patients, nurses, and doctors is important |
| 38 - Clear communication will help doctors and nurses to understand the needs of the patient |
| 39 - Clear documentation makes easy to carry out plans |
| 40 - Clear information about the after care needs to be passed to the regular doctor |
| 41 - Closed loop communication helps immediate action for timely management |
| 42 - Closed loop communication helps to identify the errors |
| 43 - Coming back to check if something has been done and making sure that someone understood you well is massive thing |
| 44 - Coming to a mutual agreement on which is the best way for us to work together |
| 45 - Communicating over the phone is difficult when the doctor does not have time to hear |
| 46 - Communicating with patient makes it easier for doctors to diagnose |
| 47 - Communication at right time so that a person is not distracted while they are doing something more important |
| 48 - Communication boards with information on staff delegation helps to talk with the person responsible for the patient care |
| 49 - Communication makes it clear what is wrong with the patient |
| 50 - Conflict resolution should be based on evidence-based practice |
| 51 - Consumer perspectives should be understood |
| 52 - Daily communication with the patients from nurses and doctors increases patient’s confidence |
| 53 - Delay in communication can deteriorate the patient’s health |
| 54 - Delay in treatment can impact the health system |
| 55 - Details of doctors should be readily available at the nursing station |
| 56 - Deteriorating health of a patient will put burden on the healthcare system |
| 57 - Different ways we can use communication to assist in the wellbeing of the patient |
| 58 - Directly calling the doctors increase interruptions to their work |
| 59 - Disagreement damages the perceptions over other profession |
| 60 - Disagreements can help pick up small things that sometimes can be missed |
| 61 - Disagreements can slower recovery rates |
| 62 - Disagreements may occur because people do not realize that they are communicating badly |
| 63 - Disagreements should be solved away from the patients |
| 64 - Do not ask doctors to see a patient until they think they need to see the patient |
| 65 - Doctor do not need to spend a lot of time reading the notes if nurses can communicate to the doctors at the morning ward rounds |
| 66 - Doctor must talk with nurses before disagreeing their idea |
| 67 - Doctor should listen to the information provided by the nurses |
| 68 - Doctors and nurses are the forefront of the healthcare practice |
| 69 - Doctors and nurses need to work together as a unit |
| 70 - Doctors and nurses should trust on each other’s knowledge, abilities, and expectations |
| 71 - Doctors are more responsive if can feel confidence on the nurses |
| 72 - Doctors are not aware of hospital protocols as they work in different hospitals |
| 73 - Doctors can benefit from the knowledge and experience of the nurses |
| 74 - Doctors have more information on patient’s conditions |
| 75 - Doctors need to acknowledge the role nurses in patient care |
| 76 - Doctors rely on the recommendation made by the experienced nurses |
| 77 - Doctors see their patients only for a brief time |
| 78 - Doctors should be sentimental of patient’s condition |
| 79 - Doctors should realize that nurses are valuable in-patient care |
| 80 - Effective communication benefits the patient, hospital, and everybody |
| 81 - Effective communication creates less confusion about patient care |
| 82 - Efficiency of nurses helps to undertake a procedure |
| 83 - Encouraging the patient to ask questions at any time |
| 84 - Errors can be prevented with joint rounds |
| 85 - Escalate this issue to the relevant manager to see if we could bring a resolution to workout |
| 86 - Every member of the healthcare team should be present while seeing the patient |
| 87 - Extra waste of time going back and forth to the hospital |
| 88 - Familiarity with the clinician helps in building rapport |
| 89 - Faster we can get them discharged safely, the better |
| 90 - Formal regular meetings between doctors and nurses |
| 91 - Frequency of care needs to be increased |
| 92 - Frequent disagreements on a long run can be stressful to the staff |
| 93 - Go above and beyond to make sure to make patient something special |
| 94 - Good communication can give nurses an opportunity to pass on important information |
| 95 - Good communication can improve hospital performance |
| 96 - Good communication elevate the mood of the patient |
| 97 - Good communication helps planning early and appropriate discharge |
| 98 - Good communication improves patient’s mindset and mental health |
| 99 - Good communication reduces gap between doctors and nurses |
| 100 - Grievance policy at the workplace can be utilized for conflict |
| 101 - Half of the information will be missed with improper handover |
| 102 - Happy patients will not complain about anything |
| 103 - Having a debrief session after a stressful patient encounter can be helpful |
| 104 - Having a robust communication system is a key to improve communication |
| 105 - Having a system for face-to-face communication for more urgent clinical things is important |
| 106 - Having a system that allows for quick communication with minimal fuss can improve communication |
| 107 - Having bad experience at hospital or clinic can impact for the rest of their life |
| 108 - Having frequent disagreements affect the level of trust |
| 109 - Having nice communication in a friendly environment will help on a long run |
| 110 - Having the patients’ best interest at front and centre is really the most important thing. |
| 111 - Head nurse needs to be involved in critical decision making |
| 112 - Hospital and staff need to learn from these mistakes to prevent these mistakes in future |
| 113 - Hospitals need to design the communication pathways (to improve communication |
| 114 - Hospitals should have one spokesperson to address the queries of the patient |
| 115 - If a patient goes home without a proper follow up or proper communication with their GP, it can lead to detrimental once they get home as something might not be covered or taken care of during the hospital |
| 116 - If patient is not communicated clearly, they may not follow the instructions given by the physician/GP |
| 117 - If there is a clear communication about what we are doing and what they want, patients will have trust with us |
| 118 - If there is no effective communication, patients may be missed out in the priority list |
| 119 - Important things need to be communicated verbally |
| 120 - Increased financial strain (may occur due to unplanned prolong hospital stay) |
| 121 - Increased need of investigations and specialist consultations |
| 122 - Information can be lost if one party does not listen to other |
| 123 - Institutional factor could affect communication between doctors and nurses |
| 124 - Interpersonal issues could affect communication |
| 125 - It can be challenging for the nursing staff to get to know people with high turnover |
| 126 - It can impact the cost effectiveness of treatment |
| 127 - It is demoralizing as a junior when your practice is questioned in front of the patients |
| 128 - It is important for nurses and doctors to ask patients what they want |
| 129 - It is important to have a respectful communication among team members |
| 130 - It is not good to have complications due to lack of communication between nurses and doctors |
| 131 - It takes a long time to get back that confidence |
| 132 - It will increase the out-of-pocket expenses |
| 133 - Joint rounds can help providing prompt patient care |
| 134 - Judicious use of electronic records is required |
| 135 - Lack in timely intervention can escalate the situation |
| 136 - Lack of communication can create confusion and distrust on health professionals |
| 137 - Lack of communication can lead to a lot of complicated situations |
| 138 - Lack of communication may affect the reputation of the institution |
| 139 - Lack of communication may impact the quality of care received by the patient |
| 140 - Lack of communication may lead to missing information that patients might have talked about |
| 141 - Lack of communication may result in some treatments not being carried out |
| 142 - lack of communication prevents a comprehensive holistic patient care |
| 143 - Lack of effective communication makes care fragmented |
| 144 - Lack of trust increases distress among patients |
| 145 - Level of training can influence communication |
| 146 - Little things that we do make a positive experience |
| 147 - Lot of people do not have a choice between public and private hospital |
| 148 - Medical insurance overbilling |
| 149 - Miscommunication due to ethnic differences can be avoidable if we give sometime to understand the doctor or the nurse |
| 150 - More negative feedback makes rating of the hospital becomes low |
| 151 - More people will lose their jobs when health system is down |
| 152 - Need to evolve role and responsibilities of the nurses |
| 153 - Negative emotions can make prone to make errors |
| 154 - Negative interactions between nurses and doctors difficult getting good outcomes for the patient |
| 155 - Negotiating disagreements in a professional manner |
| 156 - Nonverbal communication cues as well are important |
| 157 - Not feeling safe will lead to high level of burnouts |
| 158 - Not having a good communication creates hostility in the workplace |
| 159 - Nurse make the communication between the patient and their family member |
| 160 - Nurse-doctor communication can expediate care |
| 161 - Nurses and doctor should check if a patient has understood what has been told |
| 162 - Nurses and doctor should understand urgency of the situation |
| 163 - Nurses and doctors complement each other |
| 164 - Nurses and doctors need to read each other's note |
| 165 - Nurses and doctors separately communicate with patient |
| 166 - Nurses and doctors should discuss with the patient about the available options for management (dialysis, antibiotics) |
| 167 - Nurses and doctors should have open communal space where can know each other |
| 168 - Nurses and doctors should properly escalate their concerns with each other |
| 169 - Nurses and doctors should talk with the indemnity insurance to solve the problem (legal issues) |
| 170 - Nurses can act on behalf of the doctor if there is well documented plan |
| 171 - Nurses can easily make error due to lack of communication |
| 172 - Nurses can help provide a better plan for patients to manage their chronic illnesses |
| 173 - Nurses help carry out the recommendations made by the doctor |
| 174 - Nurses help patients to have their concerns passed on to the doctor |
| 175 - Nurses help understanding the underlying reason of the care |
| 176 - Nurses identify the need for the patient missed out by the doctors |
| 177 - Nurses make sure that the nutritional needs of the patients placed into their care plan |
| 178 - Nurses may not act upon the plans and requests made by the doctors |
| 179 - Nurses may not identify the clear issues because that might come down to necessarily not knowing what was wrong with the patient or of poor assessment |
| 180 - Nurses need to learn quickly on how to communicate with each other |
| 181 - Nurses need to tell things in a friendly way so that others can take it easily |
| 182 - Nurses play an important role in preventive measurements/medicine more than a GP |
| 183 - Nurses prepare the ground for the doctors |
| 184 - Nurses should be serious about vital signs |
| 185 - Nurses should have skills to report the concerns of a patient |
| 186 - Nurses should provide accurate information to the doctors |
| 187 - Nurses should speak up if they think doctor is wrong |
| 188 - Nurses should try to navigate in a different way rather than getting frustrated. |
| 189 - Nurses should verify doctor’s order |
| 190 - Often nurses explain the difficult conversations (between nurses and doctors) with the patients |
| 191 - One negative incident can stand out all positive communications |
| 192 - Ongoing disagreement create an ongoing disparity in health which may not be limited being local, but can be state level and global perspectives |
| 193 - Online messaging system with patient identifier could be better |
| 194 - Open communication between nurses and doctors is important |
| 195 - Overflow of hospital beds may increase stress to the staff |
| 196 - Pagers could potentially provide short messages which could be misinterpreted |
| 197 - Passing on the notes and information so that the patient does not need to repeat |
| 198 - Patient can potentially even die if procedures are not done due to disagreements |
| 199 - Patient can withdraw from coming back to the hospital |
| 200 - Patient care is a multidisciplinary approach |
| 201 - Patient may be left untreated until it is late |
| 202 - Patient needs to be the centre of attention when they are having conversation in front of the patient |
| 203 - Patient often disclose their concerns more to the nurses than to the doctors. |
| 204 - Patient wants the health care team that is supporting each other |
| 205 - Patient will feel unhappy if there are unresolved conflicts |
| 206 - Patient will not have any motivation towards a proposed therapy |
| 207 - Patient’s expectations are meet in a private facility, but at the financial cost to yourself |
| 208 - Patients are not good educated to seek for the health advice |
| 209 - Patients are unhappy about the lack of communication about technical errors |
| 210 - Patients can be exposed to hospital acquired infections |
| 211 - Patients can develop disabilities |
| 212 - Patients care about how people communicated with them |
| 213 - Patients do not have access to the same information that nurses, and doctors have |
| 214 - Patients expect communication to be in writing, so that they can access it whenever they want |
| 215 - Patients expect doctors (and nurses) to show empathy |
| 216 - Patients feel uncomfortable when doctors outrage nurses |
| 217 - Patients from rural community want to stay with their family |
| 218 - Patients get involved in their care if they feel comfortable with doctors and nurses |
| 219 - Patients may decide not to do things that are beneficial to themselves |
| 220 - Patients may have problems when there is no good linkage of handovers between nursing and medical staff |
| 221 - Patients may not receive procedures, medications that they need to receive |
| 222 - Patients prefer going a hospital that provides prompt service |
| 223 - Patients will not experience a full benefit |
| 224 - Patients will not get proper treatment if there is a breakdown in communication |
| 225 - Patients will not provide information if they do not feel comfortable communicating with nurses and doctors |
| 226 - Patients will not receive optimum care with disagreements |
| 227 - Patients will receive poorer care due to miscommunication |
| 228 - Patients’ perceived rejection may lead to self-harm or attempted suicide |
| 229 - People brought up in a system where they do not ask any question to a doctor |
| 230 - People may share negative views about the experience with other people |
| 231 - People might come late for work |
| 232 - People need to prioritize art of communication in their education |
| 233 - Personal opinions (should not) influence patient care |
| 234 - Personal safety comes first in hostile environment |
| 235 - Personal style of communication can impact communication |
| 236 - Problems in escalating things to intervene may lead to late diagnosis and more serious complications |
| 237 - Professional care can enhance recovery of patient |
| 238 - Prolong stay of a patient will affect the budget of the hospital |
| 239 - Prolong stay will prevent other patients to get service from the hospital |
| 240 - Providing a safe environment for the patients to make them speak up |
| 241 - Recognizing how skills from different persons can gives best experience to the patient |
| 242 - Relationship between doctors and nurses can affect communication |
| 243 - Repeated disagreements reduce productivity of the department and staff |
| 244 - Reviews on the social media gives an active impression about the hospital culture |
| 245 - Risk perception may influence what/how we communicate can be a barrier |
| 246 - Senior doctors and nurses can be rude to the juniors |
| 247 - Setting up the expectation from each other through regular conversation |
| 248 - Society needs to take the ownership of health |
| 249 - Some of the stuffs are best managed by the nurses for example |
| 250 - Speaking out with a colleague lets out your frustration |
| 251 - Spiritual factors also could influence communication |
| 252 - Staff knowing your name – comforts patient patients are not a number |
| 253 - Staff should not communicate with a loud voice as there are sick people around them |
| 254 - Team morale can be influenced by the level of communication between nurses and doctors |
| 255 - The attitudes and behaviour of both nurses and doctors in public and private healthcare sector is quite different |
| 256 - The doctor needs to know what is happening to his patient |
| 257 - The dynamics of nurse-doctor communication impacts patient care |
| 258 - The health authority should promote communication |
| 259 - The hospitals should have an eye on the cleaning services |
| 260 - The managers should have to look at the conflict to improve the communication |
| 261 - There can be a gap in the knowledge if we are talking to people with different level of education |
| 262 - There can be a lack of communication between what a doctor wants to have and what a patient can afford to do (for example, a doctor wants a patient to have insulin three times a day, but the patient can afford to have it once a day) |
| 263 - There could be policies to empower nurses |
| 264 - There is a fine line between having disagreement and conflicts |
| 265 - There is language barrier with indigenous population |
| 266 - There is potential for harm if poor communication or the concerns of one another are missed |
| 267 - There should be a facility-based agreement on what to do in case of disagreements |
| 268 - There should be a planned review with medical and nursing team |
| 269 - There should be a three-way conversation with having the inputs from the patients |
| 270 - There should be good communication link between the hospital and structures within the community |
| 271 - Things can be missed out if not communicated |
| 272 - Things will be flagged quicker with a good communication |
| 273 - Tone between nurses and doctors influence communication with the rest of the staff in the ward |
| 274 - Transparent communication on decision making of the patient |
| 275 - Trying to balance between work, family responsibility and doctor has been a nightmare |
| 276 - Underlying stress level of a person can impact communication |
| 277 - Unequal power between the nursing and the medical staff play an important role in communication |
| 278 - Uneven distribution of doctors and nurses can cause delay in care |
| 279 - Use of sign language may not be clear to the patient |
| 280 - Using a structured communication is very helpful |
| 281 - Using patient identifier could improve communication |
| 282 - Using shorthand or acronyms |
| 283 - Utilizing nursing workforce in health avenues could enhance patient care |
| 284 - We cannot give the right treatment if we are not communicating |
| 285 - We need to be harsh to improve communication |
| 286 - We need to communicate with others of what we are doing |
| 287 - We need to deal each patient as a person |
| 288 - We need to flag the urgency of what we are discussing |
| 289 - We need to follow protocol to report something that is wrong |
| 290 - We need to handle the situation mutually |
| 291 - We need to look for the social determinants of health that needs to be improved |
| 292 - We need to make a standard, reinforceable means of communication |
| 293 - We need to make sure that we are using the culturally appropriate communication |
| 294 - We need to prioritize which one is the most important |
| 295 - We need to think about the resources, people, and the helping hands |
| 296 - We should include patient and family members in the treatment |
| 297 - We should not insult or offend the person whom you are discussing with |
| 298 - Wearing masks in the healthcare setting could influence hearing |
| 299 - What doctors see might not be representative of how things are going more consistently |
| 300 - When nurses and doctors do not sense any urgency in the patient during their communication, then the patient tends to stay longer |
| 301 - When patient’s concerns are addressed, they have a better chance of not being readmitted |
| 302 - When the stay of a patient is prolonged, the government needs to pay more for the patient |
| 303 - With a good communication we can identify other problems that concerns a patient |
| 304 - With clear communication tests would be done quicker |
| 305 - With conflicts, nurses may not feel comfortable to talk with the doctor |
| 306 - With good communication there will be increased turnover of patient’s beds |
| 307 - With good communication, patients will come back to the same hospital in future |
| 308 - With good communication, patients will get right medicine |
| 309 - With good support, patients might feel like they are not away from the home |
| 310 - Wounds can get infected if not dressing is not done properly |
| 311 - Wrong decisions could be made based on those wrong information/report |
