# Supplementary material for: Stakeholders’ Perceptions of How Nurse–Doctor Communication Impacts Patient Care: A Concept Mapping Study
Source: Nurs Rep. 2023 Nov 6;13(4):1607–23. doi: 10.3390/nursrep13040133 (PMC10661264; doi:10.3390/nursrep13040133)
Supplement: Supplementary file 1 [file nursrep-13-00133-s001.zip › nursrep-2581629-supplementary/S5_List of 69 statements.docx]

List of 69 statements selected for structuring phase of concept mapping

|  | **Name of the statement** | **Random number*** |
| --- | --- | --- |
| 1 | A structured handover between nurses and doctors is important | 29 |
| 2 | Advice from nurses help doctors to plan patient care | 7 |
| 3 | Bad communication between nurses and doctors may be traumatic for the patient | 43 |
| 4 | Clear allocation of tasks to nurses and doctors | 45 |
| 5 | Clear and detailed clinical documentation is an important aspect of nurse-doctor communication | 61 |
| 6 | Clinical problems can only be addressed through positive nurse-doctor communication | 9 |
| 7 | Clinicians with a heavy caseload can be less effective at communicating | 33 |
| 8 | Clinicians with more clinical experience are better at communicating | 31 |
| 9 | Clinicians have a different scope of practice | 10 |
| 10 | Clinicians need to be approachable | 27 |
| 11 | Communication is enhanced if nurses and doctors have consistent shifts (working hours) | 12 |
| 12 | Communication is improved if nurse and doctors spend time getting to know each other | 21 |
| 13 | Conflict can negatively affect the clinician’s wellbeing | 57 |
| 15 | Delayed communication can lead to frustration | 63 |
| 16 | Direct (face-to-face) communication reduce delays in patient care | 19 |
| 17 | Dissatisfied patients will disengage with healthcare services | 54 |
| 18 | Doctors and nurses need to be honest with patients | 42 |
| 19 | Doctors and nurses need to use language that can be understood by the patient | 40 |
| 20 | Doctors need to lead nurse-doctor communication | 68 |
| 21 | Doctors need to make sure that the instructions they give to nurses is understood | 15 |
| 22 | Doctors' use of medical "jargon" impacts understanding by nurses | 46 |
| 23 | Effective communication is a skill that needs to be taught when nurses and doctors are in training | 65 |
| 24 | Effective nurse-doctor communication ensures timely patient care | 13 |
| 25 | Effective nurse-doctor communication improves the quality of patient care | 4 |
| 26 | Finding time for informal discussions about how to improve patient care is important | 24 |
| 27 | Good communication between doctors and nurses can comfort patients | 53 |
| 28 | Good communication is important across all shifts (including nights) | 17 |
| 29 | Good communication will improve people’s faith in medicine | 1 |
| 30 | Good interdisciplinary communication will ensure that discharge plans are meaningful | 52 |
| 31 | Good nurse-doctor communication reminds clinicians what tasks need to be completed | 5 |
| 32 | Having English as a second language may impact nurse-doctor communication | 59 |
| 33 | Important information about patient care gets lost if communication is poor | 14 |
| 34 | Nurses and doctors need be good at communicating with family members | 2 |
| 35 | Nurses and doctors need to have a good understanding of current evidence-based practice guidelines | 66 |
| 36 | Nurses and doctors need to make sure that they do not discuss patient care where they can be overheard by people not directly involved in their care | 37 |
| 37 | Nurses and doctors need to provide multidisciplinary patient care | 3 |
| 38 | Nurses and doctors need to trust each other’s capabilities | 6 |
| 39 | Nurses and doctors should discuss care plan before seeing the patient | 25 |
| 40 | Nurses are a bridge between patient and the doctor | 11 |
| 41 | Nurses need ensure they are aware of change in patients care plans | 8 |
| 42 | Nurses need prioritise care that impacts patient recovery | 67 |
| 43 | Nurses need to lead nurse-doctor communication | 16 |
| 44 | Orientation of new staff improves effective nurse-doctor communication | 20 |
| 45 | Patients are more likely to complain if they witness poor communication between nurses and doctors | 58 |
| 46 | Patients can be discharged before they are ready | 50 |
| 47 | Patients can get wrong treatment | 48 |
| 48 | Patients can influence communication between nurses and doctors | 38 |
| 49 | Patients need to fully understand their care and treatment | 28 |
| 50 | Patients tend to share more information with nurses than doctors | 69 |
| **51** | Personal issues (e.g., family stress) can impact communication | 30 |
| 52 | Personal Protective Equipment (PPE) is a barrier to effective communication | 34 |
| **53** | Poor communication between nurses and doctors may lead to people taking time off work | 56 |
| 54 | Poor communication can lead to worse health care outcomes in the longer term | 44 |
| 55 | Poor communication may increase the chances of a patient needed to be readmitted | 47 |
| 56 | Poor communication may mean that patients are not clear about the self-care behaviours they need to change | 39 |
| 57 | Poor communication may mean that patients are sent to an inappropriate clinical setting | 36 |
| 58 | Poor communication may mean that patients do not get required interdepartmental consultation on time | 51 |
| 59 | Poor communication may prolong a patient's period of hospitalisation | 41 |
| 60 | Precise communication is required in emergency situations (e.g., cardiac arrest) | 23 |
| 61 | Senior clinicians need to proactively help resolve conflicts between nurses and doctors | 64 |
| 62 | Technology can be used to improve communication between nurses and doctors | 55 |
| 63 | The quality of communication between nurses and doctors can influence the ward atmosphere | 26 |
| 64 | The severity of a patient's condition can impact communication | 22 |
| 65 | The volume of information shared between nurses and doctors can impact understanding | 32 |
| 66 | Unprofessional conduct (e.g., shouting) between nurses and doctors needs to be reported | 60 |
| 67 | Using the clinicians "name" in discussion improves communication | 18 |
| 68 | When vital information is not communicated, it can lead to increased risk of mortality | 49 |
| 69 | Workplace bullying impacts communication | 35 |

* A random number was generated for the structuring task and corresponds to the statement in the concept map.
