# Supplementary material for: Stakeholders’ Perceptions of How Nurse–Doctor Communication Impacts Patient Care: A Concept Mapping Study
Source: Nurs Rep. 2023 Nov 6;13(4):1607–23. doi: 10.3390/nursrep13040133 (PMC10661264; doi:10.3390/nursrep13040133)
Supplement: Supplementary file 1 [file nursrep-13-00133-s001.zip › nursrep-2581629-supplementary/S6_List of concept maps produced by Ariadne software package.docx]

**Two-cluster solution**


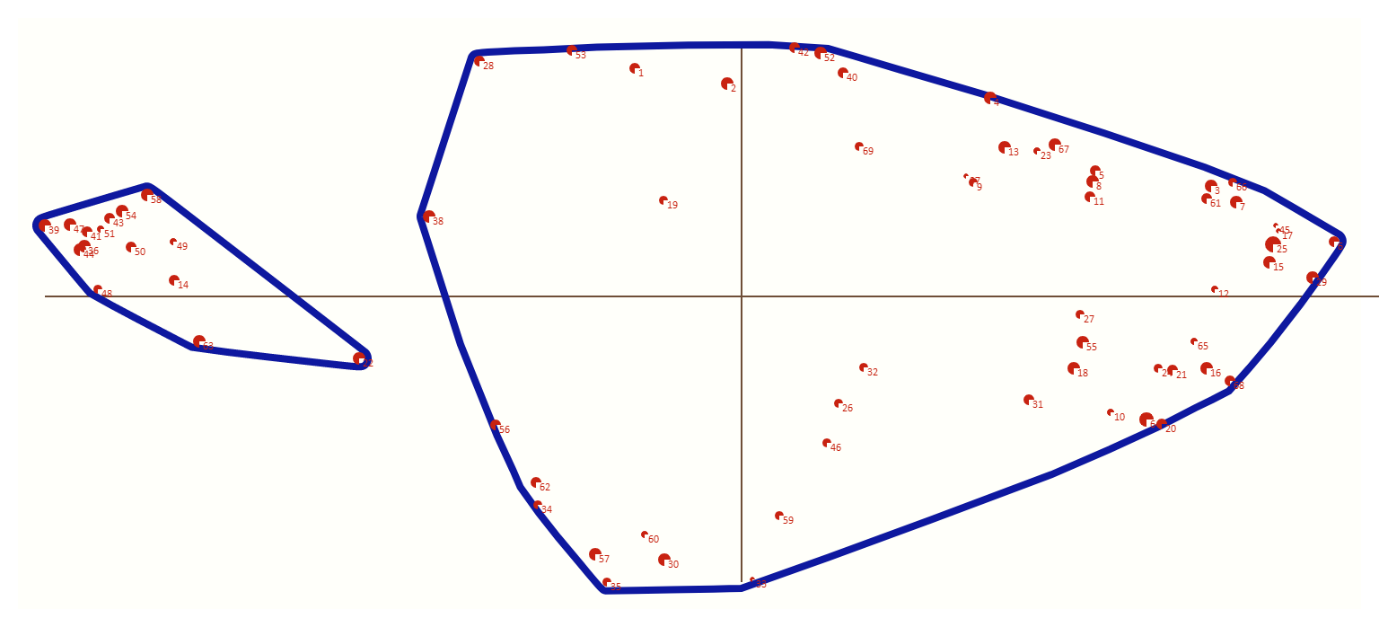


**Three-cluster solution**


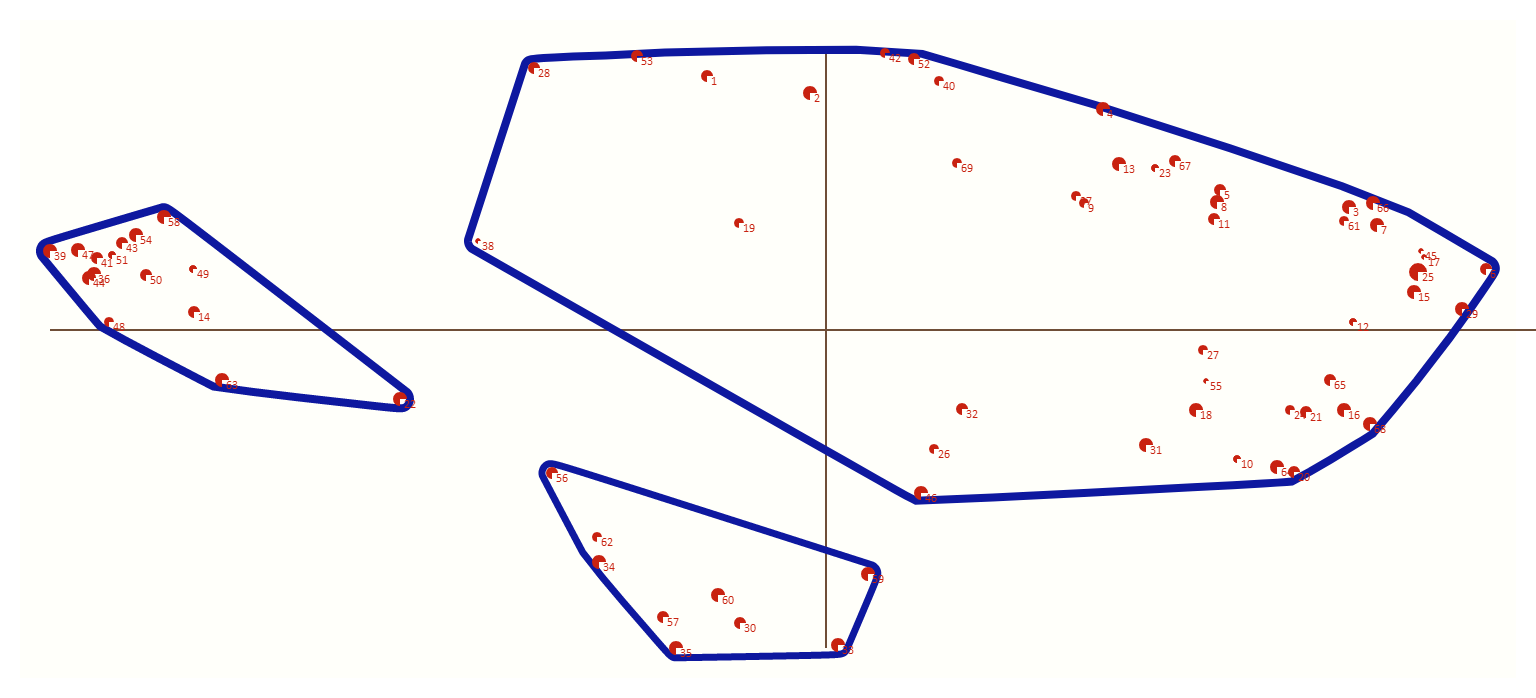


**Four-cluster solution**

**
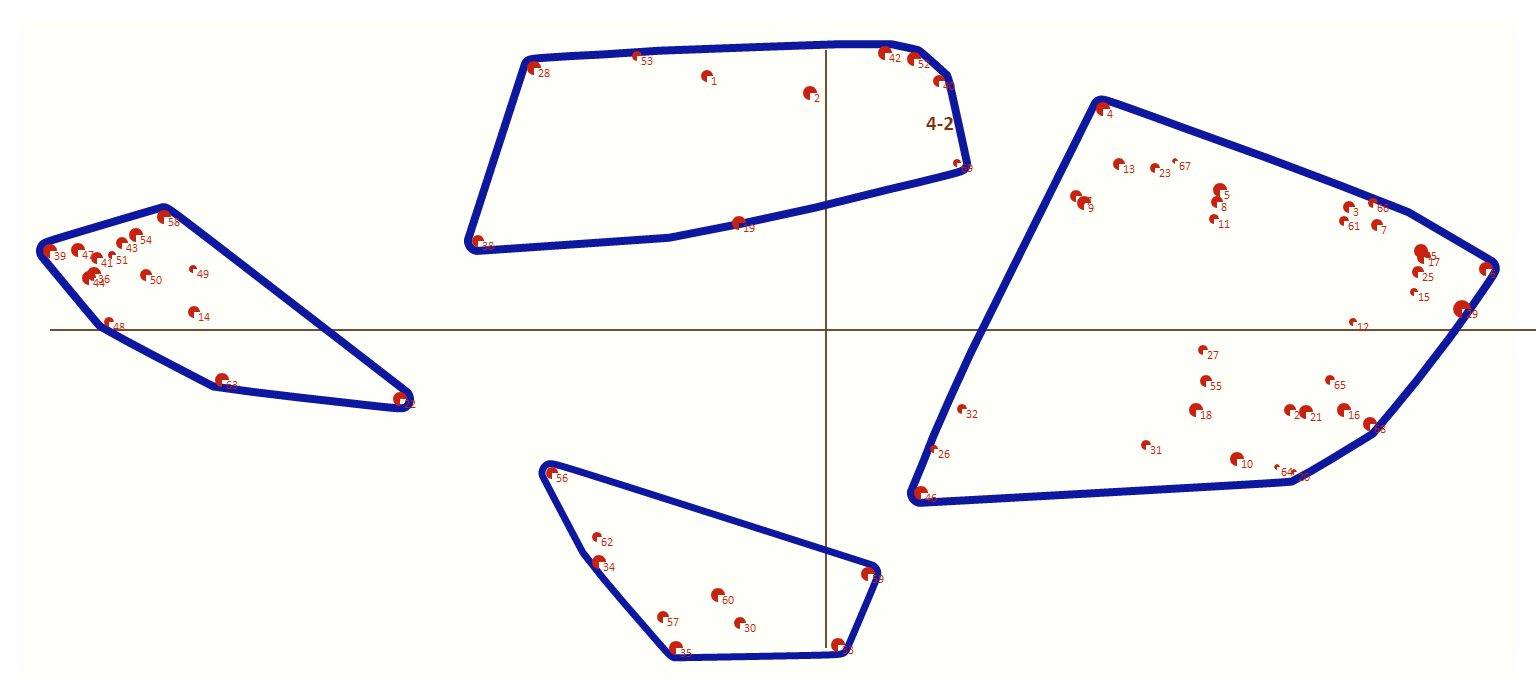
**

**Five-cluster solution**

**
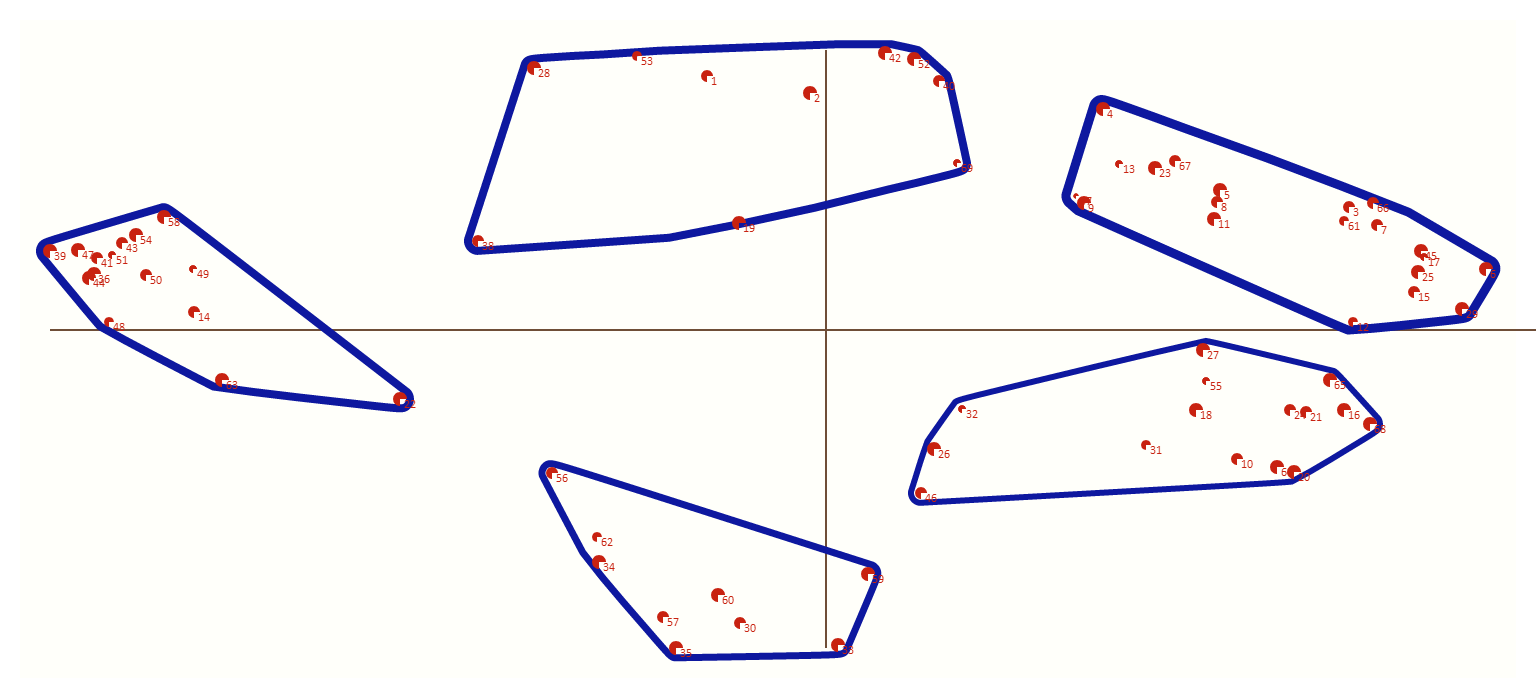
**

**Six-cluster solution**

**
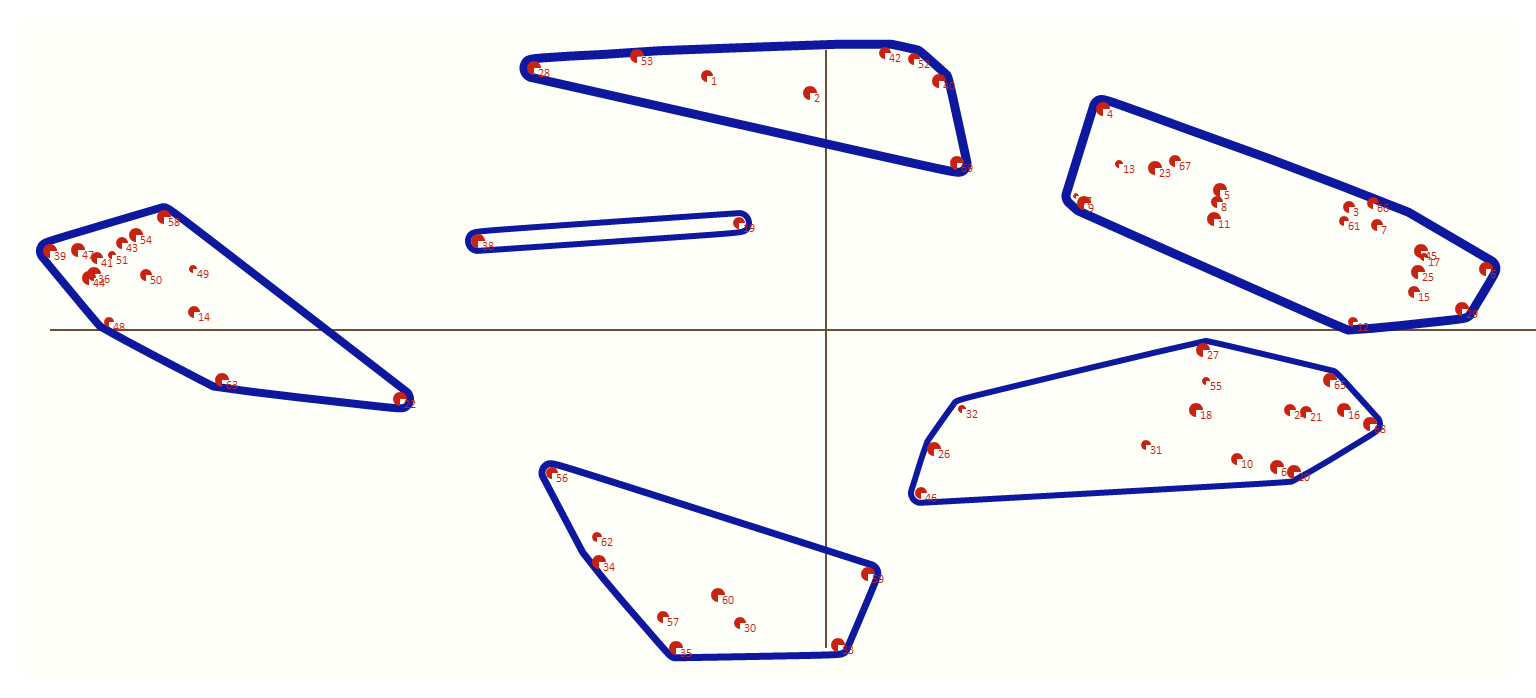
**

**Seven-cluster solution**

**
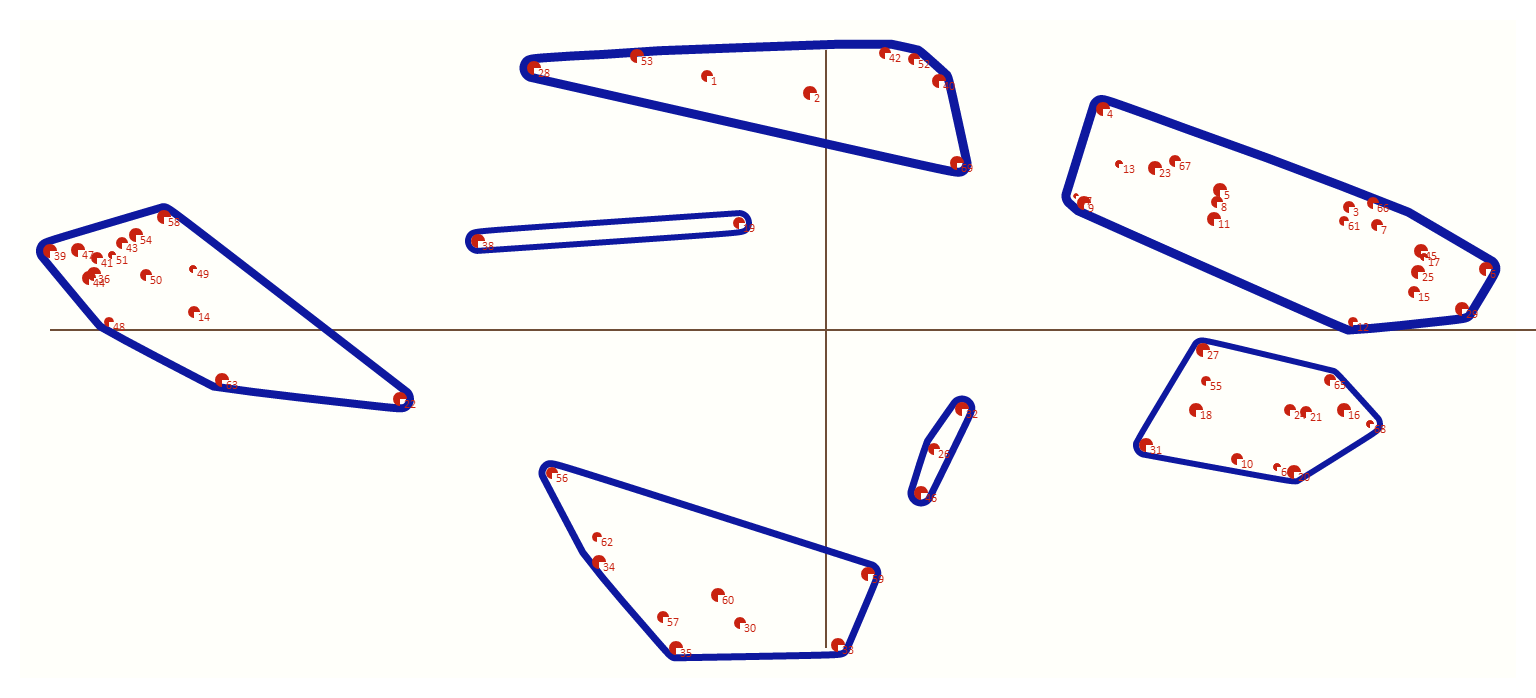
**

**Eight-cluster solution**

**
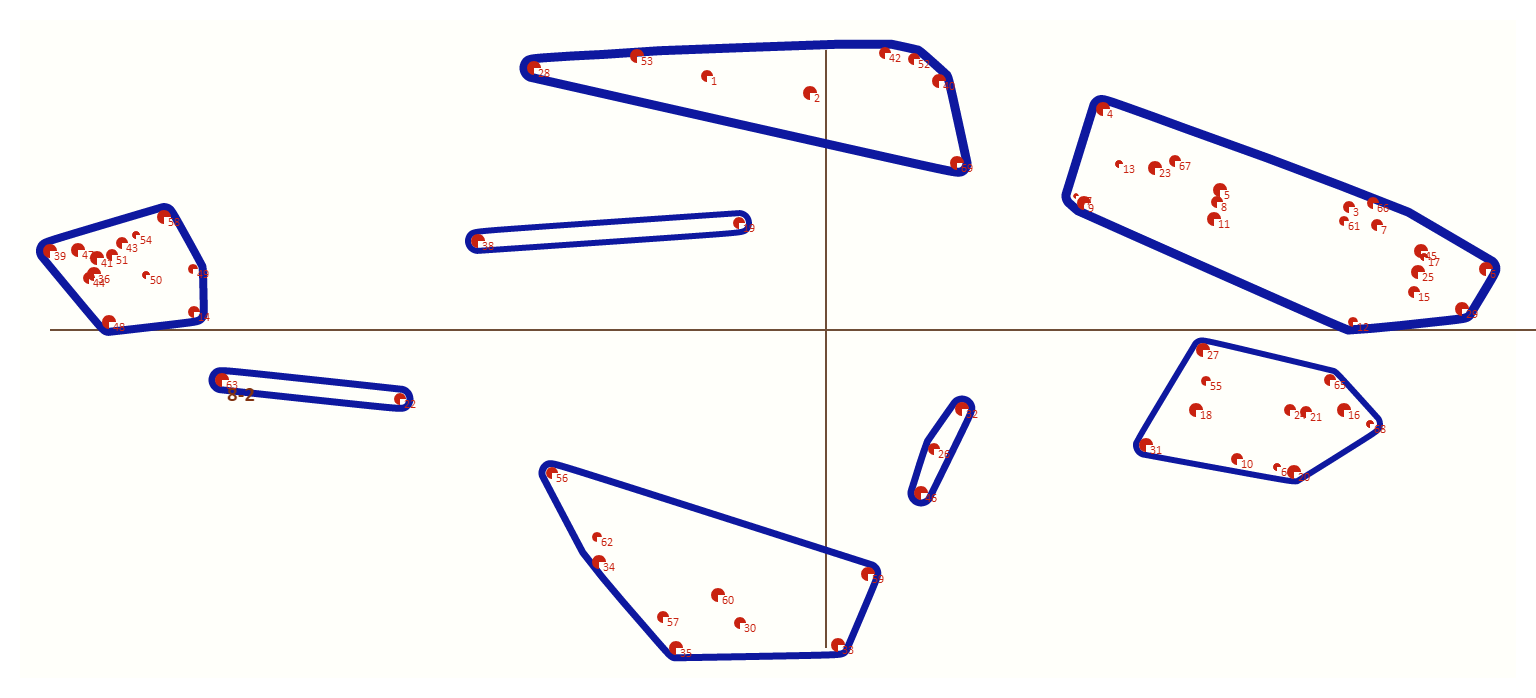
**

**Nine-cluster solution**

**
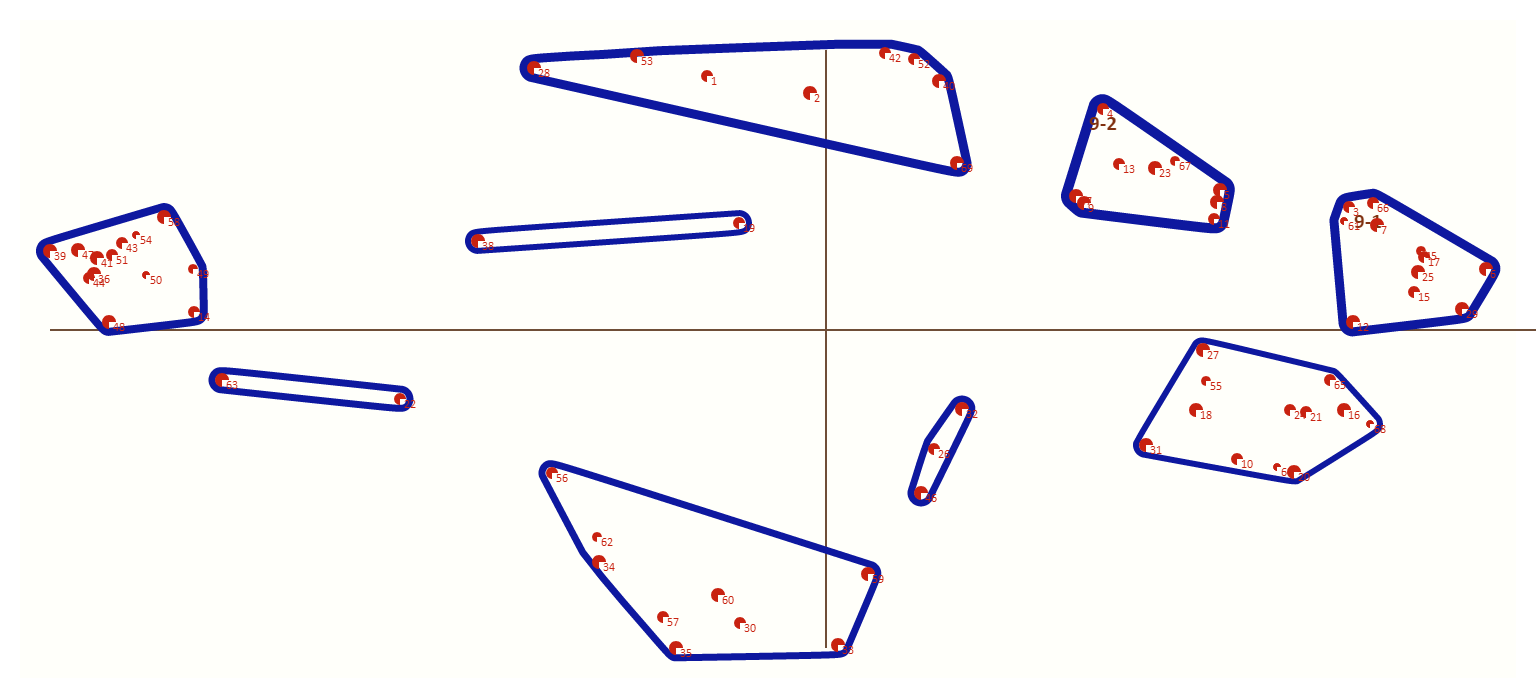
**

**Ten-cluster solution**

**
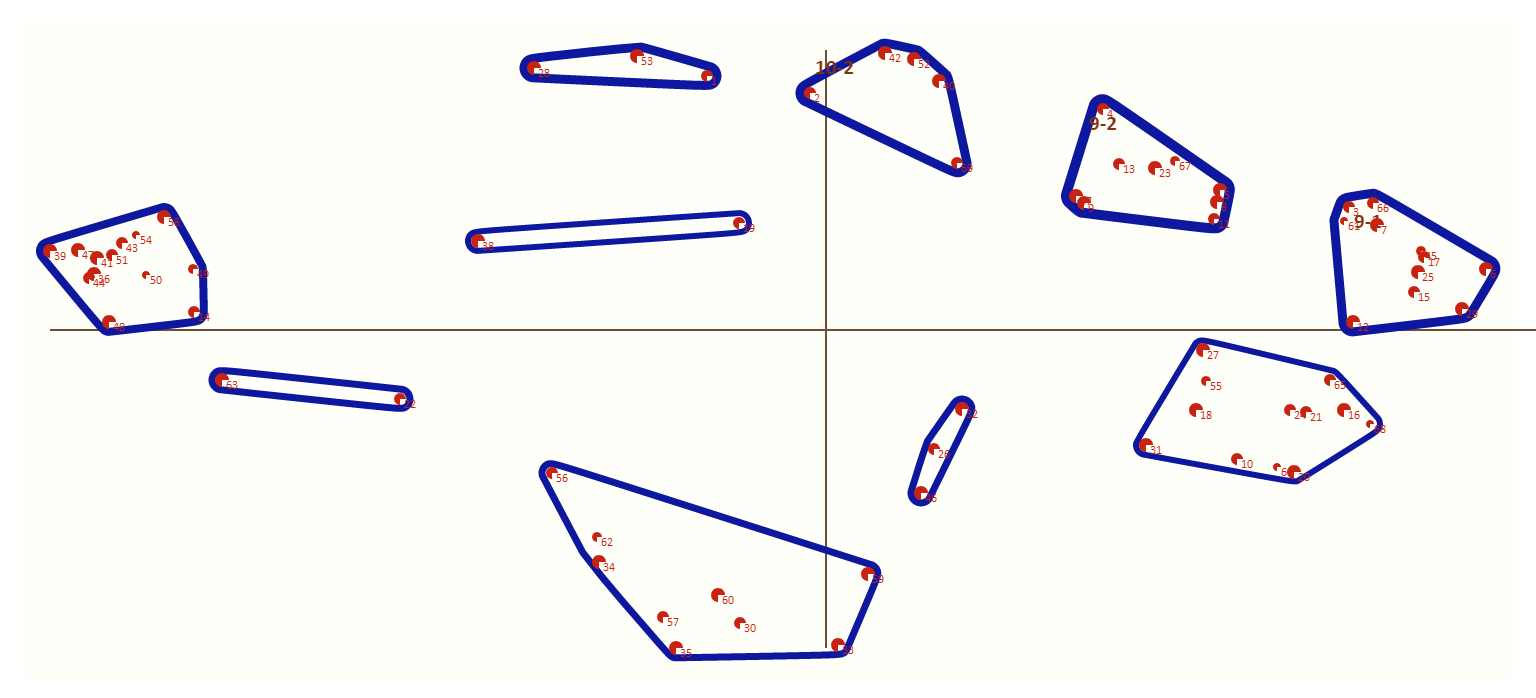
**

**Eleven-cluster solution**

**
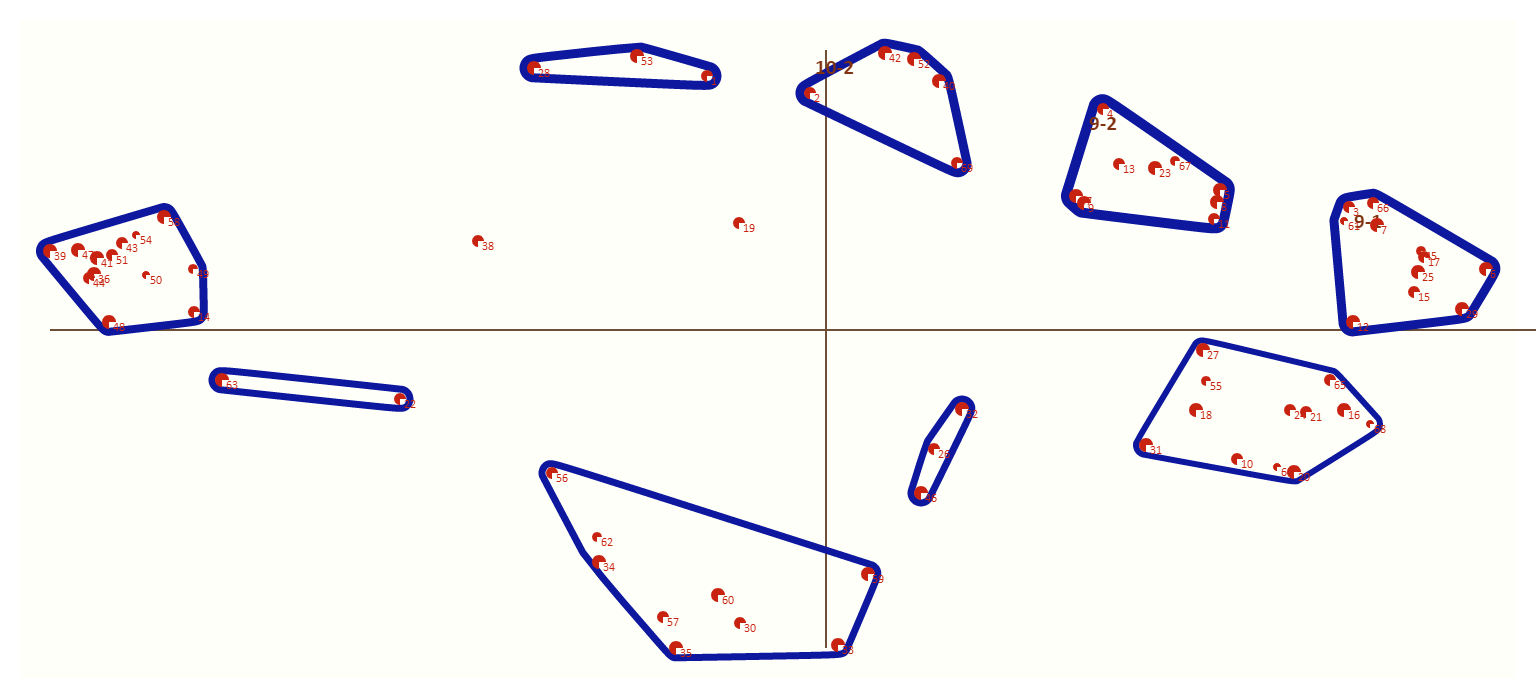
**

**Twelve-cluster solution**

**
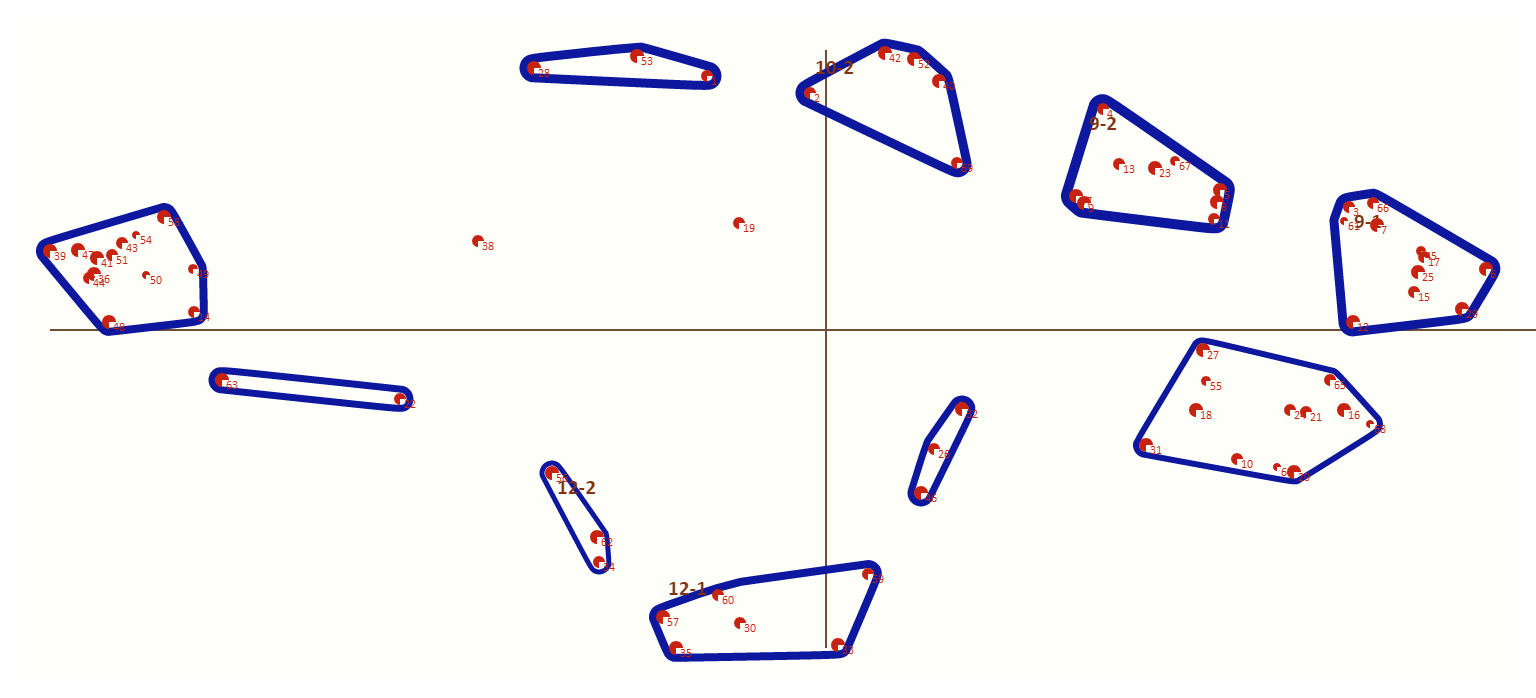
**

**Thirteen-cluster solution**

**
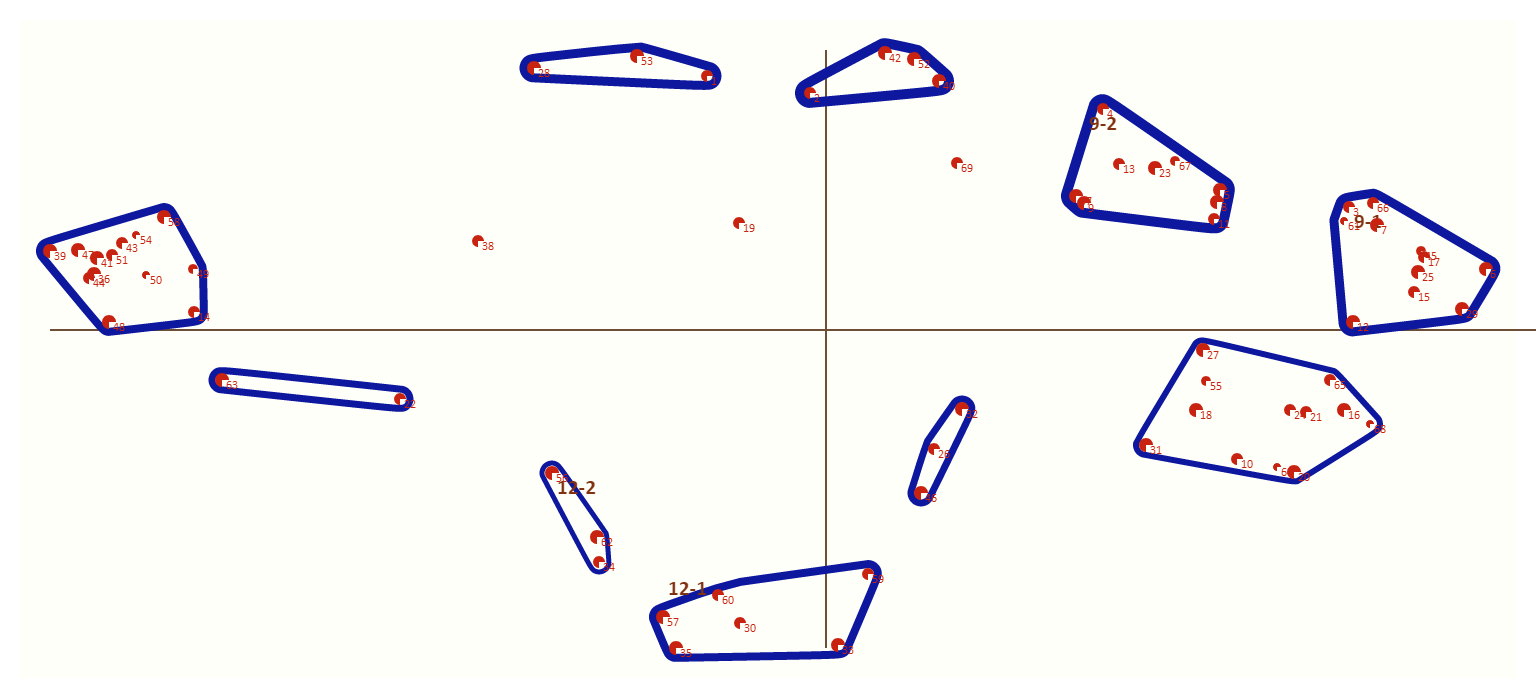
**

**Fourteen-cluster solution**

**
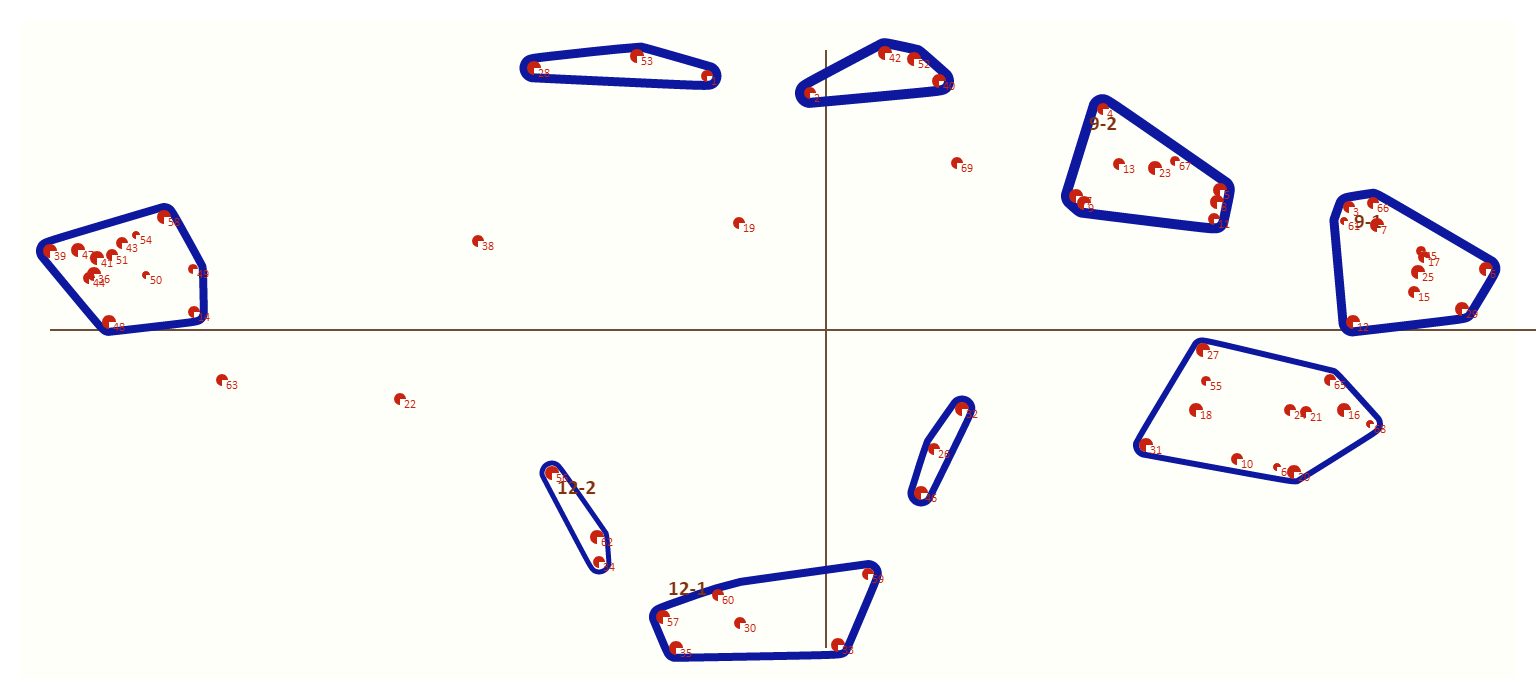
**

**Fifteen-cluster solution**

**
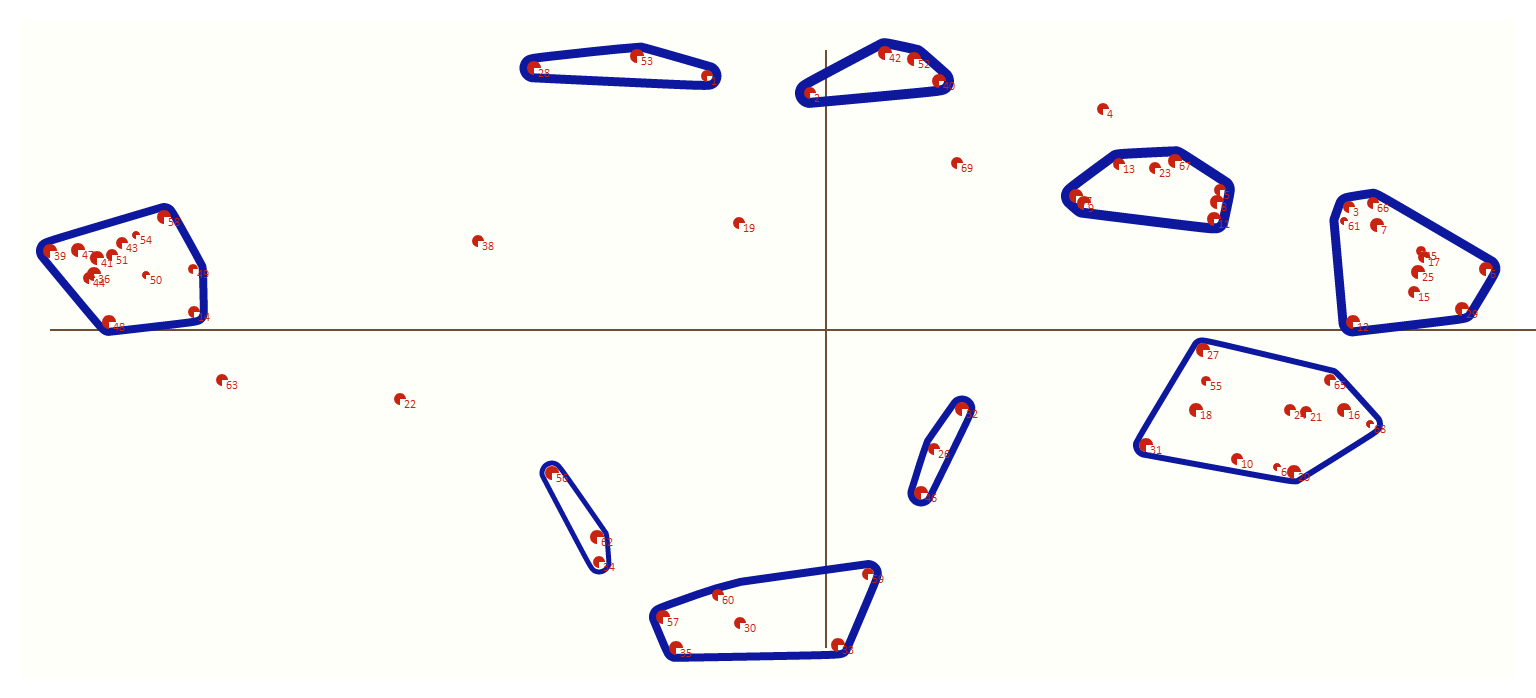
**

**Sixteen-cluster solution**

**
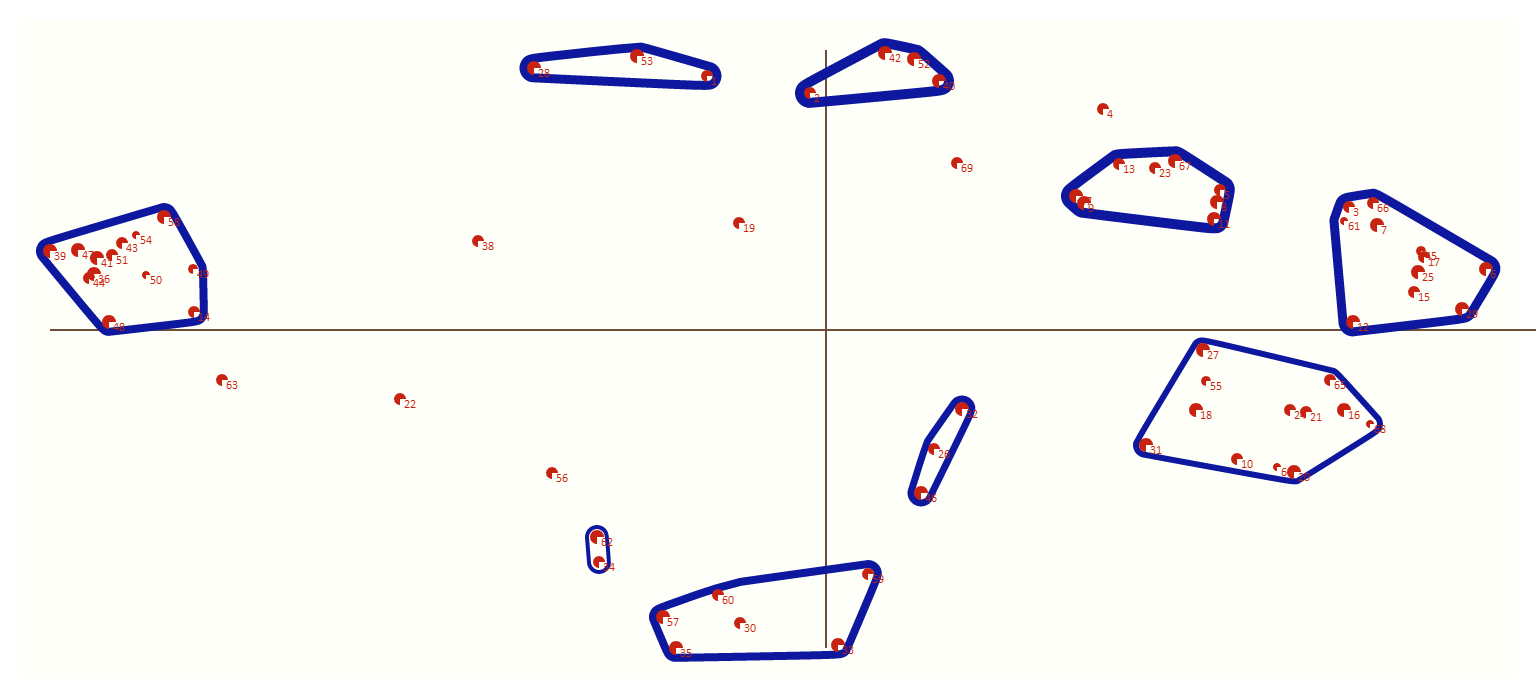
**

**Seventeen-cluster solution**

**
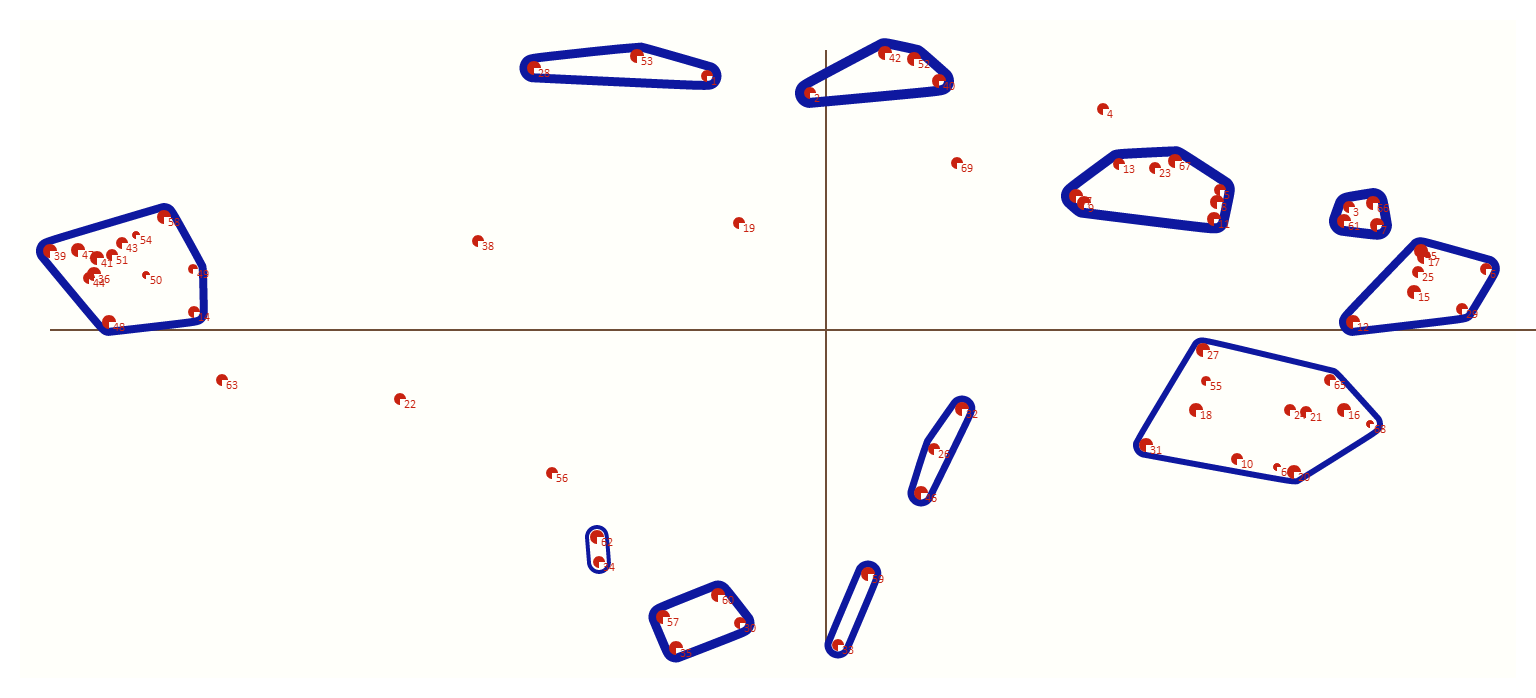
**

**Eighteen-cluster solution**

**
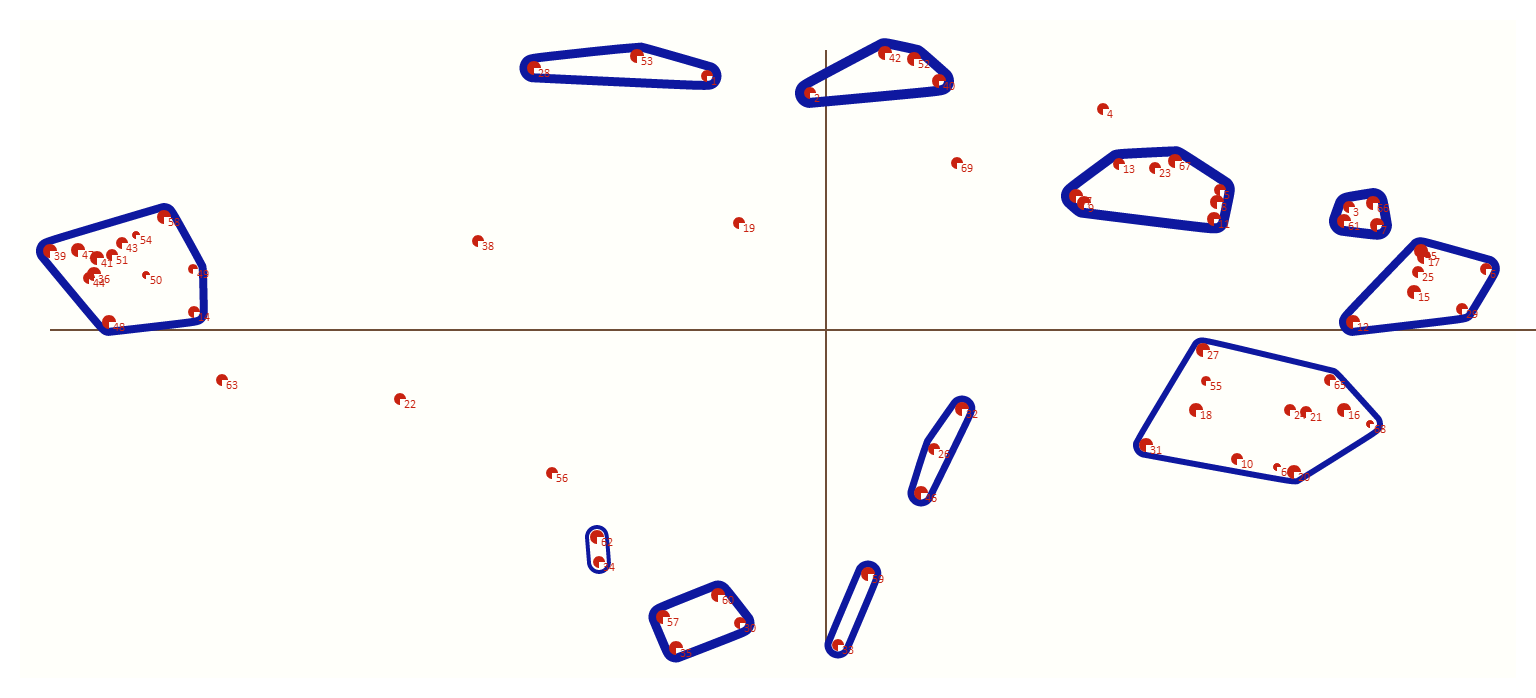
**
