# Supplementary figures and images for: Stakeholders’ Perceptions of How Nurse–Doctor Communication Impacts Patient Care: A Concept Mapping Study
Source: Nurs Rep. 2023 Nov 6;13(4):1607–23. doi: 10.3390/nursrep13040133 (PMC10661264; doi:10.3390/nursrep13040133)

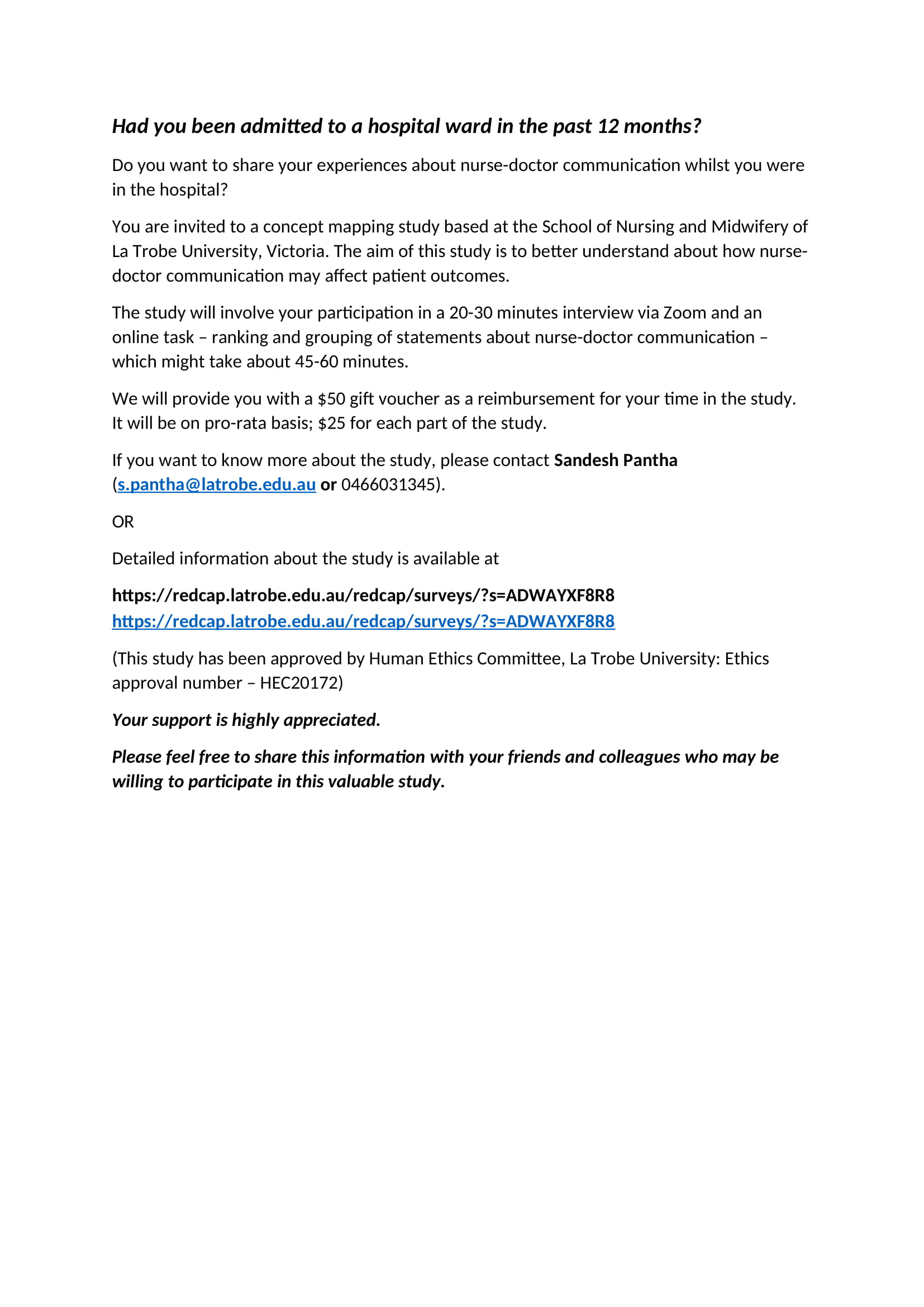


**Social media advert patient**


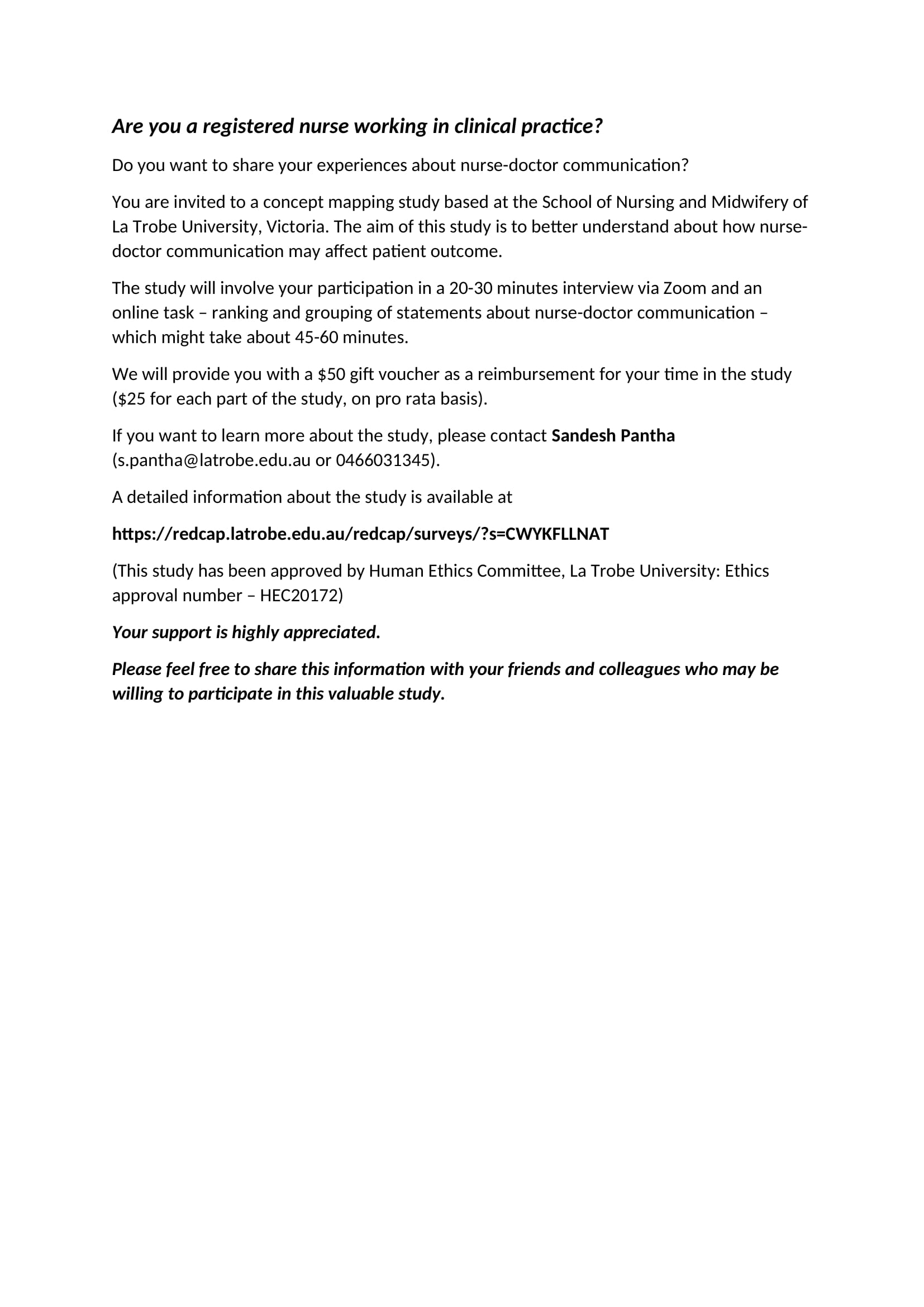


**Social media advert nurse**


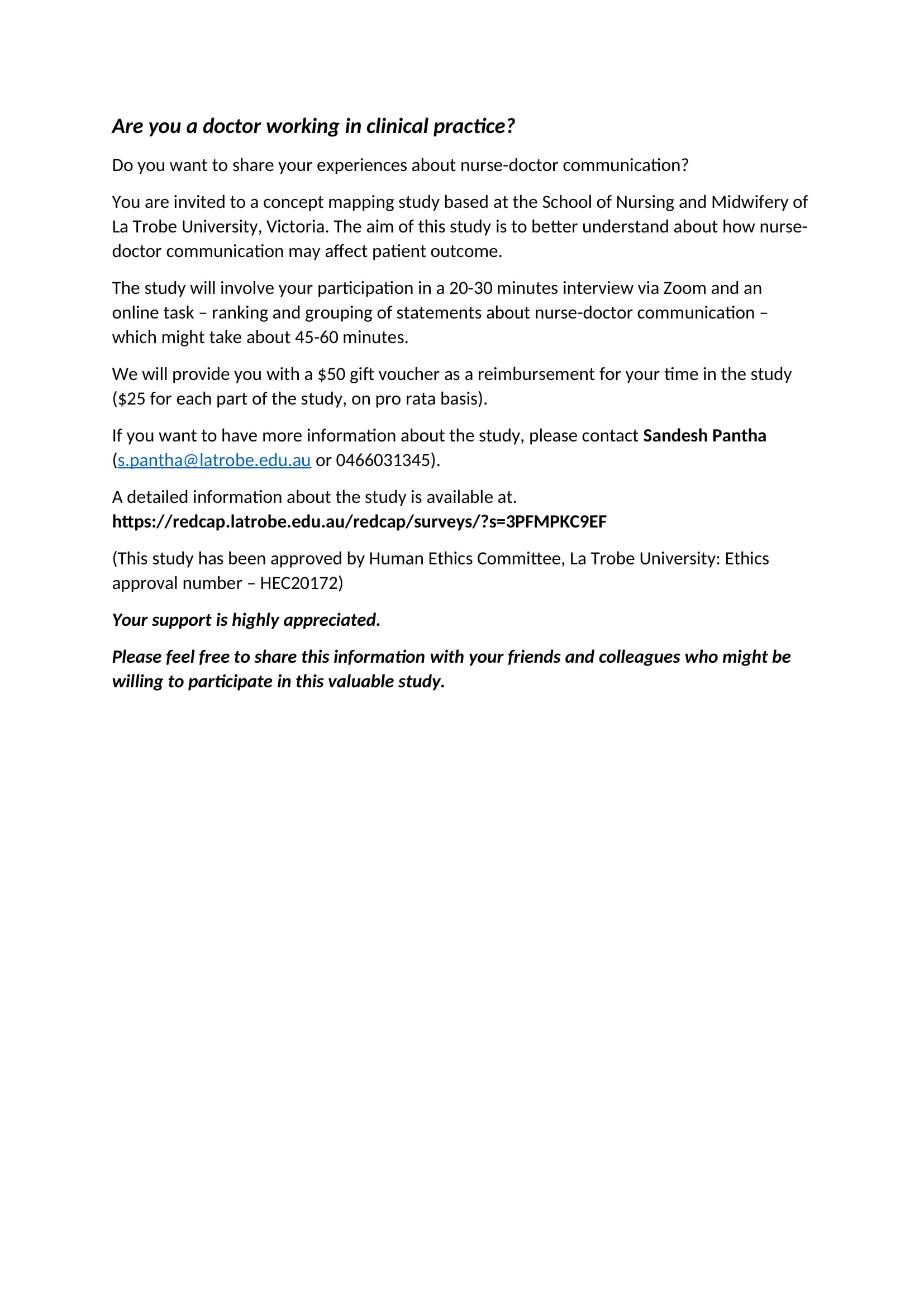


**Social media advert doctor**

Supplement: Supplementary file 1 [file nursrep-13-00133-s001.zip › nursrep-2581629-supplementary/S1_Social media advert for the study.docx]
